# Prevalence of neurotrophic tropomyosin receptor kinase (NTRK) fusion gene positivity in patients with solid tumors in Japan

**DOI:** 10.1002/cam4.7351

**Published:** 2024-06-25

**Authors:** Eiji Nakata, Tatsunori Osone, Toru Ogawa, Tomoyuki Taguchi, Kana Hattori, Shinji Kohsaka

**Affiliations:** ^1^ Department of Orthopedic Surgery Okayama University Okayama Japan; ^2^ Center for Comprehensive Genomic Medicine Okayama University Hospital Okayama Japan; ^3^ Faculty of Medicine, Dentistry and Pharmaceutical Sciences Okayama University Okayama Japan; ^4^ Medical Affairs & Pharmacovigilance Bayer Yakuhin, Ltd Osaka Japan; ^5^ National Cancer Center Research Institute Tokyo Japan

**Keywords:** comprehensive genomic profiling, neurotrophic tropomyosin receptor kinase (NTRK) gene fusion, next‐generation sequencing, solid tumors

## Abstract

**Background:**

Members of the neurotrophic tropomyosin receptor kinase (*NTRK*) gene family, *NTRK1*, *NTRK2*, and *NTRK3* encode TRK receptor tyrosine kinases. Intra‐ or inter‐chromosomal gene rearrangements produce *NTRK* gene fusions encoding fusion proteins which are oncogenic drivers in various solid tumors.

**Methods:**

This study investigated the prevalence of *NTRK* fusion genes and identified fusion partners in Japanese patients with solid tumors recorded in the Center for Cancer Genomics and Advanced Therapeutics database of comprehensive genomic profiling test.

**Results:**

In the analysis population (*n* = 46,621), *NTRK* fusion genes were detected in 91 patients (0.20%). The rate was higher in pediatric cases (<18 years; 1.69%) than in adults (0.16%). *NTRK* gene fusions were identified in 21 different solid tumor types involving 38 different partner genes including 22 (57.9%) previously unreported *NTRK* gene fusions. The highest frequency of *NTRK* gene fusions was head and neck cancer (1.31%) and thyroid cancer (1.31%), followed by soft tissue sarcoma (STS; 0.91%). A total of 97 *NTRK* fusion gene partners were analyzed involving mainly *NTRK1* (49.5%) or *NTRK3* (44.2%) gene fusions. The only fusion gene detected in head and neck cancer was *ETV6::NTRK3* (*n* = 22); in STS, *ETV6::NTRK3* (*n* = 7) and *LMNA::NTRK1* (n = 5) were common. Statistically significant mutual exclusivity of *NTRK* fusions with alterations was confirmed in *TP53*, *KRAS*, and *APC*. *NTRK* gene fusion was detected from 11 STS cases: seven unclassified sarcoma, three sarcoma NOS, and one Ewing sarcoma.

**Conclusions:**

*NTRK* gene fusion identification in solid tumors enables accurate diagnosis and potential TRK inhibitor therapy.

## Graphical abstract

In the C‐CAT database *NTRK* gene fusions were identified in 21 different solid tumor types. The highest frequency of *NTRK* gene fusions in patients by cancer type was head and neck cancer (22/1678, 1.31%) and thyroid cancer (7/534, 1.31%), followed by soft tissue sarcoma (STS) (19/2077, 0.91%). *NTRK* gene fusions were identified in several rare cancer types, including testis cancer (1/84), giving a prevalence of 1.19%. Identification of *NTRK* gene fusions in solid tumors with CGP testing enables an accurate diagnosis and TRK inhibitor treatment as a potential therapeutic option.

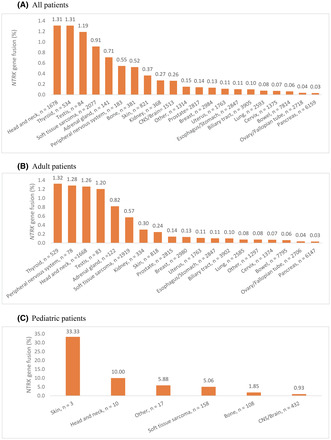

## 1 INTRODUCTION

Members of the neurotrophic tropomyosin receptor kinase (*NTRK*) gene family, *NTRK1*, *NTRK2*, and *NTRK3* encode the TRK receptor tyrosine kinases TRKA, TRKB, and TRKC, respectively. TRK receptors are activated by neurotrophins and are involved in the development and function of the central and peripheral nervous systems.^1^, ^2^, ^3^, ^4^, ^5^, ^6^

Intra‐ or inter‐chromosomal gene rearrangements involving *NTRK* genes produce gene fusions encoding fusion proteins which are oncogenic drivers of various adult and pediatric solid tumors. TRK fusion proteins are created following fusion of *NTRK* gene sequences encoding the C‐terminal kinase domain with sequences of various partner genes encoding N‐terminal polypeptides.^5^, ^6^ The prevalence of *NTRK* gene fusions in solid tumors is less than 1%, although some rare cancers such as infantile fibrosarcoma, and secretory carcinomas of the breast and salivary gland may have a much higher prevalence.^4^, ^7^, ^8^, ^9^, ^10^, ^11^ However, these reports are from Western countries and the prevalence of *NTRK* gene fusions in solid tumors is unknown in Asian individuals. Diagnostic testing for the identification of patients with TRK fusion cancer commonly uses next‐generation sequencing (NGS) based on RNA, DNA, or combined DNA/RNA methods.^12^ These methods are important for the identification of patients with *NTRK* fusion genes who may be eligible for appropriate treatment.^12^, ^13^ Two TRK inhibitors, larotrectinib and entrectinib have shown durable antitumor activity in clinical trials of TRK fusion‐positive cancer patients^14^, ^15^, ^16^ and have received regulatory approval in many countries including the United States, Europe, and Japan.^17^, ^18^

In Japan, data from comprehensive genomic profiling (CGP) are gathered in the Center for Cancer Genomics and Advanced Therapeutics (C‐CAT) database—the National Datacenter for Cancer Genomic Medicine. C‐CAT receives clinical information from hospitals specializing in cancer genomic medicine (CGM) and cancer genomic data from certified testing laboratories. Use of these data requires prior informed consent.^19^ Three genomic profiling platforms for CGP testing (FoundationOne® CDx, FoundationOne® Liquid CDx, and the OncoGuide™ NCC Oncopanel System) are covered by Japan's national health insurance system. For cancer types with standard treatment, CGP can be reimbursed at the time, or expected time of treatment completion.^20^, ^21^

In general, research on the prevalence of *NTRK* fusion genes in cancer is progressing. Although algorithms for identification of patients with *NTRK* fusion genes have been described,^5^, ^22^, ^23^ none have been accepted widely and prevalence data is subject to the range and type (e.g., RNA‐ and/or DNA‐based CGS) of methods used for genomic screening. Furthermore, no real‐world cross‐sectional studies of *NTRK* fusion genes by cancer type in Asia have been conducted. Previous large scale *NTRK* fusion gene testing studies in Japan, have focused on patients with lung cancer^24^ or colorectal adenocarcinomas.^25^ This is the first study which analyses *NTRK* fusion genes in multiple solid tumors in Asian patients. The aim of this study was to investigate the prevalence of *NTRK* fusion genes and identify fusion partners in Japanese patients with solid tumors recorded in the C‐CAT database, by cancer type. By verifying the detection rate of *NTRK* fusion genes, it may be possible to determine which patient groups require further testing.

## 2 MATERIALS AND METHODS

### 2.1 Study design

This is a retrospective descriptive observational study using the C‐CAT cancer knowledge database to retrieve CGP data from patients with solid tumors. Informed consent for secondary data use by other parties is obtained from patients in each hospital before each data transfer. Over 99% patients in C‐CAT have agreed to the secondary use of their genome and clinical information and all the available data in the dataset for this study are composed only from patients who provided informed consent for secondary data use.^26^

### 2.2 Patients and data retrieval

Patients diagnosed with a solid tumor who underwent CGP were included in the study. The study period was from 1 June 2019 to 23 February 2023. Diagnostic information, patient characteristics, specimen type, site and collection method, and pretreatment information were retrieved from records in the C‐CAT database. Histological information was classified according to OncoTree.^27^ Patients with a record of previous registration were excluded to avoid duplication.

### 2.3 Genomic profiling platforms

DNA‐based NGS CGP data were obtained using three gene profiling platforms: FoundationOne® CDx, FoundationOne Liquid® CDx, and the OncoGuide™ NCC Oncopanel System. Two CGP genomic platforms (FoundationOne CDx and OncoGuide NCC Oncopanel System) were covered by public health insurance in Japan in June 2019 while the third (FoundationOne Liquid CDx) received regulatory approval in Japan in March 2021 and is also covered by the national insurance system.^20^, ^21^ FoundationOne CDx and FoundationOne Liquid CDx analyze 324 genes in DNA isolated from solid tumors and blood samples (i.e., circulating cell‐free DNA), respectively. The OncoGuide NCC Oncopanel System version2 analyses 124 genes in DNA from solid tumors.^20^ FoundationOne CDx are designed to detect *NTRK1*, *NTRK2*, and *ETV6::NTRK3* fusion genes, while NCC Oncopanel can detect *NTRK1‐3* fusion genes. Data from each gene profiling platform were analyzed separately.

### 2.4 Endpoints

The primary endpoint was the proportion of *NTRK* fusion genes in all patients with solid tumors, and in adult (≥18 years) and pediatric (<18 years) patient groups. The prevalence of *NTRK* fusion genes was calculated for patient categories including gender, age group, cancer type, presence/absence of previous treatment, and type of cancer genome profiling panel used to identity fusion genes.

Exploratory endpoints were the proportion of fusion partner genes for *NTRK* 1, 2, and 3; the distribution of cancer types among *NTRK* fusion genes; association and co‐expression of *NTRK* fusion genes with other genetic abnormalities including high microsatellite instability (MSI), or high tumor mutational burden (TMB) (≥10 mutations/megabase); and correlation of the presence of *NTRK* fusion genes with other genetic abnormalities. The identified *NTRK* fusion pairs were confirmed by searching five websites: FusionGDB2 (https://compbio.uth.edu/FusionGDB2/index.html), TCGA Fusion Gene Database (https://www.tumorfusions.org/), FPIA (http://bioinfo‐sysu.com/fpia/#update), Mitelman Database of Chromosome Aberrations and Gene Fusions in Cancer (https://mitelmandatabase.isb‐cgc.org/mb_search), and COSMIC Fusions database (https://cancer.sanger.ac.uk/cosmic). In addition, searches of published articles in PubMed (https://pubmed.ncbi.nlm.nih.gov/) were conducted.

Previously unreported *NTRK* gene fusion partners were searched using eEnsembl (http://asia.ensembl.org/index.html) and for the presence of a coiled‐coil domain using the SMART (Simple Modular Architecture Research Tool) web‐based protein domain annotation resource (http://smart.embl.de/).

### 2.5 Statistical analyses

Categorical and continuous data were analyzed descriptively using summary statistics. Continuous data was analyzed by the number of missing and nonmissing values, mean, and standard deviation (SD), Categorical data were summarized by frequency and percentage. Prevalence of gene alterations including microsatellite instability (MSI) and tumor mutational burden (TMB) status were calculated, together with the associated odds ratio and Spearman's rank correlation coefficient between each gene alteration and *NTRK* fusion. All analyses were performed in a manner consistent with the Strengthening the Reporting of Observational Studies in Epidemiology (STROBE) guidelines^28^ and applicable sections of the Consolidated Standards of Reporting Trials (CONSORT) guidelines.^29^

All data analyses were performed using the SAS version 9.4 software package (SAS Institute Inc., Cary, NC, USA) conducted by Syneos Health Clinical.

## 3 RESULTS

A total of 46,745 records of patients with solid tumors were retrieved from the C‐CAT database. Patients with a record of previous registration were excluded to avoid duplication (*n* = 124) leaving 46,621 as the population for analysis.

Demographics and clinical characteristics of the whole patient cohort are summarized in Table 1. Patients included adults (*n* = 45,613; 97.8%) and children (*n* = 1008; 2.2%), with a mean (SD) age of 60.4 (14–74) years and about half were male (49.7%). Most patients (77.2%) had received chemotherapy previously. FoundationOne® CDx testing (75.3%) was the most commonly used platform for CGP analysis of solid tumors followed by FoundationOne® Liquid CDx (13.1%) and OncoGuide™ NCC Oncopanel System (11.6%). Specimens were mainly Formalin‐Fixed Paraffin‐Embedded (86.8%), collected following surgery (55.2%), and from a primary lesion (57.7%). Most cases (87.3%) had metastasis at the time of CGP analysis.

**TABLE 1:** Demographics and clinical characteristics in all patients and *NTRK* fusion gene positive patients.

Parameter		Database population			*NTRK* fusion gene positive		
		All (*N* = 46,621)	Adult (*N* = 45,613)	Pediatric (*N* = 1008)	All (*N* = 91)	Adult (*N* = 74)	Pediatric (*N* = 17)
Age (years), mean (SD)		60.37 (14.7)	61.51 (12.7)	8.85 (5.2)	48.95 (25.8)	59.14 (15.7)	4.59 (5.9)
Gender	Male	23,157 (49.7)	22,647 (49.7)	510 (50.6)	50 (55.0)	40 (54.1)	10 (58.8)
	Female	23,459 (50.3)	22,962 (50.3)	497 (49.3)	41 (45.1)	34 (45.95)	7 (41.2)
	Missing data	5 (0.0)	4 (0.0)	1 (0.1)	0 (0)	0 (0)	0 (0)
Prior chemotherapy	Yes	35,984 (77.2)	35,391 (77.6)	593 (58.8)	52 (57.1)	42 (56.8)	10 (58.8)
	No	1682 (3.6)	1521 (3.33)	161 (15.97)	20 (21.98)	17 (22.97)	3 (17.65)
	Missing data	8955 (19.2)	8701 (19.1)	254 (25.2)	19 (20.9)	15 (20.3)	4 (23.5)
Genomic profiling panel	Foundation One® CDx	35,099 (75.3)	34,268 (75.1)	831 (82.4)	77 (84.6)	61 (82.4)	16 (94.1)
	Foundation One® Liquid CDx	6117 (13.1)	6075 (13.3)	42 (4.2)	6 (6.6)	6 (8.1)	0 (0)
	OncoGuide^TM^ NCC Oncopanel System	5405 (11.6)	5270 (11.6)	135 (13.4)	8 (8.8)	7 (9.5)	1 (5.9)
Specimen type	FFPE	40,482 (86.8)	39,520 (86.6)	962 (95.44	84 (92.3)	67 (90.5)	17 (100)
	Fresh frozen tissue	42 (0.1)	39 (0.1)	3 (0.3)	0 (0)	0 (0)	0 (0)
	Peripheral blood	6082 (13.1)	6041 (13.2)	41 (4.1)	6 (6.6)	6 (8.1)	0 (0)
	Other	15 (0.0)	13 (0.0)	2 (0.2)	1 (1.10	1 (1.4)	0 (0)
	Biopsy	14,639 (31.4)	14,286 (31.3)	353 (35.0)	28 (30.8)	22 (29.7)	6 (35.3)
	Surgery	25,720 (55.2)	25,110 (55.1)	610 (60.5)	57 (62.6)	46 (62.2)	11 (64.7)
Specimen collection method	Other	137 (0.3)	136 (0.3)	1 (0.1)	0 (0)	0 (0)	0 (0)
	Unknown	8 (0.0)	6 (0.0)	2 (0.2)	0 (0)	0 (0)	0 (0)
	Missing data	6117 (13.1)	6075 (13.3)	42 (4.2)	6 (6.6)	6 (8.1)	0 (0)
Specimen collection site	Primary lesion	26,916 (57.7)	26,155 (57.3)	761 (75.5)	57 (62.6)	41 (55.4)	16 (94.1)
	Metastatic lesion	13,372 (28.7)	13,172 (28.9)	200 (19.8)	28 (30.8)	27 (36.5)	1 (5.9)
	Unknown	216 (0.5)	211 (0.5)	5 (0.5)	0 (0)	0 (0)	0 (0)
	Missing data	6117 (13.2)	6075 (13.3)	42 (4.2)	6 (6.6)	6 (8.1)	0 (0)
Metastasis before CGP	Yes	40,708 (87.3)	40,225 (88.2)	483 (47.9)	63 (69.2)	59 (79.7)	4 (23.5)
	No	4006 (8.6)	3510 (7.7)	496 (49.2)	19 (20.9)	7 (9.5)	12 (70.6)
	Unknown	241 (0.5)	234 (0.5)	7 (0.7)	1 (1.1)	1 (1.4)	0 (0)
	Missing data	1666 (3.6)	1644 (3.6)	22 (2.2)	8 (8.8)	7 (9.5)	1 (5.9)

*Note*: Data are shown as *n* (%) unless stated otherwise.

Abbreviations: CGP, comprehensive genomic profiling; FFPE, formalin‐fixed paraffin‐embedded.

Overall, *NTRK* fusion genes were detected in 91 patients: in 74 adults (81.3%) and 17 pediatric cases (18.7%) (Table 1). The overall detection rate was 0.20% and rates were higher in children (1.69%) than adults (0.16%). *NTRK* rearrangements in which a fusion partner could not be confirmed or other gene rearrangements (including translocations and inversions) were detected in fewer patients (*n* = 40, 0.1%), with most occurring in adults (*n* = 39) (Table S1).

**FIGURE 1:**
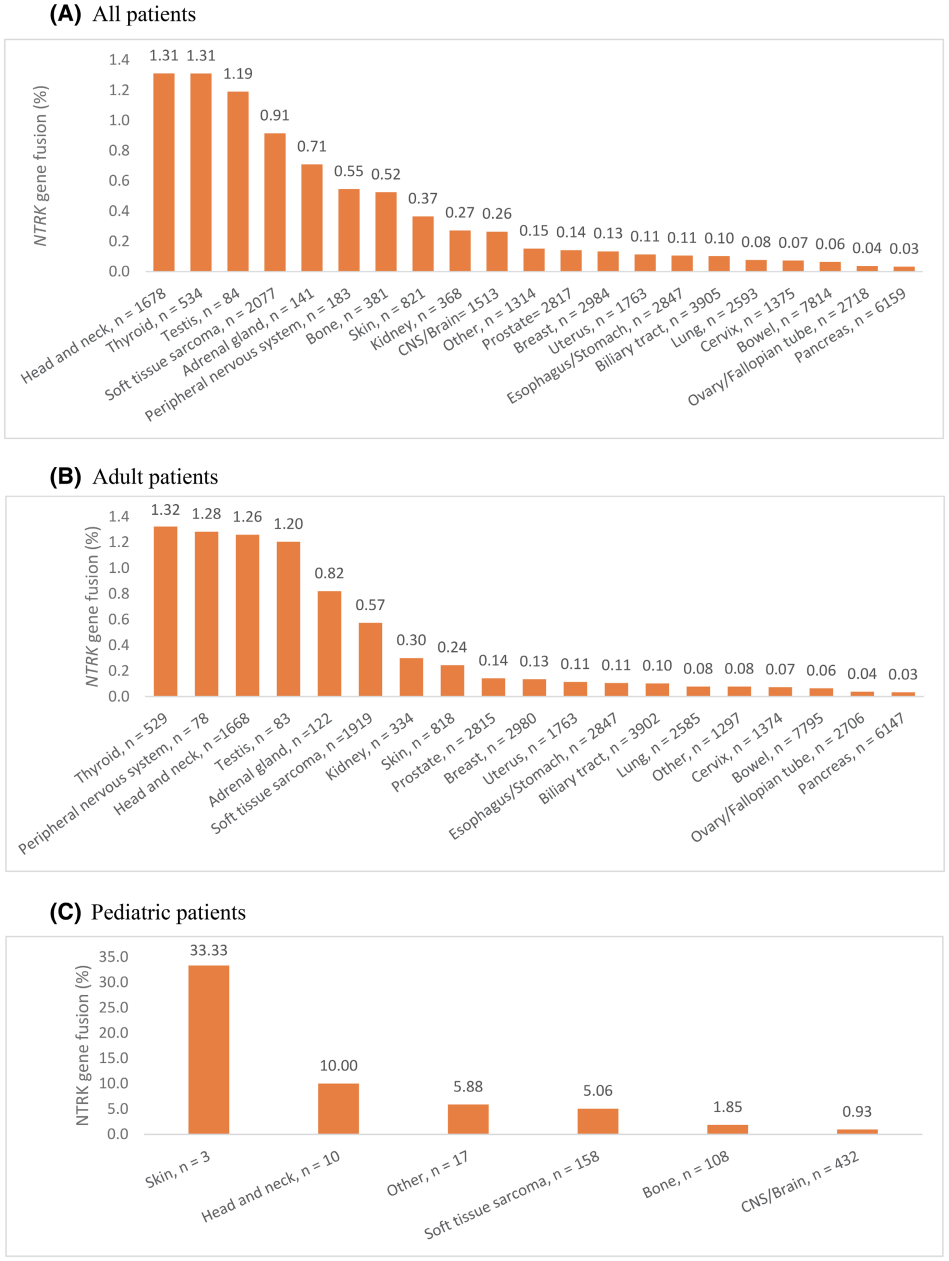
Proportion of *NTRK* gene fusions by cancer type in: (A) all patients; (B) adults (≥18 years) and (C) children (<18 years). The total number of patients by cancer type is shown on the x axis. (A) All patients. (B) Adult patients. (C) Pediatric patients.

*NTRK* gene fusions were identified in 21 different solid tumor types. The highest frequency of *NTRK* gene fusions in patients by cancer type (≥100 patients) was head and neck cancer (22/1678, 1.31%) and thyroid cancer (7/534, 1.31%), followed by soft tissue sarcoma (STS) (19/2077, 0.91%). *NTRK* gene fusions were identified in several rare cancer types, including testis cancer (1/84), giving a prevalence of 1.19% (Figure 1A). In adults, a relatively high prevalence was found in thyroid cancer (7/529, 1.32%), head and neck cancer (21/1668, 1.26%), and STS (11/1919, 0.57%). Single *NTRK* gene fusions were found in rare cancers, including peripheral nervous system cancer (1/78, 1.28%) and testis cancer (1/83, 1.20%) (Figure 1B). Of common cancers in children (≥100 patients), STS (8/158, 5.06%) and bone sarcoma (2/108, 1.85%) had the highest prevalence of *NTRK* gene fusions (Figure 1C).

**FIGURE 2:**
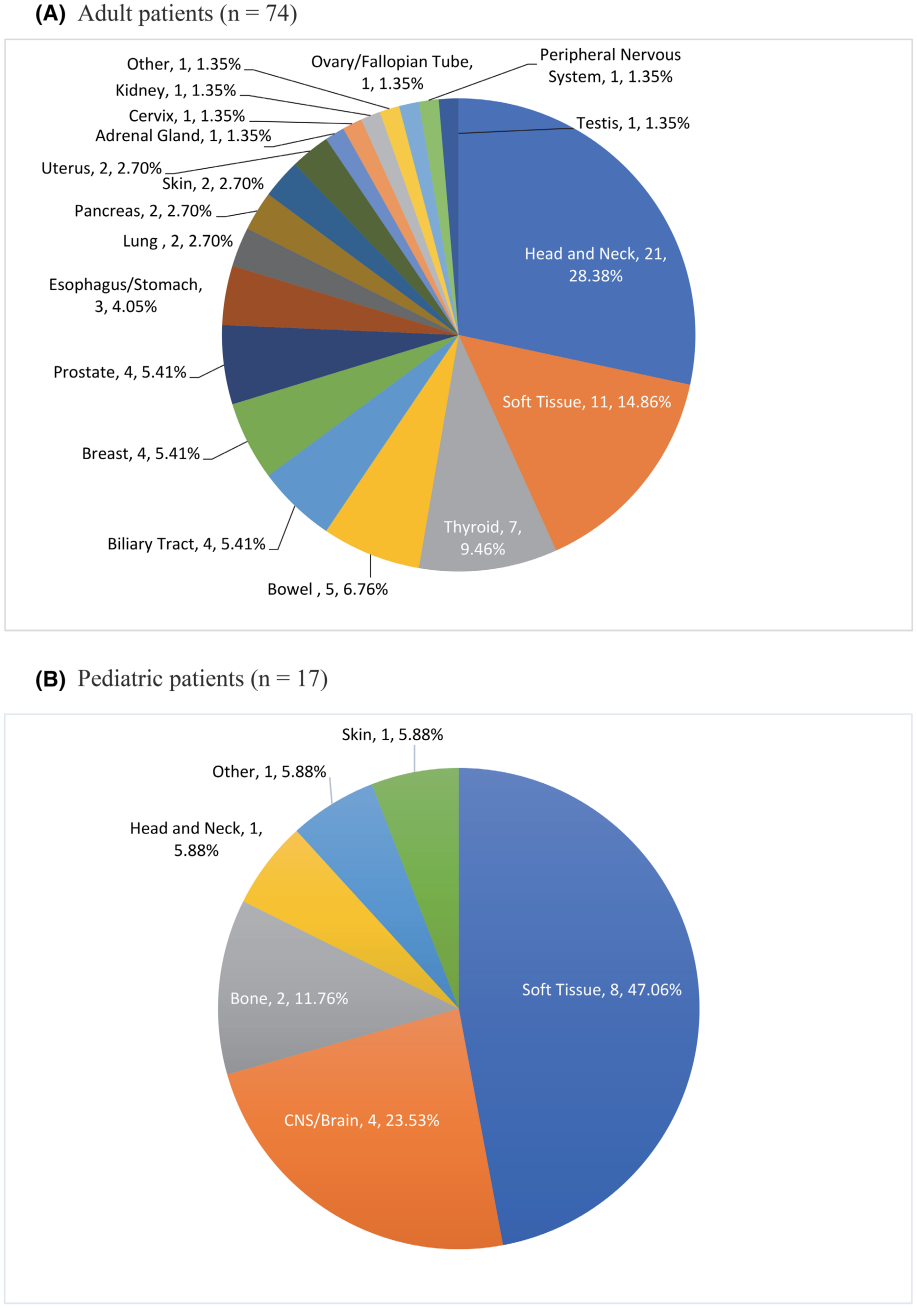
Number and proportion of cancer types in *NTRK* fusion gene positive adult and pediatric patients. (A) Adult patients. (B) Pediatric patients.

In adults with *NTRK* fusion gene positivity (*n* = 74), head and neck cancer (*n* = 21, 28.38%) was the most frequent cancer type followed by STS (*n* = 11, 14.86%), and thyroid cancer (*n* = 7, 9.46%) (Figure 2A). In pediatric cases with a detectable *NTRK* fusion gene (*n* = 17), the most frequent cancer type was STS (*n* = 8, 47.06%) then CNS/brain cancer (*n* = 4, 23.53%) (Figure 2B). Most STS with a detectable *NTRK* gene fusion (*n* = 19) were unclassified sarcoma (*n* = 7, 36.84%), followed by infantile fibrosarcoma (*n* = 3, 15.79%) and sarcoma/not otherwise specified (NOS; *n* = 3, 15.79%). The most common head and neck cancer with *NTRK* fusion gene positivity (*n* = 22) was salivary gland carcinoma (*n* = 16, 72.73%) and the remainder (*n* = 6, 27.27%) were unclassified.

Detection of *NTRK* gene fusions varied according to the platform used. Positivity rates were highest for FoundationOne® CDx (0.22%), then the OncoGuide™ NCC Oncopanel System (0.15%) and FoundationOne® Liquid CDx (0.10%) (Table S2).

**FIGURE 3:**
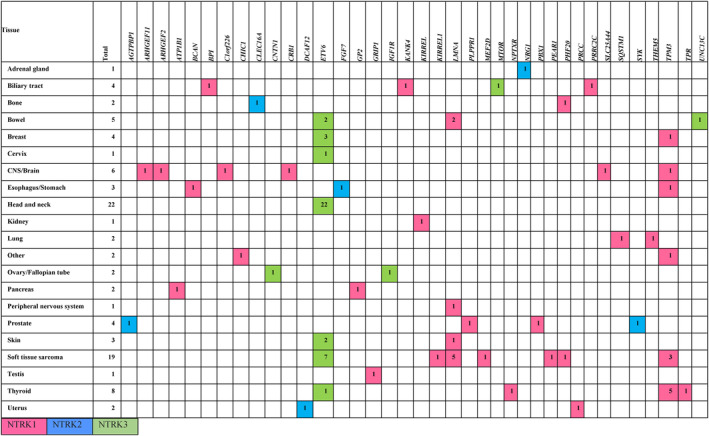
NTRK fusion gene (*NTRK1*, *NTRK2*, or *NTRK3*) partners by cancer type.

Overall, 97 *NTRK* fusion genes were detected in 91 cases. These included detection of *NTRK* gene fusions involving two partner genes in four subjects, and reciprocal translocations (*ETV6::NTRK3* and *NTRK3::ETV6*) in two subjects (Table S3). Most fusion gene partners were identified for *NTRK1* (*n* = 47; 49.5%), followed by *NTRK3* (*n* = 42; 44.2%) and *NTRK2* (*n* = 6; 6.3%). The only *NTRK* fusion gene detected in head and neck cancer was *ETV6::NTRK3* (*n* = 22). The most common fusion genes detected in STS were *ETV6::NTRK3* (*n* = 7) and *LMNA::NTRK1* (*n* = 5). In thyroid cancer, *TPM3::NTRK1* (*n* = 5) was the most frequently detected fusion gene (Figure 3).

**FIGURE 4:**
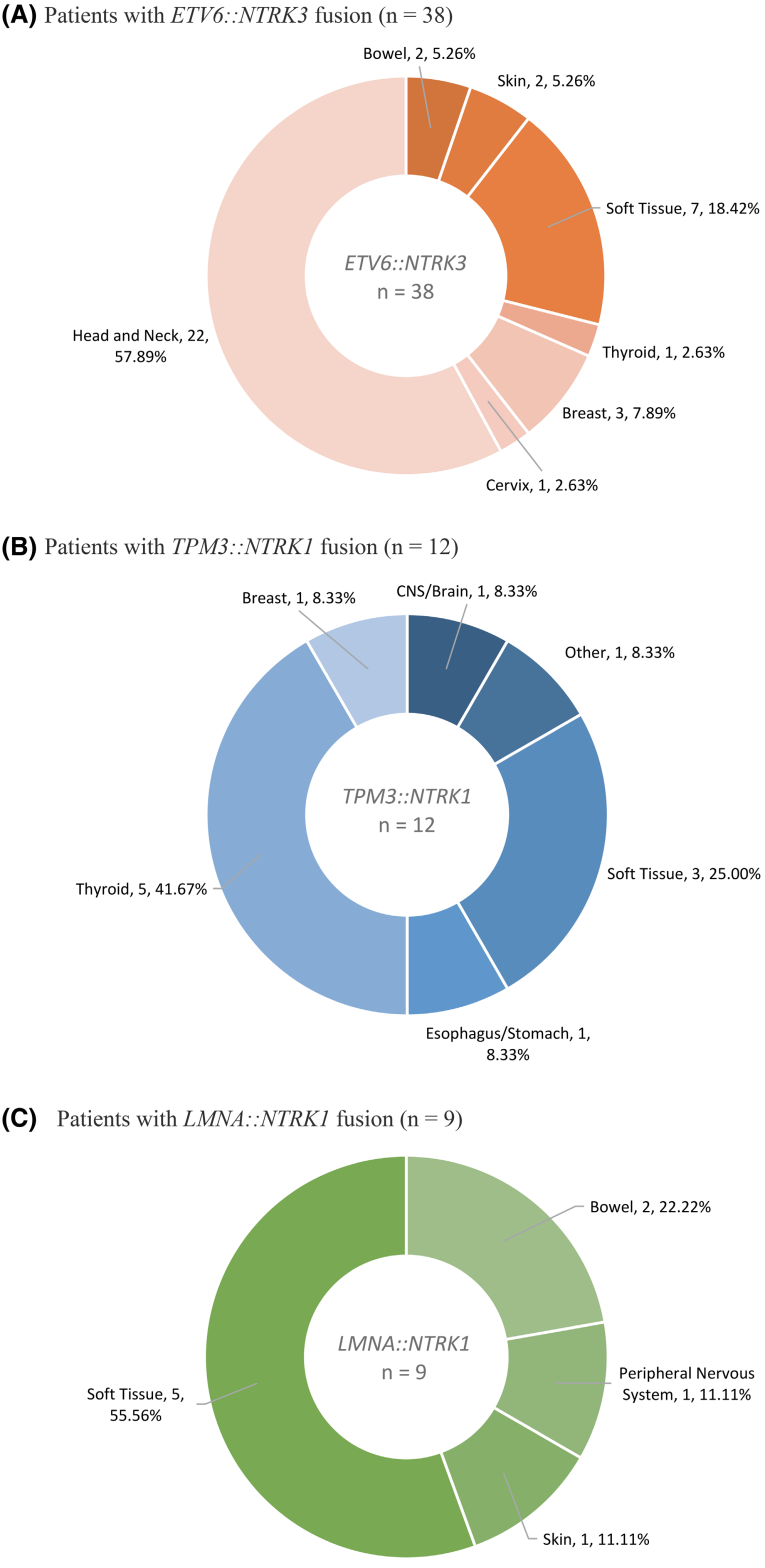
Frequency of common *NTRK* gene fusions by cancer type. (A) Patients with *ETV6::NTRK3* fusion (*n* = 38). (B) Patients with *TPM3::NTRK1* fusion (*n* = 12). (C) Patients with *LMNA::NTRK1* fusion (*n* = 9)

Distribution of the most common *NTRK* fusion genes by cancer type is shown in Figure 4. Of 38 *ETV6::NTRK3* gene fusions detected, most were found in head and neck cancers (*n* = 22; 57.9%) followed by STS (*n* = 7; 18.4%). Most *TPM3::NTRK1* gene fusions (*n* = 12) were found in thyroid cancer (*n* = 5, 41.7%), then STS (*n* = 3, 25.0%). Over half of *LMNA::NTRK1* gene fusions (*n* = 9) were detected in STS (*n* = 5, 55.6%).

**FIGURE 5:**
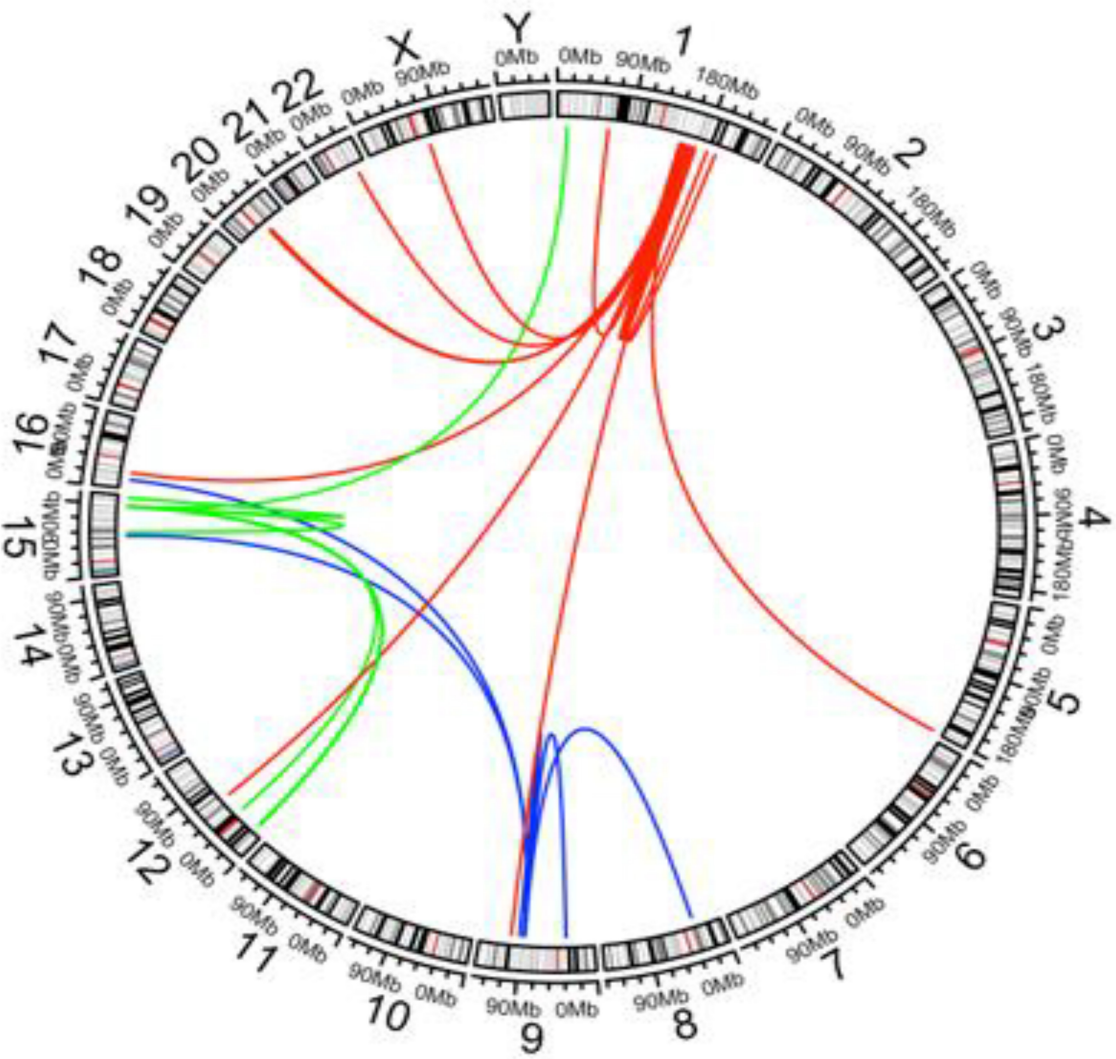
Circos plot depicting *NTRK* gene fusions by chromosomal location: gene fusions involving *NTRK1* (1q23.1; shown in red), *NTRK2* (9q21.33; shown in blue), and *NTRK3* (15q25.3; shown in green).

A total of 38 different fusion gene partners of *NTRK1*, *NTRK2*, and *NTRK3* were identified, of which 22 (57.9%) had not been reported previously (Table S4). Of 22 previously unreported fusion partners, 4 (18.2%: *CHIC1*, *KANK4*, *NPTXR*, and *PRRC2C*) contained a coiled‐coil domain, which leads to dimerization of the *NTRK* gene. The chromosomal locations of *NTRK* gene fusions are depicted diagrammatically in Figure 5 and indicate both intra‐and inter‐chromosomal rearrangements. *NTRK1* is localized on the long arm of chromosome (1q23.1) and fusion partners were localized on the short arm of chromosome 1 (1p) and multiple locations on the long arm (1q), other autosomal chromosomes (5q, 9q, 12q, 16p, 20q, and 22q), and the long arm of the X chromosome (Xq). Fusion partners of *NTRK2* which is localized on the long arm of chromosome 9 (9q21.33) were on chromosome 9p, 9q, 8p, 15q, and 16p. *NTRK3* is located on the long arm of chromosome 15 (15q25.3) and its most common fusion partner, *ETV6* was on chromosome 12p. Other fusion partners of *NTRK3* were on chromosome 1p, 12q, and 15q.

*NTRK* gene fusion‐positivity was not associated with high MSI or high TMB status. Of 91 patients who had a detectable *NTRK* gene fusion, three patients (3.3%) had high MSI and eight patients (8.8%) had high TMB (Table S5). Correlations between *NTRK* gene fusion‐positivity and MSI‐high or TMB‐high status were not statistically significant (Table S6). *NTRK* gene fusion‐positivity showed a very weak correlation with other biomarkers (*n* = 320). Statistically significant mutual exclusivity of *NTRK* fusions with alterations in *TP53* (*p* < 0.001), *KRAS* (*p* < 0.001), *APC* (*p* < 0.001), *ASXL1* (*p* = 0.016), *PIK3CA* (*p* = 0.03), *SMAD4* (*p* = 0.037), and *KMT2D* (*p* = 0.04) was confirmed. In addition, significant co‐occurrence of *NTRK* fusions with alterations of *NTRK1* (*p* = 0.002), *SDHC* (*p* = 0.004), *TMPRSS2‐ERG* fusion (*p* = 0.005), *CDK4* (*p* = 0.006), and *SDHD* (*p* = 0.01) were found (Table S6). In bowel cancer, *NTRK* fusion genes were detected overall in just five cases: three of 6661 (0.05%) with MSI stable (MSI‐low) status; and two of 742 (0.27%) with MSI‐high status.

In Ewing sarcoma in soft tissue, one case was identified with an *NTRK* fusion gene but an *EWSR1* fusion gene was not detected. This case illustrates the difficulty of histological diagnosis in rare cancers and suggests reclassification as *NTRK*‐rearranged spindle cell neoplasms (NTRK‐RSCNs). In addition, *NTRK* fusion genes were identified in 10 unclassifiable sarcomas or Sarcoma NOS, which also should be reclassified as *NTRK*‐RSCNs.

*NTRK* fusion gene frequency by histological type in head and neck, thyroid, and soft tissue, at the time of CGP ordering, is shown in Table S7.

## 4 DISCUSSION

In this study, the prevalence of *NTRK* gene fusion in Japanese patients with solid tumors recorded in the C‐CAT database was 0.20% and rates were higher in pediatric cases (1.69%) than in adults (0.16%). Prevalence was similar to previous analyses of large cancer populations.^7^, ^8^, ^10^ While Okamura et al. observed similar rates of *NTRK* fusions in adult (0.31%) and pediatric (0.34%) tumors,^7^ Westphalen and colleagues reported higher prevalence in pediatric patients (1.34%) compared with adults (0.28%),^10^ in agreement with the present study. In this study, other *NTRK* gene rearrangements (structural variants) were detected in 0.09% of cases, and the possibility that these may be pathogenic cannot be excluded.

In cancers with ≥100 patients, the highest prevalence of *NTRK* gene fusion was found in head and neck cancer (mostly salivary gland carcinoma) and thyroid cancer (each 1.31%), and STS (0.91%). Similar results were reported in the FoundationCORE study by Westphalen and colleagues who found the highest prevalence of *NTRK* fusion genes in adults was in salivary gland cancer, followed by STS and thyroid cancer,^10^ while Rosen and colleagues reported the highest prevalence in adult and pediatric patients in salivary gland cancers followed by thyroid carcinomas and sarcomas.^8^ However, comparisons between studies should be treated with caution as prevalence is influenced by the platform used to detect fusion genes. In this respect, both the current and FoundationCORE^10^ studies primarily used the same testing platform (FoundationOne CDx).

Although the number of pediatric patients with a detectable *NTRK* fusion gene was relatively low (*n* = 17), STS accounted for nearly half of all pediatric cases (47.06%) and CNS/brain cancer almost a quarter of cases (23.53%). In a recent large study of sarcoma, CGP identified potentially actionable kinase fusions (including *NTRK1‐3*, *ALK*, *BRAF*, *FGFR1‐4*, *RET*, and *ROS1*) in 2.6% of patients.^13^ Due to the favorable efficacy and safety of TRK inhibitors, the World Sarcoma Network (WSN) recommends that *NTRK* gene fusion testing should be incorporated into the clinical management of patients with sarcoma.^30^ More than 90% of infantile fibrosarcoma cases have an *NTRK* gene fusion^31^ and, in the present study, an *NTRK* gene fusion was found in three cases of infantile fibrosarcoma representing 15.79% of all STS *NTRK* fusion gene cases. Other main STS types were unclassified sarcoma (36.84%) and sarcoma NOS (15.79%). These unclassified sarcoma cases should be reclassified as NTRK‐RSCNs. In some cases, sarcomas are difficult to diagnose by pathological examination alone, and the identification of an *NTRK* fusion gene by CGP testing has led to a more accurate diagnosis and the potential for TRK inhibitor therapy.

A relatively low frequency of *NTRK* gene fusion was observed in this study for lung cancer (0.08%), in general agreement with large Chinese studies which reported rates of 0.09% (4/4619) in lung adenocarcinoma^32^ and 0.06% (12/21,155) in lung cancer^33^ patients. However, a systematic literature review and meta‐analysis of *NTRK* gene fusion in solid tumors estimated a higher frequency of 0.17% in non‐small cell lung cancer (NSCLC).^9^ These results may be due to Asian lung cancer patients having a relatively low frequency of *NTRK* gene fusion, or DNA‐based NGS does not detect the breakpoint within an intronic region of a *NTRK* gene fusion which may be common in lung cancer.

Based on the WSN recommendation that *NTRK* gene fusion testing should be incorporated into the clinical management of patients with sarcoma,^30^ there is a need to screen efficiently since the overall frequency of NTRK fusions is low. For screening of tumors with a known low frequency of *NTRK* fusions where NGS is not routinely performed (e.g., colon cancer), Penault‐Llorca and colleagues recommend pan‐TRK immunohistochemistry (IHC) and, if positive, NGS.^22^ Currently, there is no clear clinical evidence for testing algorithms incorporating pan‐TRK IHC for low frequency tumors. Our tumor‐agnostic CGP study yielded results that were similar to those in Europe and the United States. Based on this fact, it is necessary to consider the availability and use of CGP and IHC for diagnosis to verify the optimal testing strategy in Japan.

This study identified multiple genes which showed statistically significant mutual exclusivity or co‐occurrence with *NTRK* fusions. Four genes (*KRAS*, *APC*, *TP53*, and *PIK3CA*), which demonstrated mutual exclusivity with *NTRK* fusions, were also identified by Westphalen and colleagues in the FoundationCORE study.^10^ Only one gene (*CDK4*) was identified which significantly co‐occurred with *NTRK* fusions in both studies.

In this study, *NTRK* gene fusions were identified in 21 different solid tumor types involving 38 different partner genes of which, 22 (57.9%) were previously unreported. Recent studies of patients with TRK fusion cancer have also identified a high proportion (66%^10^ and 72%^12^) of previously unreported partner genes in *NTRK* gene fusions.

*NTRK* gene fusion products are proposed to lead to constitutive kinase activation following dimerization which is mediated by upstream gene partner oligomerization domains, such as the coiled‐coil domain.^3^, ^34^ In this study, among 22 previously unreported fusion partners, four cases contained a coiled‐coil domain. The majority of cases (81.8%) are assumed to have an alternative mechanism of dimerization or an unknown mechanism of fusion kinase activation.

The current study analyzed C‐CAT data from three different genomic platforms which increased the likelihood of detection. For example, the *ETV6::NTRK3* fusion gene (breakpoints located in introns 4 and 13, respectively) was identified in thyroid cancer by the OncoGuide NCC Oncopanel System which was not covered by FoundationOne CDx. While it is important to continue CGP of solid tumors to identify more *NTRK* fusion gene partners, the choice of CGP platform can influence the detection rate due to differences in genomic coverage of probes and reduced sensitivity to detect fusions from circulating tumor DNA. Considering the complexity of *NTRK* gene fusions, RNA‐based NGS may be the most suitable method to increase sensitivity and specificity.

*ETV6::NTRK3* was the only *NTRK* fusion gene detected in head and neck cancer and is known to be common in salivary gland carcinoma.^35^ A high prevalence of *ETV6::NTRK3* in salivary gland carcinoma in the present study is probable as most head and neck cancers were identified as salivary gland carcinoma (72.7%) and the rest were unclassified. Many other solid tumors with detectable *NTRK* fusion genes were unspecified or *NTRK*‐RSCNs.

This study showed that, across all tumor types, most *NTRK* fusion genes involved *NTRK1* (49.5%) or *NTRK3* (44.2%). Similarly, *NTRK* gene fusions involving *NTRK3* (48%) or *NTRK1* (39%) were common in patients with TRK fusion cancer prior to enrollment in clinical trials involving larotrectinib.^12^

In Ewing sarcoma of soft tissue, one case was identified with an *NTRK* fusion gene but an *EWSR1* fusion gene was not detected. This case illustrates the difficulty of histological diagnosis in rare cancers and suggests reclassification as *NTRK*‐RSCNs. In addition, *NTRK* fusion genes were identified in 10 unclassifiable sarcomas or Sarcoma NOS, which also should be re‐classified as NTRK‐RSCNs. CGP testing may provide a definitive diagnosis when the tissue diagnosis is difficult. Thus, identification of *NTRK* fusion genes enables accurate diagnosis and TRK inhibitor treatment as a therapeutic option, especially for STS.

This study has several limitations. The registered patient backgrounds may differ from the prevalence rates of different cancer types: as mentioned above, CGP testing in Japan is covered by health insurance only for advanced solid tumors in patients who have completed, or will soon complete, standard therapies.^19^ Consequently, cancer types with few available standard therapies may be overrepresented in the C‐CAT database. Criteria for *NTRK* fusion gene positivity may be variable due to the range of diagnostic testing approaches available and selection of cutoff values,^5^, ^12^ and diagnosis of some rare cancers may be problematic. The number of cases analyzed was limited for some cancer types (e.g., some pediatric cancers) and prevalence could not be estimated accurately. Although this study demonstrated potential dimerization of *NTRK* fusion gene, it cannot be assumed that all *NTRK* fusion genes, especially those with novel fusion gene partners, are pathogenic and activating fusions. However, in clinical trials of larotrectinib for patients with TRK fusion‐positive solid tumors, the overall response rate according to independent review was 75%.^14^ We can speculate that most of the identified *NTRK* fusion genes are likely to be pathogenic. It should also be noted that the FoundationCORE study period was from January 2013 to December 2019,^10^ whereas the current study period was from 1 June 2019 to 23 February 2023; therefore, although the FoundationCORE dataset included a comprehensive analysis of multiple races, but the C‐CAT database only analyses Japanese data, there is a 6‐month overlap which may possibly account for some duplication between the studies. Finally, as there are limited prognostic data in the C‐CAT database, survival data for patients with an *NTRK* gene fusion could not be determined.

In conclusion, this study found that the prevalence of *NTRK* gene fusion in Japanese patients with solid tumors was 0.2%, and rates were higher in pediatric cases (1.7%) than adults (0.2%). Identification of *NTRK* gene fusions in solid tumors with CGP testing enables an accurate diagnosis and TRK inhibitor treatment as a potential therapeutic option.

## AUTHOR CONTRIBUTIONS

**Eiji Nakata:** Conceptualization (equal); supervision (equal); writing – original draft (equal); writing – review and editing (equal). **Tatsunori Osone:** Conceptualization (equal); supervision (equal); writing – original draft (equal); writing – review and editing (equal). **Toru Ogawa:** Conceptualization (equal); data curation (equal); formal analysis (equal); project administration (equal); supervision (equal); writing – original draft (equal); writing – review and editing (equal). **Tomoyuki Taguchi:** Conceptualization (equal); data curation (equal); formal analysis (equal); project administration (equal); writing – original draft (equal); writing – review and editing (equal). **Kana Hattori:** Conceptualization (equal); data curation (equal); formal analysis (equal); supervision (equal); writing – original draft (equal); writing – review and editing (equal). **Shinji Kohsaka:** Conceptualization (equal); writing – original draft (equal); writing – review and editing (equal).

## FUNDING INFORMATION

The study was supported by Bayer Yakuhin Ltd., Japan.

## CONFLICT OF INTEREST STATEMENT

Kana Hattori, Toru Ogawa, and Tomoyuki Taguchi are employees of Bayer Yakuhin, Ltd. Eiji Nakata, Tatsunori Osone, and Shinji Kohsaka declare no conflict of interest.

## ETHICS STATEMENTS

Approval of the research protocol by an institutional review board: This study was approved by the ethics committee of the Non‐Profit Organization MINS. Registry and registration no. of the study/trial: UMIN000050715, NCT05793281.

## Supporting information


Table S1.

Table S2.

Table S3.

Table S4.

Table S5.

Table S6.

Table S7.

## Data Availability

The data that support the findings of this study are available from the corresponding author upon reasonable request.
