# Supplementary material for: Prevalence of neurotrophic tropomyosin receptor kinase (NTRK) fusion gene positivity in patients with solid tumors in Japan
Source: Cancer Med. 2024 Jun 25;13(12):e7351. doi: 10.1002/cam4.7351 (PMC11199329; doi:10.1002/cam4.7351)
Supplement: Supplementary file 1 — Table S1. [file CAM4-13-e7351-s005.docx]

Supplementary Tables

**Supplementary Table 1 *NTRK* gene fusion and other *NTRK* gene alterations by gender**

|  | **n (%)** |
| --- | --- |
| **All** | 46,621 (100) |
| with *NTRK* gene fusion | 91 (0.20) |
| with other *NTRK* gene rearrangement | 40 (0.09) |
| Adults | 45,613 (100) |
| with *NTRK* gene fusion | 74 (0.16) |
| with other *NTRK* gene rearrangement | 39 (0.09) |
| Pediatric | 1,008 (100) |
| with *NTRK* gene fusion | 17 (1.69) |
| with other *NTRK* gene rearrangement | 1 (0.10) |
| **Male** | 23,157 (100) |
| with *NTRK* gene fusion | 50 (0.22) |
| with other *NTRK* gene rearrangement | 26 (0.11) |
| Adults | 22,647 (100) |
| with *NTRK* gene fusion | 40 (0.18) |
| with other *NTRK* gene rearrangement | 26 (0.11) |
| Pediatric | 510 (100) |
| with *NTRK* gene fusion | 10 (1.96) |
| with other *NTRK* gene rearrangement | 0 (0) |
| **Female** | 23,459 (100) |
| with *NTRK* gene fusion | 41 (0.17) |
| with other *NTRK* gene rearrangement | 14 (0.06) |
| Adult | 22,962 (100) |
| with *NTRK* gene fusion | 34 (0.15) |
| with other *NTRK* gene rearrangement | 13 (0.06) |

| Pediatric | 497 (100) |
| --- | --- |
| with *NTRK* gene fusion | 7 (1.41) |
| with other *NTRK* gene rearrangement | 1 (0.20) |

Other *NTRK* gene alterations include duplication, long deletion, inversion, truncation, splice variants and other chromosomal-level rearrangements; and unconfirmed *NTRK* fusion gene partners
