# Supplementary material for: Prevalence of neurotrophic tropomyosin receptor kinase (NTRK) fusion gene positivity in patients with solid tumors in Japan
Source: Cancer Med. 2024 Jun 25;13(12):e7351. doi: 10.1002/cam4.7351 (PMC11199329; doi:10.1002/cam4.7351)
Supplement: Supplementary file 2 — Table S2. [file CAM4-13-e7351-s007.docx]

Supplementary Table 2 Proportion of *NTRK* gene fusions and other *NTRK*

rearrangements by gene profiling platform

| **Gene profiling platform** | **Patients** | **n (%)** |
| --- | --- | --- |
| FoundationOne^®^ CDx | All | 35,099 (100) |
|  | with *NTRK* gene fusion | 77 (0.22) |
|  | with other *NTRK* gene rearrangement | 26 (0.07) |
|  | Adult | 34,268 (100) |
|  | with *NTRK* gene fusion | 61 (0.18) |
|  | with other *NTRK* gene rearrangement | 25 (0.07) |
|  | Pediatric | 831 (100) |
|  | with *NTRK* gene fusion | 16 (1.93) |
|  | with other *NTRK* gene rearrangement | 1 (0.12) |
| FoundationOne^®^ Liquid CDx | All | 6,117 (100) |
|  | with *NTRK* gene fusion | 6 (0.10) |
|  | with other *NTRK* gene rearrangement | 14 (0.23) |
|  | Adult | 6,075 (100) |
|  | with *NTRK* gene fusion | 6 (0.10) |
|  | with other *NTRK* gene rearrangement | 14 (0.23) |
|  | Pediatric | 42 (100) |
|  | with *NTRK* gene fusion | 0 (0.00) |
|  | with other *NTRK* gene rearrangement | 0 (0) |
| OncoGuide^TM^ NCC Oncopanel System | All | 5,405 (100) |
|  | with *NTRK* gene fusion | 8 (0.15) |
|  | with other *NTRK* gene rearrangement | 0 (0) |
|  | Adult | 5,270 (100) |
|  | with *NTRK* gene fusion | 7 (0.13) |

|  | with other *NTRK* gene rearrangement | 0 (0) |
| --- | --- | --- |
|  | Pediatric | 135 (100) |
|  | with *NTRK* gene fusion | 1 (0.74) |
|  | with other *NTRK* gene rearrangement | 0 (0) |
