# Supplementary material for: Prevalence of neurotrophic tropomyosin receptor kinase (NTRK) fusion gene positivity in patients with solid tumors in Japan
Source: Cancer Med. 2024 Jun 25;13(12):e7351. doi: 10.1002/cam4.7351 (PMC11199329; doi:10.1002/cam4.7351)
Supplement: Supplementary file 3 — Table S3. [file CAM4-13-e7351-s002.docx]

# Supplementary Table 3 *NTRK* fusion gene patient list

| Cancer type | Diagnosis name | Age | OncoGuideTM  NCC Oncopanel  system | TMB | MSI | Alteration type | Marker | Mutation context | Mutation allele frequency | Chromosome | Position |
| --- | --- | --- | --- | --- | --- | --- | --- | --- | --- | --- | --- |
| Breast | Juvenile Secretory Carcinoma of the Breast | 51 | FoundationOne  CDx DX1 | 0Muts/Mb() | stable | rearrangement | ETV6-NTRK3 | fusion |  | 15:12 | 87943237-87943237:11879138-  11879138 |
|  |  |  |  |  |  | small_scale_variant | KMT2A | P1838S | 0.49 | 11 | 118495857 |
|  |  |  |  |  |  | small_scale_variant | PDGFRA | D1033V | 0.5 | 4 | 54290530 |
|  |  |  |  |  |  | small_scale_variant | PTCH1 | R1303C | 0.5 | 9 | 95447349 |
|  |  |  |  |  |  | small_scale_variant | RET | V292M | 0.49 | 10 | 43106382 |
|  |  |  |  |  |  | small_scale_variant | RET | V292M | 0.49 | 10 | 43106382 |
| Bowel | Colon Adenocarcinoma | 72 | FoundationOne CDx DX1 | 21Muts/Mb(high) | high | rearrangement | ETV6-NTRK3 | fusion |  | 15:12 | 87951298-87951298:11884118-  11884118 |
|  |  |  |  |  |  | small_scale_variant | ALK | F241fs*8 | 0.26 | 2 | 29717641 |
|  |  |  |  |  |  | small_scale_variant | ARAF | F107del | 0.07 | X | 47564999 |
|  |  |  |  |  |  | small_scale_variant | ARID1A | Q372fs*19 | 0.18 | 1 | 26697515 |
|  |  |  |  |  |  | small_scale_variant | ATM | L1194fs*1 | 0.14 | 11 | 108282713 |
|  |  |  |  |  |  | small_scale_variant | ATM | L1194fs*1 | 0.14 | 11 | 108282713 |
|  |  |  |  |  |  | small_scale_variant | ATM | L1194fs*1 | 0.14 | 11 | 108282713 |
|  |  |  |  |  |  | small_scale_variant | CASP8 | L487P | 0.31 | 2 | 201286563 |
|  |  |  |  |  |  | small_scale_variant | CBL | L272fs*1 | 0.29 | 11 | 119274898 |
|  |  |  |  |  |  | small_scale_variant | CSF3R | S155G | 0.38 | 1 | 36473786 |
|  |  |  |  |  |  | small_scale_variant | CTCF | T204fs*26 | 0.26 | 16 | 67611442 |
|  |  |  |  |  |  | small_scale_variant | CTNNB1 | W383R | 0.03 | 3 | 41233406 |
|  |  |  |  |  |  | small_scale_variant | DOT1L | D610N | 0.27 | 19 | 2214501 |
|  |  |  |  |  |  | small_scale_variant | EPHB1 | A729T | 0.25 | 3 | 135201528 |
|  |  |  |  |  |  | small_scale_variant | HDAC1 | E446fs*2 | 0.12 | 1 | 32332206 |
|  |  |  |  |  |  | small_scale_variant | HNF1A | L377fs*7 | 0.29 | 12 | 120996561 |
|  |  |  |  |  |  | small_scale_variant | KLHL6 | R250* | 0.25 | 3 | 183508220 |
|  |  |  |  |  |  | small_scale_variant | KMT2D | P2354fs*30 | 0.19 | 12 | 49040708 |
|  |  |  |  |  |  | small_scale_variant | LTK | V113M | 0.26 | 15 | 41512729 |
|  |  |  |  |  |  | small_scale_variant | MAP3K1 | A241T | 0.48 | 5 | 56859802 |
|  |  |  |  |  |  | small_scale_variant | MCL1 | P101L | 0.28 | 1 | 150579229 |
|  |  |  |  |  |  | small_scale_variant | MET | D543N | 0.26 | 7 | 116740951 |
|  |  |  |  |  |  | small_scale_variant | MSH3 | K383fs*32 | 0.31 | 5 | 80675102 |
|  |  |  |  |  |  | small_scale_variant | MSH3 | N739fs*29 | 0.29 | 5 | 80768966 |
|  |  |  |  |  |  | small_scale_variant | NOTCH2 | V1321L | 0.26 | 1 | 119926543 |
|  |  |  |  |  |  | small_scale_variant | NOTCH3 | L1590I | 0.25 | 19 | 15170794 |

|  |  |  |  |  |  | small_scale_variant | PBRM1 | N258fs*26 | 0.3 | 3 | 52648383 |
| --- | --- | --- | --- | --- | --- | --- | --- | --- | --- | --- | --- |
|  |  |  |  |  |  | small_scale_variant | PDGFRA | D1033V | 0.48 | 4 | 54290530 |
|  |  |  |  |  |  | small_scale_variant | PIK3C2B | C395F | 0.27 | 1 | 204464455 |
|  |  |  |  |  |  | small_scale_variant | PIK3R1 | I30V | 0.62 | 5 | 68290816 |
|  |  |  |  |  |  | small_scale_variant | POLE | Q2035H | 0.28 | 12 | 132632695 |
|  |  |  |  |  |  | small_scale_variant | PTPRO | A86G | 0.17 | 12 | 15484155 |
|  |  |  |  |  |  | small_scale_variant | RNF43 | G659fs*41 | 0.65 | 17 | 58357799 |
|  |  |  |  |  |  | small_scale_variant | RNF43 | R117fs*41 | 0.34 | 17 | 58370936 |
|  |  |  |  |  |  | small_scale_variant | ROS1 | L590P | 0.54 | 6 | 117388025 |
|  |  |  |  |  |  | small_scale_variant | TGFBR2 | K128fs*3 | 0.03 | 3 | 30650387 |
|  |  |  |  |  |  | small_scale_variant | TGFBR2 | K128fs*35 | 0.23 | 3 | 30650388 |
|  |  |  |  |  |  | small_scale_variant | TGFBR2 | P129fs*3 | 0.18 | 3 | 30650389 |
| Soft Tissue | Infantile Fibrosarcoma | 0 | FoundationOne CDx DX1 | 1Muts/Mb() | stable | rearrangement | ETV6-NTRK3 | fusion |  | 15:12 | 87967701-87967701:11883680-  11883680 |
|  |  |  |  |  |  | small_scale_variant | FANCG | S315N | 0.51 | 9 | 35076564 |
|  |  |  |  |  |  | small_scale_variant | MTOR | A432V | 0.48 | 1 | 11243231 |
|  |  |  |  |  |  | small_scale_variant | NOTCH1 | G889S | 0.51 | 9 | 136510728 |
|  |  |  |  |  |  | small_scale_variant | RET | A641T | 0.5 | 10 | 43114521 |
| Head and Neck | Mammary Analogue Secretory Carcinoma of Salivary Gland Origin | 65 | FoundationOne CDx DX1 | 3Muts/Mb() | stable | copy_number_alter ation | CDKN2A | loss |  | 9 | 21853213-21998003 |
|  |  |  |  |  |  | copy_number_alter  ation | CDKN2B | loss |  | 9 | 21998749-22101833 |
|  |  |  |  |  |  | copy_number_alter  ation | MTAP | loss |  | 9 | 21802700-21862059 |
|  |  |  |  |  |  | rearrangement | ETV6-NTRK3 | fusion |  | 15:12 | 87984781-87984781:11879891-  11879891 |
|  |  |  |  |  |  | small_scale_variant | APC | R2673G | 0.51 | 5 | 112843611 |
|  |  |  |  |  |  | small_scale_variant | DAXX | W622G | 0.49 | 6 | 33319456 |
|  |  |  |  |  |  | small_scale_variant | DDR1 | R188T | 0.48 | 6 | 30891118 |
|  |  |  |  |  |  | small_scale_variant | MST1R | V670G | 0.5 | 3 | 49897557 |
|  |  |  |  |  |  | small_scale_variant | NOTCH3 | R2237G | 0.49 | 19 | 15160919 |
|  |  |  |  |  |  | small_scale_variant | PALB2 | S652N | 0.4 | 16 | 23630199 |
|  |  |  |  |  |  | small_scale_variant | RAD51D | V66M | 0.5 | 17 | 35118568 |
|  |  |  |  |  |  | small_scale_variant | ROS1 | R2269L | 0.22 | 6 | 117288730 |
|  |  |  |  |  |  | small_scale_variant | SMARCA4 | P222L | 0.47 | 19 | 10986498 |
|  |  |  |  |  |  | small_scale_variant | SPEN | I3084V | 0.19 | 1 | 15935490 |
| CNS/Brain | High-Grade Glioma, NOS | 0 | FoundationOne CDx DX2 | 0Muts/Mb() | stable | rearrangement | TPM3-NTRK1 | fusion |  | 1:01 | 156875290-  156875602:154166747-154167025 |
|  |  |  |  |  |  | small_scale_variant | ASXL1 | G669S | 0.5054 | 20 | 32434717 |
|  |  |  |  |  |  | small_scale_variant | DIS3 | A656V | 0.4911 | 13 | 72763521 |
|  |  |  |  |  |  | small_scale_variant | EZH2 | R213H | 0.5183 | 7 | 148827254 |

|  |  |  |  |  |  | small_scale_variant | KMT2A | A53V | 0.5802 | 11 | 118436670 |
| --- | --- | --- | --- | --- | --- | --- | --- | --- | --- | --- | --- |
| Soft Tissue | Angiosarcoma | 66 | FoundationOne CDx DX2 | 2Muts/Mb() | stable | copy_number_alter ation | FLCN | loss |  | 17 | 17213654-17228137 |
|  |  |  |  |  |  | copy_number_alter  ation | GID4 | loss |  | 17 | 17992767-18111749 |
|  |  |  |  |  |  | copy_number_alter  ation | MYC | amplification |  | 8 | 127694344-127740958 |
|  |  |  |  |  |  | copy_number_alter  ation | NOTCH1 | loss |  | 9 | 136496070-136531172 |
|  |  |  |  |  |  | copy_number_alter  ation | POLE | loss |  | 12 | 132624688-132687349 |
|  |  |  |  |  |  | copy_number_alter  ation | TP53 | loss |  | 17 | 7597317-7686123 |
|  |  |  |  |  |  | rearrangement | MYC | rearrangements |  | 8:08 | 127738394-  127738814:127277453-127277773 |
|  |  |  |  |  |  | rearrangement | PEAR1-NTRK1 | fusion |  | 1:01 | 156874221-  156874591:156910860-156911164 |
|  |  |  |  |  |  | rearrangement | RPTOR | rearrangements |  | 17:17 | 80961281-80961580:80952741-  80952864 |
|  |  |  |  |  |  | rearrangement | TP53 | rearrangements |  | 17:17 | 7686075-7686254:7649467-  7649751 |
|  |  |  |  |  |  | small_scale_variant | CASP8 | R430* | 0.4444 | 2 | 201285250 |
|  |  |  |  |  |  | small_scale_variant | ERBB2 | P378L | 0.3493 | 17 | 39712433 |
|  |  |  |  |  |  | small_scale_variant | NKX2-1 | G322S | 0.3392 | 14 | 36517430 |
|  |  |  |  |  |  | small_scale_variant | SPEN | R231K | 0.4964 | 1 | 15876489 |
|  |  |  |  |  |  | small_scale_variant | TBX3 | A675_L677del | 0.5842 | 12 | 114672042 |
|  |  |  |  |  |  | small_scale_variant | VEGFA | S186Y | 0.4933 | 6 | 43784609 |
|  |  |  |  |  |  | small_scale_variant | VHL | M1I | 0.6354 | 3 | 10141850 |
| Thyroid | Papillary Thyroid Cancer | 63 | FoundationOne CDx DX2 | 1Muts/Mb() | stable | rearrangement | TPM3-NTRK1 | fusion |  | 1:01 | 156874381-  156874381:154162366-154162366 |
|  |  |  |  |  |  | small_scale_variant | DIS3 | P701S | 0.4821 | 13 | 72762074 |
|  |  |  |  |  |  | small_scale_variant | GRM3 | F828L | 0.4965 | 7 | 86850462 |
|  |  |  |  |  |  | small_scale_variant | KMT2A | P3837L | 0.5027 | 11 | 118521293 |
|  |  |  |  |  |  | small_scale_variant | LTK | G212_G213insGG G | 0.5165 | 15 | 41511835 |
|  |  |  |  |  |  | small_scale_variant | NOTCH2 | N1102S | 0.4748 | 1 | 119937889 |
|  |  |  |  |  |  | small_scale_variant | RAD51C | M136L | 0.4337 | 17 | 58696694 |
|  |  |  |  |  |  | small_scale_variant | SPEN | M1588V | 0.1639 | 1 | 15931002 |
|  |  |  |  |  |  | small_scale_variant | TERT | c.-79-45C>T | 0.3901 | 5 | 1295113 |
|  |  |  |  |  |  | small_scale_variant | TSC1 | T899S | 0.43 | 9 | 132897540 |
| Thyroid | Papillary Thyroid Cancer | 52 | OncoGuideTM NCC Oncopanel system | 0.00Muts/Mb() | 1.97%(sta ble) | rearrangement | ETV6-NTRK3 | fusion | 0.2249 | 12:15 | 11857625-11857625:88043982-  88043987 |
|  |  |  |  |  |  | rearrangement | NTRK3-ETV6 | fusion | 0.2208 | 15:12 | 88043987-88043990:11857636-  11857639 |
|  |  |  |  |  |  | small_scale_variant | MAP3K1 | V889L | 0.471 (317/673) | 5 | 56881865 |
| Biliary Tract | Intrahepatic Cholangiocarcinoma | 56 | FoundationOne Liquid CDx AB1 | 3Muts/Mb() | high not detected | rearrangement | NTRK1-BPI | fusion |  | 1:20 | 156875515-156875515:38324204-  38324204 |

|  |  |  |  |  |  | small_scale_variant | ATRX | D774fs*29 | 0.0074 | X | 77682939 |
| --- | --- | --- | --- | --- | --- | --- | --- | --- | --- | --- | --- |
|  |  |  |  |  |  | small_scale_variant | BRIP1 | G481D | 0.4792 | 17 | 61793628 |
|  |  |  |  |  |  | small_scale_variant | CBL | R593fs*13 | 0.0047 | 11 | 119285402 |
|  |  |  |  |  |  | small_scale_variant | DAXX | L500P | 0.4922 | 6 | 33319821 |
|  |  |  |  |  |  | small_scale_variant | DNMT3A | G685R | 0.0026 | 2 | 25241591 |
|  |  |  |  |  |  | small_scale_variant | DNMT3A | I695S | 0.0082 | 2 | 25240729 |
|  |  |  |  |  |  | small_scale_variant | DNMT3A | Q678* | 0.0099 | 2 | 25241612 |
|  |  |  |  |  |  | small_scale_variant | EPHB1 | T27M | 0.5375 | 3 | 134925837 |
|  |  |  |  |  |  | small_scale_variant | LTK | G212_G213insGG G | 0.9989 | 15 | 41511835 |
|  |  |  |  |  |  | small_scale_variant | NTRK2 | I518V | 0.4695 | 9 | 84867350 |
|  |  |  |  |  |  | small_scale_variant | PIK3C2B | P285S | 0.4961 | 1 | 204468950 |
|  |  |  |  |  |  | small_scale_variant | PTCH1 | G29V | 0.4946 | 9 | 95516735 |
|  |  |  |  |  |  | small_scale_variant | SDHD | V111I | 0.5075 | 11 | 112094821 |
|  |  |  |  |  |  | small_scale_variant | SMO | G277S | 0.483 | 7 | 129205691 |
|  |  |  |  |  |  | small_scale_variant | STK11 | F354L | 0.4921 | 19 | 1223126 |
| Uterus | Uterine Serous Carcinoma/Uterine Papillary Serous Carcinoma | 74 | FoundationOne CDx DX2 | 2Muts/Mb() | stable | rearrangement | DDR1 | rearrangements |  | 6:06 | 30897966-30898075:30858779-  30858939 |
|  |  |  |  |  |  | rearrangement | ESR1 | rearrangements |  | 6:07 | 152098959-152099087:61083436-  61083607 |
|  |  |  |  |  |  | rearrangement | NTRK1 | rearrangements |  | 1:01 | 156874778-  156874957:156634689-156634870 |
|  |  |  |  |  |  | rearrangement | PRCC-NTRK1 | fusion |  | 1:01 | 156874429-  156874647:156799097-156799271 |
|  |  |  |  |  |  | small_scale_variant | CRKL | V279M | 0.0522 | 22 | 20949768 |
|  |  |  |  |  |  | small_scale_variant | EPHB4 | A88V | 0.4851 | 7 | 100823792 |
|  |  |  |  |  |  | small_scale_variant | FLT1 | N562K | 0.483 | 13 | 28390079 |
|  |  |  |  |  |  | small_scale_variant | LTK | G212_G213insGG G | 0.381 | 15 | 41511835 |
|  |  |  |  |  |  | small_scale_variant | NF2 | E394K | 0.0792 | 22 | 29673326 |
|  |  |  |  |  |  | small_scale_variant | NOTCH3 | R1175W | 0.4823 | 19 | 15179220 |
|  |  |  |  |  |  | small_scale_variant | PIK3C2G | P105L | 0.4806 | 12 | 18282395 |
|  |  |  |  |  |  | small_scale_variant | PIK3CA | K111E | 0.1144 | 3 | 179199156 |
|  |  |  |  |  |  | small_scale_variant | RARA | D433E | 0.3867 | 17 | 40356136 |
|  |  |  |  |  |  | small_scale_variant | SPEN | R3011Q | 0.4831 | 1 | 15935272 |
|  |  |  |  |  |  | small_scale_variant | TP53 | C238Y | 0.1823 | 17 | 7674250 |
| Soft Tissue | Sarcoma, NOS | 31 | FoundationOne CDx DX2 | 0Muts/Mb() | stable | copy_number_alter ation | CDKN2A | loss |  | 9 | 21853213-21998003 |
|  |  |  |  |  |  | copy_number_alter  ation | CDKN2B | loss |  | 9 | 21998749-22101833 |
|  |  |  |  |  |  | copy_number_alter  ation | MTAP | loss |  | 9 | 21802700-21862059 |
|  |  |  |  |  |  | rearrangement | FAT3-CBL | fusion |  | 11:11 | 119278645-119278845:92598962-  92599299 |

|  |  |  |  |  |  | rearrangement | LMNA-NTRK1 | fusion |  | 1:01 | 156874388-  156874712:156134787-156135260 |
| --- | --- | --- | --- | --- | --- | --- | --- | --- | --- | --- | --- |
|  |  |  |  |  |  | rearrangement | PTCH1 | truncation |  | 9:09 | 95480029-95480233:87326891-  87327218 |
|  |  |  |  |  |  | small_scale_variant | BRCA1 | Q1459R | 0.4983 | 17 | 43079381 |
|  |  |  |  |  |  | small_scale_variant | MAP2K2 | V400M | 0.4959 | 19 | 4090603 |
|  |  |  |  |  |  | small_scale_variant | MET | V1070M | 0.4755 | 7 | 116775060 |
|  |  |  |  |  |  | small_scale_variant | MUTYH | c.892-2A>G | 0.4874 | 1 | 45332088 |
|  |  |  |  |  |  | small_scale_variant | NBN | I171V | 0.4919 | 8 | 89978293 |
|  |  |  |  |  |  | small_scale_variant | NTRK1 | G89S | 0.3782 | 1 | 156864406 |
|  |  |  |  |  |  | small_scale_variant | TSC1 | T417I | 0.4882 | 9 | 132910584 |
| Bone | Osteosarcoma | 13 | FoundationOne CDx DX2 | 6Muts/Mb() | stable | copy_number_alter ation | ALOX12B | amplification |  | 17 | 8072770-8087442 |
|  |  |  |  |  |  | copy_number_alter  ation | ATM | amplification |  | 11 | 108051655-108543256 |
|  |  |  |  |  |  | copy_number_alter  ation | AURKB | amplification |  | 17 | 8156471-8257961 |
|  |  |  |  |  |  | copy_number_alter  ation | CBL | amplification |  | 11 | 119206417-119299781 |
|  |  |  |  |  |  | copy_number_alter  ation | CCND1 | amplification |  | 11 | 69597245-69687252 |
|  |  |  |  |  |  | copy_number_alter  ation | CCND1 | amplification |  | 11 | 69597245-69687252 |
|  |  |  |  |  |  | copy_number_alter  ation | CDK4 | amplification |  | 12 | 57700149-57794361 |
|  |  |  |  |  |  | copy_number_alter  ation | CHEK1 | amplification |  | 11 | 125626736-125655336 |
|  |  |  |  |  |  | copy_number_alter  ation | ERCC4 | amplification |  | 16 | 13920165-13948347 |
|  |  |  |  |  |  | copy_number_alter  ation | FGF19 | amplification |  | 11 | 69699261-69747018 |
|  |  |  |  |  |  | copy_number_alter  ation | FGF3 | amplification |  | 11 | 69765547-69868824 |
|  |  |  |  |  |  | copy_number_alter  ation | FGF4 | amplification |  | 11 | 69773308-69775084 |
|  |  |  |  |  |  | copy_number_alter  ation | GID4 | amplification |  | 17 | 17992767-18111749 |
|  |  |  |  |  |  | copy_number_alter  ation | KMT2A | amplification |  | 11 | 118436512-118522172 |
|  |  |  |  |  |  | copy_number_alter  ation | MRE11 | amplification |  | 11 | 94420091-94492853 |
|  |  |  |  |  |  | copy_number_alter  ation | SDHD | amplification |  | 11 | 112086868-112094970 |
|  |  |  |  |  |  | rearrangement | ALK | rearrangements |  | 2:02 | 29224511-29224874:29232394-  29232645 |
|  |  |  |  |  |  | rearrangement | ALK | rearrangements |  | 2:02 | 29224885-29225169:20878541-  20878794 |
|  |  |  |  |  |  | rearrangement | NTRK2-  CLEC16A | fusion |  | 9:16 | 84860909-84861239:11122677-  11122844 |
|  |  |  |  |  |  | small_scale_variant | ERBB4 | A1055P | 0.5731 | 2 | 211387965 |
|  |  |  |  |  |  | small_scale_variant | ERBB4 | A769S | 0.0569 | 2 | 211562085 |
|  |  |  |  |  |  | small_scale_variant | FLT3 | R741T | 0.4127 | 13 | 28024929 |
|  |  |  |  |  |  | small_scale_variant | MAP3K13 | I523V | 0.5261 | 3 | 185466887 |

|  |  |  |  |  |  | small_scale_variant | NTRK2 | G480D | 0.3738 | 9 | 84861082 |
| --- | --- | --- | --- | --- | --- | --- | --- | --- | --- | --- | --- |
|  |  |  |  |  |  | small_scale_variant | PDCD1LG2 | c.632-1G>C | 0.0651 | 9 | 5557617 |
|  |  |  |  |  |  | small_scale_variant | STK11 | F354L | 0.4111 | 19 | 1223126 |
| Soft Tissue | Ewing Sarcoma of Soft Tissue | 50 | FoundationOne CDx DX1 | 12.61Muts/Mb() | stable | copy_number_alter ation | EGFR | amplification |  | 7 | 54970060-55255535 |
|  |  |  |  |  |  | copy_number_alter  ation | GRM3 | amplification |  | 7 | 86765145-86864383 |
|  |  |  |  |  |  | copy_number_alter  ation | HGF | amplification |  | 7 | 81665128-81813236 |
|  |  |  |  |  |  | copy_number_alter  ation | IKZF1 | amplification |  | 7 | 50319016-50400627 |
|  |  |  |  |  |  | rearrangement | PHF20-NTRK1 | fusion |  | 1:20 | 156868050-156868299:35829618-  35829757 |
|  |  |  |  |  |  | small_scale_variant | AMER1 | T1100fs*9 | 0.16 (151/954) | X | 64189989 |
|  |  |  |  |  |  | small_scale_variant | AXIN1 | V683L | 0.49 (558/1133) | 16 | 293627 |
|  |  |  |  |  |  | small_scale_variant | AXL | V150A | 0.98 (406/415) | 19 | 41221919 |
|  |  |  |  |  |  | small_scale_variant | BCORL1 | T1111M | 0.66 (594/897) | X | 130016104 |
|  |  |  |  |  |  | small_scale_variant | DNMT3A | P451S | 0.63 (574/908) | 2 | 25246238 |
|  |  |  |  |  |  | small_scale_variant | FANCG | S315N | 0.32 (303/935) | 9 | 35076564 |
|  |  |  |  |  |  | small_scale_variant | KEAP1 | M499V | 0.98 (512/521) | 19 | 10489684 |
|  |  |  |  |  |  | small_scale_variant | MSH3 | A54_A59del | 0.20 (219/1107) | 5 | 80654888 |
|  |  |  |  |  |  | small_scale_variant | MSH3 | A992S | 0.51 (524/1019) | 5 | 80854290 |
|  |  |  |  |  |  | small_scale_variant | NF1 | V351fs*25 | 0.85 (298/349) | 17 | 31200583 |
|  |  |  |  |  |  | small_scale_variant | PDGFRB | M655L | 0.40 (498/1255) | 5 | 150124310 |
|  |  |  |  |  |  | small_scale_variant | PRDM1 | S336G | 0.26 (333/1263) | 6 | 106105166 |
|  |  |  |  |  |  | small_scale_variant | RB1 | S127N | 0.96 (452/472) | 13 | 48342714 |
|  |  |  |  |  |  | small_scale_variant | SRC | P434L | 0.51 (517/1020) | 20 | 37402779 |
|  |  |  |  |  |  | small_scale_variant | TIPARP | C552W | 0.50 (658/1313) | 3 | 156704813 |
|  |  |  |  |  |  | small_scale_variant | TP53 | T125K | 0.96 (386/403) | 17 | 7675995 |
| Biliary Tract | Perihilar Cholangiocarcinoma | 73 | FoundationOne CDx DX1 | 3.78Muts/Mb() | stable | rearrangement | KANK4-NTRK1 | fusion |  | 1:01 | 156875320-156875561:62271216-  62271463 |
|  |  |  |  |  |  | small_scale_variant | ARID1A | N1997fs*3 | 0.14 (119/857) | 1 | 26779887 |
|  |  |  |  |  |  | small_scale_variant | CTNNA1 | G160D | 0.55 (471/860) | 5 | 138812193 |
|  |  |  |  |  |  | small_scale_variant | EED | D279N | 0.47 (470/1000) | 11 | 86266191 |
|  |  |  |  |  |  | small_scale_variant | EPHB4 | R91H | 0.47 (311/663) | 7 | 100823783 |
|  |  |  |  |  |  | small_scale_variant | KLHL6 | V175I | 0.47 (654/1381) | 3 | 183508445 |
|  |  |  |  |  |  | small_scale_variant | NOTCH3 | R10_R13del | 0.49 (67/137) | 19 | 15200865 |
|  |  |  |  |  |  | small_scale_variant | PTCH1 | I395N | 0.13 (121/927) | 9 | 95479031 |
|  |  |  |  |  |  | small_scale_variant | RBM10 | K670R | 0.56 (512/909) | X | 47185113 |
|  |  |  |  |  |  | small_scale_variant | SMO | K575M | 0.53 (346/657) | 7 | 129211036 |
|  |  |  |  |  |  | small_scale_variant | SPEN | R275G | 0.53 (614/1162) | 1 | 15876620 |

|  |  |  |  |  |  | small_scale_variant | TBX3 | A491E | 0.53 (218/412) | 12 | 114674463 |
| --- | --- | --- | --- | --- | --- | --- | --- | --- | --- | --- | --- |
|  |  |  |  |  |  | small_scale_variant | TGFBR2 | N144S | 0.16 (172/1088) | 3 | 30650437 |
| Soft Tissue | Soft Tissue | 4 | FoundationOne CDx DX1 | 3.78Muts/Mb() | stable | rearrangement | TPM3-NTRK1 | fusion |  | 1:01 | 156875337-  156875647:154158778-154159043 |
|  |  |  |  |  |  | small_scale_variant | BRAF | Q165H | 0.50 (473/948) | 7 | 140834618 |
|  |  |  |  |  |  | small_scale_variant | HDAC1 | W312* | 0.28 (217/777) | 1 | 32330865 |
|  |  |  |  |  |  | small_scale_variant | IRS2 | I524V | 0.50 (285/575) | 13 | 109784484 |
|  |  |  |  |  |  | small_scale_variant | KMT2A | S3338C | 0.51 (517/1012) | 11 | 118505914 |
|  |  |  |  |  |  | small_scale_variant | NOTCH1 | P2411dup | 0.48 (254/526) | 9 | 136496506 |
|  |  |  |  |  |  | small_scale_variant | NOTCH1 | Q1134R | 0.47 (340/730) | 9 | 136508064 |
|  |  |  |  |  |  | small_scale_variant | STK11 | F354L | 0.48 (358/741) | 19 | 1223126 |
|  |  |  |  |  |  | small_scale_variant | TSC2 | V1618I | 0.45 (272/601) | 16 | 2086734 |
| CNS/Brain |  | 15 | FoundationOne CDx DX1 | 2.52Muts/Mb() | stable | rearrangement | ARHGEF2- NTRK1 | fusion |  | 1:01 | 156873925-  156874102:155948694-155948921 |
|  |  |  |  |  |  | small_scale_variant | ESR1 | R555H | 0.60 (373/618) | 6 | 152098842 |
|  |  |  |  |  |  | small_scale_variant | JAK1 | N226S | 0.20 (108/553) | 1 | 64867179 |
|  |  |  |  |  |  | small_scale_variant | KEL | R292Q | 0.51 (299/590) | 7 | 142954233 |
|  |  |  |  |  |  | small_scale_variant | MAP3K13 | I523V | 0.50 (378/755) | 3 | 185466887 |
|  |  |  |  |  |  | small_scale_variant | TP53 | P80L | 0.02 (12/556) | 17 | 7676130 |
|  |  |  |  |  |  | small_scale_variant | TSC1 | T417I | 0.56 (588/1042) | 9 | 132910584 |
| CNS/Brain |  | 5 | FoundationOne CDx DX1 | 0.0Muts/Mb() | stable | copy_number_alter ation | BTG2 | amplification |  | 1 | 203305606-203307438 |
|  |  |  |  |  |  | copy_number_alter  ation | H3F3A | amplification |  | 1 | 226064350-226071479 |
|  |  |  |  |  |  | copy_number_alter  ation | MAP3K1 | amplification |  | 5 | 56815573-56886063 |
|  |  |  |  |  |  | copy_number_alter  ation | PIK3C2B | amplification |  | 1 | 204424851-204469802 |
|  |  |  |  |  |  | copy_number_alter  ation | QKI | amplification |  | 6 | 163415193-163570766 |
|  |  |  |  |  |  | rearrangement | ARHGEF11-  NTRK1 | fusion |  | 1:01 | 156875287-  156875662:156936166-156936481 |
|  |  |  |  |  |  | rearrangement | CRB1-NTRK1 | fusion |  | 1:01 | 156874764-  156874924:197360473-197360575 |
|  |  |  |  |  |  | small_scale_variant | BCL6 | R160H | 0.44 (266/605) | 3 | 187729926 |
|  |  |  |  |  |  | small_scale_variant | BRIP1 | H478R | 0.63 (339/534) | 17 | 61793637 |
|  |  |  |  |  |  | small_scale_variant | GATA3 | G242R | 0.52 (200/382) | 10 | 8058787 |
|  |  |  |  |  |  | small_scale_variant | INPP4B | A631S | 0.48 (280/578) | 4 | 142124590 |
| Head and Neck | Head and Neck | 83 | FoundationOne CDx DX1 | 0.0Muts/Mb() | stable | copy_number_alter ation | CDKN2A | loss |  | 9 | 21968170-21974827 |
|  |  |  |  |  |  | rearrangement | ETV6-NTRK3 | fusion |  | 15:12 | 88003226-88003736:11879372-  11879983 |
|  |  |  |  |  |  | small_scale_variant | AURKB | R44H | 0.50 (671/1348) | 17 | 8207758 |
|  |  |  |  |  |  | small_scale_variant | CARD11 | A687V | 0.50 (602/1201) | 7 | 2923214 |
|  |  |  |  |  |  | small_scale_variant | CCND1 | E280del | 0.37 (335/904) | 11 | 69651232 |

|  |  |  |  |  |  | small_scale_variant | KMT2D | I5232V | 0.23 (257/1100) | 12 | 49026272 |
| --- | --- | --- | --- | --- | --- | --- | --- | --- | --- | --- | --- |
|  |  |  |  |  |  | small_scale_variant | MSH3 | A54_A59del | 0.18 (165/913) | 5 | 80654888 |
|  |  |  |  |  |  | small_scale_variant | PALB2 | E837K | 0.44 (584/1338) | 16 | 23629645 |
|  |  |  |  |  |  | small_scale_variant | SPEN | T819I | 0.50 (710/1416) | 1 | 15928696 |
|  |  |  |  |  |  | small_scale_variant | TERT | c.-79-45C>T | 0.24 (69/288) | 5 | 1295113 |
|  |  |  |  |  |  | small_scale_variant | TSC1 | P1143L | 0.47 (586/1236) | 9 | 132896302 |
|  |  |  |  |  |  | small_scale_variant | XRCC2 | V39M | 0.48 (525/1085) | 7 | 152660707 |
|  |  |  |  |  |  | small_scale_variant | ZNF703 | A511_A513del | 0.18 (158/891) | 8 | 37698431 |
| Head and Neck | Mammary Analogue Secretory Carcinoma of Salivary Gland Origin | 14 | FoundationOne CDx DX1 | 0.0Muts/Mb() | cannot be determin ed | rearrangement | ETV6-NTRK3 | fusion |  | 15:12 | 87965441-87965441:11878612-  11878612 |
|  |  |  |  |  |  | small_scale_variant | ALK | M770I | 0.52 (251/487) | 2 | 29239725 |
|  |  |  |  |  |  | small_scale_variant | BCOR | R1665W | 0.54 (381/705) | X | 40052282 |
|  |  |  |  |  |  | small_scale_variant | EGFR | I646S | 0.51 (299/592) | 7 | 55173000 |
|  |  |  |  |  |  | small_scale_variant | FGF4 | Q97R | 0.49 (126/257) | 11 | 69774795 |
|  |  |  |  |  |  | small_scale_variant | GATA3 | A396T | 0.48 (315/651) | 10 | 8073874 |
|  |  |  |  |  |  | small_scale_variant | TSC2 | S1433L | 0.47 (271/578) | 16 | 2084520 |
| Ovary/Fallopian Tube | Ovarian Cancer, Other | 63 | FoundationOne CDx DX1 | 0Muts/Mb() | stable | copy_number_alter ation | CDK4 | amplification |  | 12 | 57700149-57794361 |
|  |  |  |  |  |  | copy_number_alter  ation | CDKN2A | loss |  | 9 | 21853213-21998003 |
|  |  |  |  |  |  | copy_number_alter  ation | CDKN2B | loss |  | 9 | 21998749-22101833 |
|  |  |  |  |  |  | copy_number_alter  ation | LYN | amplification |  | 8 | 55941859-56010110 |
|  |  |  |  |  |  | copy_number_alter  ation | MDM2 | amplification |  | 12 | 68760216-68883425 |
|  |  |  |  |  |  | copy_number_alter  ation | MTAP | loss |  | 9 | 21802700-21862059 |
|  |  |  |  |  |  | rearrangement | NTRK3-CNTN1 | fusion |  | 15:12 | 88183342-88183607:40886410-  40886485 |
|  |  |  |  |  |  | rearrangement | NTRK3-IGF1R | fusion |  | 15:15 | 88183341-88183652:98919435-  98919520 |
|  |  |  |  |  |  | small_scale_variant | AXL | R48Q | 0.49 | 19 | 41220693 |
|  |  |  |  |  |  | small_scale_variant | BCOR | V379fs*62 | 0.27 | X | 40074206 |
|  |  |  |  |  |  | small_scale_variant | CDK12 | C723F | 0.49 | 17 | 39492810 |
|  |  |  |  |  |  | small_scale_variant | ERBB3 | G40S | 0.02 | 12 | 56083786 |
|  |  |  |  |  |  | small_scale_variant | GATA6 | S184N | 0.52 | 18 | 22171695 |
|  |  |  |  |  |  | small_scale_variant | NOTCH3 | A1450T | 0.71 | 19 | 15177580 |
|  |  |  |  |  |  | small_scale_variant | SPEN | R3011Q | 0.48 | 1 | 15935272 |
|  |  |  |  |  |  | small_scale_variant | TP53 | V216M | 0.34 | 17 | 7674885 |
|  |  |  |  |  |  | small_scale_variant | TP53 | V216M | 0.34 | 17 | 7674885 |
|  |  |  |  |  |  | small_scale_variant | TP53 | V216M | 0.34 | 17 | 7674885 |
|  |  |  |  |  |  | small_scale_variant | TP53 | V216M | 0.34 | 17 | 7674885 |

| Pancreas | Pancreatic Adenocarcinoma | 78 | FoundationOne  CDx DX1 | 5Muts/Mb() | stable | copy_number_alter  ation | CDKN2A | loss |  | 9 | 21853213-21998003 |
| --- | --- | --- | --- | --- | --- | --- | --- | --- | --- | --- | --- |
|  |  |  |  |  |  | copy_number_alter  ation | CDKN2B | loss |  | 9 | 21998749-22101833 |
|  |  |  |  |  |  | copy_number_alter  ation | MTAP | loss |  | 9 | 21802700-21862059 |
|  |  |  |  |  |  | copy_number_alter  ation | SMAD4 | loss |  | 18 | 50899938-51165425 |
|  |  |  |  |  |  | rearrangement | GP2-NTRK1 | fusion |  | 1:16 | 156873146-156873673:20324513-  20324875 |
|  |  |  |  |  |  | small_scale_variant | ALK | E953G | 0.21 | 2 | 29227630 |
|  |  |  |  |  |  | small_scale_variant | AMER1 | R21C | 0.49 | X | 64193226 |
|  |  |  |  |  |  | small_scale_variant | APC | K1734R | 0.51 | 5 | 112840795 |
|  |  |  |  |  |  | small_scale_variant | CARD11 | R608H | 0.48 | 7 | 2924350 |
|  |  |  |  |  |  | small_scale_variant | CDKN2C | H108Y | 0.2 | 1 | 50974085 |
|  |  |  |  |  |  | small_scale_variant | DDR2 | R307C | 0.39 | 1 | 162761274 |
|  |  |  |  |  |  | small_scale_variant | FANCA | S119C | 0.48 | 16 | 89810999 |
|  |  |  |  |  |  | small_scale_variant | FLT3 | P336L | 0.49 | 13 | 28049413 |
|  |  |  |  |  |  | small_scale_variant | MTOR | A1134V | 0.46 | 1 | 11212472 |
|  |  |  |  |  |  | small_scale_variant | NSD2 | K361Q | 0.49 | 4 | 1918294 |
|  |  |  |  |  |  | small_scale_variant | TSC2 | S1386N | 0.51 | 16 | 2084379 |
| Soft Tissue | Angiomatoid Fibrous Histiocytoma | 54 | FoundationOne CDx DX1 | 2.52Muts/Mb() | stable | rearrangement | ETV6-NTRK3 | fusion |  | 15:12 | 87959934-87959934:11880805-  11880805 |
|  |  |  |  |  |  | small_scale_variant | FGF4 | A132T | 0.52 (441/856) | 11 | 69774074 |
|  |  |  |  |  |  | small_scale_variant | KMT2A | F1445C | 0.11 (117/1046) | 11 | 118488615 |
|  |  |  |  |  |  | small_scale_variant | MST1R | R1389Q | 0.13 (123/928) | 3 | 49887344 |
|  |  |  |  |  |  | small_scale_variant | NOTCH1 | D1185N | 0.45 (352/790) | 9 | 136507395 |
|  |  |  |  |  |  | small_scale_variant | PTCH1 | E44G | 0.44 (541/1231) | 9 | 95516690 |
|  |  |  |  |  |  | small_scale_variant | SETD2 | S2382fs*29 | 0.13 (155/1224) | 3 | 47042655 |
|  |  |  |  |  |  | small_scale_variant | SPEN | A2179T | 0.49 (574/1175) | 1 | 15932775 |
|  |  |  |  |  |  | small_scale_variant | TYRO3 | P431S | 0.50 (509/1016) | 15 | 41570065 |
| Head and Neck | Mammary Analogue Secretory Carcinoma of Salivary Gland Origin | 42 | FoundationOne CDx DX1 | 0.0Muts/Mb() | stable | rearrangement | ETV6-NTRK3 | fusion |  | 15:12 | 87963867-87963867:11882085-  11882085 |
|  |  |  |  |  |  | small_scale_variant | FGFR4 | I197T | 1.00 (1127/1128) | 5 | 177091091 |
|  |  |  |  |  |  | small_scale_variant | FLCN | H429N | 0.50 (384/764) | 17 | 17216395 |
| Soft Tissue | Soft Tissue | 24 | FoundationOne CDx DX1 | 3.78Muts/Mb() | stable | copy_number_alter ation | CDKN2A | loss |  | 9 | 21961858-21998003 |
|  |  |  |  |  |  | copy_number_alter  ation | CDKN2B | loss |  | 9 | 21998749-22028932 |
|  |  |  |  |  |  | rearrangement | LMNA-NTRK1 | fusion |  | 1:01 | 156874863-  156875048:156135599-156135918 |
|  |  |  |  |  |  | small_scale_variant | ARFRP1 | E181_N190del | 0.27 (204/763) | 20 | 63700477 |
|  |  |  |  |  |  | small_scale_variant | ARFRP1 | H192fs*162 | 0.28 (215/759) | 20 | 63700473 |
|  |  |  |  |  |  | small_scale_variant | PIK3C2B | K837N | 0.60 (649/1085) | 1 | 204446123 |

|  |  |  |  |  |  | small_scale_variant | TIPARP | N447D | 0.49 (616/1248) | 3 | 156703515 |
| --- | --- | --- | --- | --- | --- | --- | --- | --- | --- | --- | --- |
| Thyroid | Papillary Thyroid Cancer | 66 | FoundationOne CDx DX1 | 0.00Muts/Mb() | stable | rearrangement | TPM3-NTRK1 | fusion |  | 1:01 | 156873733-  156874102:154169824-154170631 |
|  |  |  |  |  |  | small_scale_variant | HRAS | A122V | 0.49 | 11 | 533538 |
|  |  |  |  |  |  | small_scale_variant | NF1 | R1534* | 0.03 | 17 | 31261733 |
|  |  |  |  |  |  | small_scale_variant | NF1 | R1534* | 0.03 | 17 | 31261733 |
|  |  |  |  |  |  | small_scale_variant | NF1 | R1534* | 0.03 | 17 | 31261733 |
|  |  |  |  |  |  | small_scale_variant | NTRK1 | R593W | 0.46 | 1 | 156876544 |
|  |  |  |  |  |  | small_scale_variant | PDGFRB | R370C | 0.55 | 5 | 150132769 |
|  |  |  |  |  |  | small_scale_variant | RBM10 | R765* | 0.51 | X | 47185568 |
|  |  |  |  |  |  | small_scale_variant | RNF43 | R145Q | 0.53 | 17 | 58363542 |
|  |  |  |  |  |  | small_scale_variant | SPEN | P3120del | 0.46 | 1 | 15935598 |
|  |  |  |  |  |  | small_scale_variant | TERT | c.-79-45C>T | 0.3 | 5 | 1295113 |
|  |  |  |  |  |  | small_scale_variant | TSC1 | T417I | 0.52 | 9 | 132910584 |
| Bowel | Colorectal Adenocarcinoma | 71 | FoundationOne CDx DX1 | 4Muts/Mb() | stable | rearrangement | LMNA-NTRK1 | fusion |  | 1:01 | 156874879-  156875048:156135310-156135613 |
|  |  |  |  |  |  | small_scale_variant | ALK | R137S | 0.5 | 2 | 29920251 |
|  |  |  |  |  |  | small_scale_variant | CHEK1 | E76G | 0.37 | 11 | 125627768 |
|  |  |  |  |  |  | small_scale_variant | ERCC4 | L190Q | 0.46 | 16 | 13926741 |
|  |  |  |  |  |  | small_scale_variant | FBXW7 | S558F | 0.7 | 4 | 152324366 |
|  |  |  |  |  |  | small_scale_variant | KMT2A | G73E | 0.58 | 11 | 118436730 |
|  |  |  |  |  |  | small_scale_variant | RAD51D | V66M | 0.15 | 17 | 35118568 |
|  |  |  |  |  |  | small_scale_variant | SMO | P753L | 0.15 | 7 | 129212345 |
|  |  |  |  |  |  | small_scale_variant | SPEN | L2700_V2706del | 0.22 | 1 | 15934339 |
|  |  |  |  |  |  | small_scale_variant | SPEN | P2004L | 0.52 | 1 | 15932251 |
|  |  |  |  |  |  | small_scale_variant | SPEN | P3120del | 0.48 | 1 | 15935598 |
| Head and Neck | Salivary Carcinoma | 76 | FoundationOne CDx DX1 | 0Muts/Mb() | stable | rearrangement | ETV6 | truncation |  | 12:12 | 11880117-11880369:28335508-  28335713 |
|  |  |  |  |  |  | rearrangement | ETV6-NTRK3 | fusion |  | 15:12 | 87957489-87957489:11877681-  11877681 |
|  |  |  |  |  |  | small_scale_variant | CDKN2A | H83D | 0.04 | 9 | 21971112 |
|  |  |  |  |  |  | small_scale_variant | MAP3K1 | R1238K | 0.51 | 5 | 56883573 |
|  |  |  |  |  |  | small_scale_variant | NF1 | T1648A | 0.47 | 17 | 31325926 |
|  |  |  |  |  |  | small_scale_variant | SDHD | V111I | 0.44 | 11 | 112094821 |
|  |  |  |  |  |  | small_scale_variant | TERT | c.-79-45C>T | 0.29 | 5 | 1295113 |
| Skin | Spitzoid Melanoma | 4 | FoundationOne CDx DX1 | 5.04Muts/Mb() | stable | rearrangement | LMNA-NTRK1 | fusion |  | 1:01 | 156874628-  156874713:156133934-156134275 |
|  |  |  |  |  |  | small_scale_variant | ATR | D817E | 0.51 (553/1083) | 3 | 142553906 |
|  |  |  |  |  |  | small_scale_variant | LTK | G212_G213insGG G | 0.56 (69/124) | 15 | 41511835 |
|  |  |  |  |  |  | small_scale_variant | MAF | G152S | 0.67 (8/12) | 16 | 79599449 |

| Head and Neck | Mammary Analogue Secretory Carcinoma  of Salivary Gland Origin | 32 | FoundationOne  CDx DX1 | 1.26Muts/Mb() | stable | copy_number_alter  ation | AKT3 | amplification |  | 1 | 243455698-243893220 |
| --- | --- | --- | --- | --- | --- | --- | --- | --- | --- | --- | --- |
|  |  |  |  |  |  | copy_number_alter  ation | BTG2 | amplification |  | 1 | 203305606-203307438 |
|  |  |  |  |  |  | copy_number_alter  ation | CDC73 | amplification |  | 1 | 193122200-193250758 |
|  |  |  |  |  |  | copy_number_alter  ation | DDR2 | amplification |  | 1 | 162719039-162780246 |
|  |  |  |  |  |  | copy_number_alter  ation | FH | amplification |  | 1 | 241497827-241519722 |
|  |  |  |  |  |  | copy_number_alter  ation | IKBKE | amplification |  | 1 | 206473206-206496192 |
|  |  |  |  |  |  | copy_number_alter  ation | MCL1 | amplification |  | 1 | 150538659-150628334 |
|  |  |  |  |  |  | copy_number_alter  ation | MDM4 | amplification |  | 1 | 204525492-204549682 |
|  |  |  |  |  |  | copy_number_alter  ation | PIK3C2B | amplification |  | 1 | 204424851-204469802 |
|  |  |  |  |  |  | copy_number_alter  ation | SDHC | amplification |  | 1 | 161314350-161364810 |
|  |  |  |  |  |  | rearrangement | ETV6-NTRK3 | fusion |  | 15:12 | 87961371-87961371:11882828-  11882828 |
|  |  |  |  |  |  | small_scale_variant | CDH1 | K182N | 0.35 (324/915) | 16 | 68808707 |
|  |  |  |  |  |  | small_scale_variant | NOTCH1 | Q2444* | 0.20 (119/586) | 9 | 136496409 |
|  |  |  |  |  |  | small_scale_variant | NOTCH3 | G1347R | 0.50 (103/207) | 19 | 15177889 |
|  |  |  |  |  |  | small_scale_variant | TSC2 | A678T | 0.38 (215/573) | 16 | 2071869 |
| Soft Tissue | Infantile Fibrosarcoma | 1 | FoundationOne CDx DX2 | 0Muts/Mb() | stable | rearrangement | LMNA-NTRK1 | fusion |  | 1:01 | 156874351-  156874654:156135647-156136034 |
|  |  |  |  |  |  | small_scale_variant | ERBB2 | V1253M | 0.485 | 17 | 39728033 |
|  |  |  |  |  |  | small_scale_variant | MAP3K13 | I523V | 0.4812 | 3 | 185466887 |
|  |  |  |  |  |  | small_scale_variant | MET | R1022Q | 0.4902 | 7 | 116774917 |
|  |  |  |  |  |  | small_scale_variant | PALB2 | G514R | 0.4793 | 16 | 23635006 |
| Bone | Chondroblastic Osteosarcoma | 16 | FoundationOne CDx DX2 | 1Muts/Mb() | stable | copy_number_alter ation | BTG1 | amplification |  | 12 | 92144079-92145535 |
|  |  |  |  |  |  | copy_number_alter  ation | CALR | amplification |  | 19 | 12938660-12943913 |
|  |  |  |  |  |  | copy_number_alter  ation | CDKN2A | amplification |  | 9 | 21853213-21998003 |
|  |  |  |  |  |  | copy_number_alter  ation | CDKN2B | amplification |  | 9 | 21998749-22101833 |
|  |  |  |  |  |  | copy_number_alter  ation | MTAP | amplification |  | 9 | 21802700-21862059 |
|  |  |  |  |  |  | copy_number_alter  ation | NBN | amplification |  | 8 | 89935532-89984607 |
|  |  |  |  |  |  | copy_number_alter  ation | RB1 | loss |  | 13 | 48476700-48480098 |
|  |  |  |  |  |  | copy_number_alter  ation | TEK | amplification |  | 9 | 27109551-27229259 |
|  |  |  |  |  |  | rearrangement | PHF20-NTRK1 | fusion |  | 1:20 | 156868002-156868196:35829573-  35829751 |
|  |  |  |  |  |  | small_scale_variant | ERCC4 | R670Q | 0.4569 | 16 | 13944827 |
|  |  |  |  |  |  | small_scale_variant | MAP3K1 | L1321P | 0.1815 | 5 | 56884806 |
|  |  |  |  |  |  | small_scale_variant | TP53 | P278S | 0.8982 | 17 | 7673788 |

| Prostate | Prostate Adenocarcinoma | 65 | OncoGuideTM  NCC Oncopanel system | 2.30Muts/Mb() | 1.2%(stab le) | rearrangement | AGTPBP1- NTRK2 | fusion | 0.4068 | 9:09 | 85704400-85704403:84921067-  84921070 |
| --- | --- | --- | --- | --- | --- | --- | --- | --- | --- | --- | --- |
|  |  |  |  |  |  | small_scale_variant | FLT3 | T820N | 0.481 (558/1161) | 13 | 28018549 |
|  |  |  |  |  |  | small_scale_variant | POLE | V437M | 0.474 (307/648) | 12 | 132673625 |
|  |  |  |  |  |  | small_scale_variant | NRG1 | T202M | 0.266 (258/971) | 8 | 32648322 |
|  |  |  |  |  |  | small_scale_variant | TP53 | Y163N | 0.435 (864/1986) | 17 | 7675125 |
| Esophagus/Stomach |  | 72 | FoundationOne CDx DX1 | 1.26Muts/Mb() | stable | copy_number_alter ation | CCNE1 | amplification |  | 19 | 29763011-29869731 |
|  |  |  |  |  |  | copy_number_alter  ation | ERBB2 | amplification |  | 17 | 39651436-39777579 |
|  |  |  |  |  |  | rearrangement | NTRK1-BCAN | fusion |  | 1:01 | 156864324-  156864500:156646671-156646808 |
|  |  |  |  |  |  | small_scale_variant | FLT1 | A664P | 0.18 (108/616) | 13 | 28389775 |
|  |  |  |  |  |  | small_scale_variant | JAK3 | P84L | 0.30 (150/506) | 19 | 17843834 |
|  |  |  |  |  |  | small_scale_variant | MSH3 | A62_P63insPAAP AA | 0.18 (73/416) | 5 | 80654916 |
|  |  |  |  |  |  | small_scale_variant | MTOR | P1408S | 0.48 (239/501) | 1 | 11199289 |
|  |  |  |  |  |  | small_scale_variant | NOTCH3 | R107W | 0.48 (182/379) | 19 | 15192398 |
|  |  |  |  |  |  | small_scale_variant | PTCH1 | Y922S | 0.58 (355/612) | 9 | 95459722 |
|  |  |  |  |  |  | small_scale_variant | TP53 | R273C | 0.54 (352/655) | 17 | 7673803 |
|  |  |  |  |  |  | small_scale_variant | TP53 | R273C | 0.54 (352/655) | 17 | 7673803 |
| Soft Tissue |  | 1 | FoundationOne CDx DX1 | 0.0Muts/Mb() | stable | rearrangement | NTRK3-ETV6 | fusion |  | 15:12 | 87955415-87955647:11872770-  11873019 |
|  |  |  |  |  |  | small_scale_variant | ERCC4 | A596E | 0.48 (387/809) | 16 | 13935719 |
|  |  |  |  |  |  | small_scale_variant | PARP2 | I331T | 0.49 (511/1038) | 14 | 20355802 |
|  |  |  |  |  |  | small_scale_variant | PIK3CA | I889M | 0.50 (625/1262) | 3 | 179230004 |
|  |  |  |  |  |  | small_scale_variant | RAD51D | V66M | 0.51 (385/757) | 17 | 35118568 |
| Head and Neck |  | 77 | FoundationOne CDx DX1 | 1.26Muts/Mb() | stable | rearrangement | ETV6-NTRK3 | fusion |  | 15:12 | 87945719-87945876:11879665-  11879905 |
|  |  |  |  |  |  | small_scale_variant | ERBB2 | S963C | 0.51 (643/1266) | 17 | 39726577 |
|  |  |  |  |  |  | small_scale_variant | GSK3B | R180Q | 0.26 (282/1089) | 3 | 119916113 |
|  |  |  |  |  |  | small_scale_variant | IKBKE | G660E | 0.47 (580/1233) | 1 | 206493312 |
|  |  |  |  |  |  | small_scale_variant | MAP3K13 | I523V | 0.49 (633/1286) | 3 | 185466887 |
|  |  |  |  |  |  | small_scale_variant | MET | N375K | 0.47 (478/1016) | 7 | 116700209 |
|  |  |  |  |  |  | small_scale_variant | NOTCH1 | R1303H | 0.48 (474/991) | 9 | 136506633 |
|  |  |  |  |  |  | small_scale_variant | NOTCH2 | S2103Y | 0.50 (692/1397) | 1 | 119916414 |
|  |  |  |  |  |  | small_scale_variant | TERT | c.-79-45C>T | 0.22 (61/273) | 5 | 1295113 |
| Head and Neck | Myoepithelial Carcinoma | 53 | FoundationOne Liquid CDx AB1 | 4Muts/Mb() | high not detected | rearrangement | ETV6-NTRK3 | fusion |  | 15:12 | 88065766-88065766:11876195-  11876195 |
|  |  |  |  |  |  | small_scale_variant | CARD11 | V171M | 0.51 | 7 | 2944385 |
|  |  |  |  |  |  | small_scale_variant | CHEK2 | A334V | 0 | 22 | 28699845 |
|  |  |  |  |  |  | small_scale_variant | CREBBP | R1768C | 0.01 | 16 | 3729745 |

|  |  |  |  |  |  | small_scale_variant | KDR | R833W | 0.01 | 4 | 55098149 |
| --- | --- | --- | --- | --- | --- | --- | --- | --- | --- | --- | --- |
|  |  |  |  |  |  | small_scale_variant | MSH6 | K1358fs*2 | 0.49 | 2 | 47806848 |
|  |  |  |  |  |  | small_scale_variant | NOTCH3 | R10_R13del | 0.65 | 19 | 15200865 |
|  |  |  |  |  |  | small_scale_variant | STK11 | F354L | 0.49 | 19 | 1223126 |
|  |  |  |  |  |  | small_scale_variant | TSC1 | T417I | 0.47 | 9 | 132910584 |
| Head and Neck | Mammary Analogue Secretory Carcinoma of Salivary Gland Origin | 66 | FoundationOne CDx DX1 | 1.26Muts/Mb() | stable | rearrangement | ETV6-NTRK3 | fusion |  | 15:12 | 88080068-88080068:11881807-  11881807 |
|  |  |  |  |  |  | small_scale_variant | CHEK2 | R521W | 0.47 | 22 | 28687968 |
|  |  |  |  |  |  | small_scale_variant | NBN | I171V | 0.48 | 8 | 89978293 |
|  |  |  |  |  |  | small_scale_variant | PRDM1 | Q634R | 0.23 | 6 | 106106498 |
|  |  |  |  |  |  | small_scale_variant | RICTOR | A373S | 0.47 | 5 | 38967371 |
|  |  |  |  |  |  | small_scale_variant | TSC2 | A77T | 0.49 | 16 | 2053345 |
| Bowel | Mucinous Adenocarcinoma of the Colon and Rectum | 57 | FoundationOne CDx DX1 | 22.70Muts/Mb(hi gh) | high | rearrangement | ETV6-NTRK3 | fusion |  | 15:12 | 88009148-88009148:11882371-  11882371 |
|  |  |  |  |  |  | small_scale_variant | AKT2 | S126P | 0.13 | 19 | 40242599 |
|  |  |  |  |  |  | small_scale_variant | ALK | R48H | 0.15 | 2 | 29920517 |
|  |  |  |  |  |  | small_scale_variant | ARID1A | D1850fs*33 | 0.15 | 1 | 26779445 |
|  |  |  |  |  |  | small_scale_variant | ATM | N1739fs*10 | 0.14 | 11 | 108301686 |
|  |  |  |  |  |  | small_scale_variant | AXIN1 | G265fs*149 | 0.28 | 16 | 346239 |
|  |  |  |  |  |  | small_scale_variant | BAP1 | I586V | 0.16 | 3 | 52403272 |
|  |  |  |  |  |  | small_scale_variant | CASP8 | G342D | 0.17 | 2 | 201284987 |
|  |  |  |  |  |  | small_scale_variant | CIC | P1248fs*54 | 0.12 | 19 | 42293228 |
|  |  |  |  |  |  | small_scale_variant | CREBBP | R2052W | 0.34 | 16 | 3728893 |
|  |  |  |  |  |  | small_scale_variant | DOT1L | S615L | 0.11 | 19 | 2214517 |
|  |  |  |  |  |  | small_scale_variant | FANCC | K231del | 0.14 | 9 | 95135494 |
|  |  |  |  |  |  | small_scale_variant | FBXW7 | W425* | 0.16 | 4 | 152328352 |
|  |  |  |  |  |  | small_scale_variant | IKBKE | G660E | 0.47 | 1 | 206493312 |
|  |  |  |  |  |  | small_scale_variant | JAK1 | R93C | 0.15 | 1 | 64879077 |
|  |  |  |  |  |  | small_scale_variant | JAK3 | A919T | 0.12 | 19 | 17831724 |
|  |  |  |  |  |  | small_scale_variant | MAP3K1 | T948_T949del | 0.11 | 5 | 56882041 |
|  |  |  |  |  |  | small_scale_variant | MSH2 | K845E | 0.47 | 2 | 47480770 |
|  |  |  |  |  |  | small_scale_variant | NBN | R466fs*18 | 0.17 | 8 | 89955283 |
|  |  |  |  |  |  | small_scale_variant | NOTCH3 | A1450T | 0.51 | 19 | 15177580 |
|  |  |  |  |  |  | small_scale_variant | NSD3 | S88T | 0.17 | 8 | 38347910 |
|  |  |  |  |  |  | small_scale_variant | P2RY8 | R234H | 0.14 | X | 1465858 |
|  |  |  |  |  |  | small_scale_variant | QKI | K134fs*14 | 0.16 | 6 | 163478894 |
|  |  |  |  |  |  | small_scale_variant | RAD51B | E193D | 0.48 | 14 | 67887027 |

|  |  |  |  |  |  | small_scale_variant | RNF43 | G659fs*41 | 0.17 | 17 | 58357799 |
| --- | --- | --- | --- | --- | --- | --- | --- | --- | --- | --- | --- |
|  |  |  |  |  |  | small_scale_variant | TGFBR2 | K128fs*35 | 0.45 | 3 | 30650388 |
|  |  |  |  |  |  | small_scale_variant | WT1 | A83T | 0.23 | 11 | 32435114 |
| CNS/Brain | Pilocytic Astrocytoma | 0 | FoundationOne CDx DX2 | 0Muts/Mb() | stable | rearrangement | C1orf226- NTRK1 | fusion |  | 1:01 | 156873792-  156874098:162379337-162379773 |
|  |  |  |  |  |  | rearrangement | NTRK1 | rearrangements |  | 1:01 | 156871474-  156871836:162494801-162495219 |
|  |  |  |  |  |  | rearrangement | NTRK1 | rearrangements |  | 1:01 | 156871857-  156871975:156195322-156195477 |
|  |  |  |  |  |  | rearrangement | NTRK1 | rearrangements |  | 1:01 | 156871862-  156871969:162420136-162420438 |
|  |  |  |  |  |  | rearrangement | NTRK1-  SLC25A44 | fusion |  | 1:01 | 156872233-  156872666:156194668-156194899 |
|  |  |  |  |  |  | small_scale_variant | CARD11 | T670M | 0.4526 | 7 | 2923265 |
|  |  |  |  |  |  | small_scale_variant | EMSY | R62H | 0.4721 | 11 | 76453328 |
|  |  |  |  |  |  | small_scale_variant | RAD51D | I311N | 0.4728 | 17 | 35101008 |
|  |  |  |  |  |  | small_scale_variant | TET2 | E259K | 0.4851 | 4 | 105234717 |
|  |  |  |  |  |  | small_scale_variant | TSC2 | A862V | 0.5107 | 16 | 2075838 |
|  |  |  |  |  |  | small_scale_variant | ZNF217 | T239S | 0.4897 | 20 | 53582112 |
| Head and Neck | Mammary Analogue Secretory Carcinoma of Salivary Gland Origin | 75 | OncoGuideTM NCC Oncopanel system | 0.00Muts/Mb() | 1.79%(sta ble) | rearrangement | ETV6-NTRK3 | fusion | 0.3111 | 12:15 | 11881663-11881663:87944722-  87944722 |
|  |  |  |  |  |  | rearrangement | NTRK3-ETV6 | fusion | 0.152 | 15:12 | 87945311-87945312:11886098-  11886099 |
|  |  |  |  |  |  | small_scale_variant | ABL1 | P662L | 0.496  (1289/2597) | 9 | 130884275 |
|  |  |  |  |  |  | small_scale_variant | MAP3K1 | V889L | 0.454 (270/595) | 5 | 56881865 |
|  |  |  |  |  |  | small_scale_variant | MSH2 | A733T | 0.51 (562/1103) | 2 | 47476558 |
|  |  |  |  |  |  | small_scale_variant | ROS1 | L590P | 0.451 (836/1852) | 6 | 117388025 |
| Soft Tissue |  | 66 | FoundationOne CDx DX1 | 3.78Muts/Mb() | stable | rearrangement | TPM3-NTRK1 | fusion |  | 1:01 | 156874448-  156874712:154165404-154165659 |
|  |  |  |  |  |  | small_scale_variant | ARID1A | D1850fs*4 | 0.20 (192/954) | 1 | 26779446 |
|  |  |  |  |  |  | small_scale_variant | ARID1A | S1992P | 0.19 (193/1039) | 1 | 26779872 |
|  |  |  |  |  |  | small_scale_variant | CD79B | R113Q | 0.48 (482/1011) | 17 | 63930166 |
|  |  |  |  |  |  | small_scale_variant | CIC | N702S | 0.49 (499/1008) | 19 | 42290873 |
|  |  |  |  |  |  | small_scale_variant | ERBB2 | V1253M | 0.50 (358/713) | 17 | 39728033 |
|  |  |  |  |  |  | small_scale_variant | POLE | R1382C | 0.51 (338/658) | 12 | 132648934 |
|  |  |  |  |  |  | small_scale_variant | RAC1 | I21N | 0.21 (184/896) | 7 | 6387238 |
|  |  |  |  |  |  | small_scale_variant | SMARCA4 | G1162S | 0.20 (99/499) | 19 | 11030831 |
| Head and Neck |  | 54 | FoundationOne CDx DX1 | 1.26Muts/Mb() | stable | rearrangement | ETV6-NTRK3 | fusion |  | 12:15 | 11879151-11880392:87962081-  87962476 |
|  |  |  |  |  |  | small_scale_variant | ATM | R337C | 0.02 (20/872) | 11 | 108247071 |
|  |  |  |  |  |  | small_scale_variant | ATM | R924Q | 0.51 (443/864) | 11 | 108268542 |
|  |  |  |  |  |  | small_scale_variant | GATA3 | A396T | 0.51 (446/874) | 10 | 8073874 |

|  |  |  |  |  |  | small_scale_variant | GATA4 | P163S | 0.57 (131/230) | 8 | 11708799 |
| --- | --- | --- | --- | --- | --- | --- | --- | --- | --- | --- | --- |
|  |  |  |  |  |  | small_scale_variant | IKBKE | G660E | 0.48 (303/626) | 1 | 206493312 |
|  |  |  |  |  |  | small_scale_variant | KMT2D | E1535A | 0.46 (327/710) | 12 | 49046154 |
|  |  |  |  |  |  | small_scale_variant | NOTCH1 | Q1134R | 0.49 (308/624) | 9 | 136508064 |
|  |  |  |  |  |  | small_scale_variant | SETD2 | D631fs*11 | 0.22 (210/934) | 3 | 47122745 |
|  |  |  |  |  |  | small_scale_variant | TERT | c.-79-45C>T | 0.24 (40/170) | 5 | 1295113 |
|  |  |  |  |  |  | small_scale_variant | TSC2 | E1789V | 0.48 (333/693) | 16 | 2088552 |
| Uterus | Uterine Epithelioid Leiomyosarcoma | 59 | FoundationOne CDx DX1 | 2.52Muts/Mb() | stable | copy_number_alter ation | BRCA2 | loss |  | 13 | 32258204-32424884 |
|  |  |  |  |  |  | copy_number_alter  ation | IDH1 | loss |  | 2 | 208237059-208251554 |
|  |  |  |  |  |  | copy_number_alter  ation | RB1 | loss |  | 13 | 48411294-48627034 |
|  |  |  |  |  |  | copy_number_alter  ation | TP53 | loss |  | 17 | 7666160-7670726 |
|  |  |  |  |  |  | rearrangement | DCAF12-  NTRK2 | fusion |  | 9:09 | 84744313-84744635:34102037-  34102302 |
|  |  |  |  |  |  | small_scale_variant | ALK | E314G | 0.49 (372/765) | 2 | 29694861 |
|  |  |  |  |  |  | small_scale_variant | ATRX | L1233* | 0.36 (337/946) | X | 77681558 |
|  |  |  |  |  |  | small_scale_variant | BRAF | D22N | 0.47 (417/893) | 7 | 140924640 |
|  |  |  |  |  |  | small_scale_variant | CDH1 | V832M | 0.70 (435/619) | 16 | 68833344 |
|  |  |  |  |  |  | small_scale_variant | CDK6 | L166fs*45 | 0.12 (178/1501) | 7 | 92725653 |
|  |  |  |  |  |  | small_scale_variant | CIC | A1014V | 0.48 (253/524) | 19 | 42292332 |
|  |  |  |  |  |  | small_scale_variant | FGF6 | S143N | 0.37 (234/627) | 12 | 4444155 |
|  |  |  |  |  |  | small_scale_variant | IKBKE | G660E | 0.29 (200/684) | 1 | 206493312 |
|  |  |  |  |  |  | small_scale_variant | PDCD1LG2 | c.632-1G>C | 0.37 (362/982) | 9 | 5557617 |
|  |  |  |  |  |  | small_scale_variant | SPEN | P3120del | 0.41 (343/834) | 1 | 15935598 |
| Breast |  | 58 | OncoGuideTM NCC Oncopanel system | 2.3Muts/Mb() |  | rearrangement | TPM3-NTRK1 | fusion |  | 1:01 | 154162195-  154162196:156874430-156874431 |
|  |  |  |  |  |  | small_scale_variant | ARID1A | C1968fs*30 | 0.36 (1035/2883) | 1 | 26779797 |
| Soft Tissue |  | 4 | FoundationOne CDx DX1 | 0.0Muts/Mb() | stable | copy_number_alter ation | CDKN2A | loss |  | 9 | 21853213-21998003 |
|  |  |  |  |  |  | copy_number_alter  ation | CDKN2B | loss |  | 9 | 21998749-22101833 |
|  |  |  |  |  |  | copy_number_alter  ation | MTAP | loss |  | 9 | 21802700-21862059 |
|  |  |  |  |  |  | copy_number_alter  ation | PRKAR1A | amplification |  | 17 | 68515399-68530449 |
|  |  |  |  |  |  | rearrangement | LMNA-NTRK1 | fusion |  | 1:01 | 156874594-  156874712:156134358-156134690 |
|  |  |  |  |  |  | small_scale_variant | DAXX | E457del | 0.26 (525/2052) | 6 | 33320103 |
|  |  |  |  |  |  | small_scale_variant | ESR1 | A340V | 0.49 (501/1032) | 6 | 151944431 |
|  |  |  |  |  |  | small_scale_variant | MST1R | R396* | 0.54 (442/812) | 3 | 49902424 |
|  |  |  |  |  |  | small_scale_variant | NOTCH3 | G1347R | 0.53 (154/290) | 19 | 15177889 |

|  |  |  |  |  | small_scale_variant | RAD54L | H676fs*19 | 0.48 (495/1035) | 1 | 46277973 |
| --- | --- | --- | --- | --- | --- | --- | --- | --- | --- | --- |
| Breast | 70 | FoundationOne CDx DX1 | 6.3Muts/Mb() | stable | rearrangement | ETV6-NTRK3 | fusion |  | 15:12 | 88012285-88012934:11877374-  11878230 |
|  |  |  |  |  | small_scale_variant | BRD4 | P978_P980del | 0.35 (243/689) | 19 | 15243127 |
|  |  |  |  |  | small_scale_variant | CUL3 | R733Q | 0.23 (180/769) | 2 | 224474354 |
|  |  |  |  |  | small_scale_variant | ERBB3 | R1118Q | 0.43 (436/1018) | 12 | 56101212 |
|  |  |  |  |  | small_scale_variant | FANCG | A153G | 0.46 (367/791) | 9 | 35078193 |
|  |  |  |  |  | small_scale_variant | MAF | Q303E | 0.31 (242/780) | 16 | 79598996 |
|  |  |  |  |  | small_scale_variant | NOTCH3 | A1450T | 0.65 (444/686) | 19 | 15177580 |
|  |  |  |  |  | small_scale_variant | TERT | c.-79-45C>T | 0.16 (28/180) | 5 | 1295113 |
|  |  |  |  |  | small_scale_variant | TGFBR2 | A179fs*14 | 0.20 (162/809) | 3 | 30671718 |
|  |  |  |  |  | small_scale_variant | VEGFA | C386fs*14 | 0.23 (188/835) | 6 | 43782025 |
|  |  |  |  |  | small_scale_variant | VEGFA | S186Y | 0.52 (425/825) | 6 | 43784609 |
| Bowel | 42 | FoundationOne CDx DX1 | 5.04Muts/Mb() | stable | copy_number_alter ation | CDK8 | amplification |  | 13 | 26206195-26447079 |
|  |  |  |  |  | copy_number_alter  ation | DIS3 | amplification |  | 13 | 72759771-72781832 |
|  |  |  |  |  | rearrangement | NTRK3-  UNC13C | fusion |  | 15:15 | 87978320-87978452:54068256-  54068409 |
|  |  |  |  |  | small_scale_variant | APC | Y997fs*9 | 0.28 (215/777) | 5 | 112838583 |
|  |  |  |  |  | small_scale_variant | APC | Y997fs*9 | 0.28 (215/777) | 5 | 112838583 |
|  |  |  |  |  | small_scale_variant | BRD4 | V268I | 0.51 (403/793) | 19 | 15265401 |
|  |  |  |  |  | small_scale_variant | EPHB4 | R729G | 0.61 (757/1235) | 7 | 100807514 |
|  |  |  |  |  | small_scale_variant | IKBKE | G660E | 0.49 (447/921) | 1 | 206493312 |
|  |  |  |  |  | small_scale_variant | KDR | G1337V | 0.06 (63/1103) | 4 | 55080002 |
|  |  |  |  |  | small_scale_variant | KIT | T304A | 0.52 (479/930) | 4 | 54703877 |
|  |  |  |  |  | small_scale_variant | MAP3K1 | L78P | 0.53 (233/439) | 5 | 56815806 |
|  |  |  |  |  | small_scale_variant | NOTCH1 | D1185N | 0.42 (309/736) | 9 | 136507395 |
|  |  |  |  |  | small_scale_variant | PTCH1 | R1303C | 0.53 (345/653) | 9 | 95447349 |
|  |  |  |  |  | small_scale_variant | ROS1 | A2233S | 0.20 (217/1063) | 6 | 117301010 |
|  |  |  |  |  | small_scale_variant | SMAD4 | K122R | 0.06 (57/924) | 18 | 51048801 |
|  |  |  |  |  | small_scale_variant | SMAD4 | Y131N | 0.06 (49/809) | 18 | 51048827 |
|  |  |  |  |  | small_scale_variant | STK11 | R415C | 0.51 (310/605) | 19 | 1226588 |
|  |  |  |  |  | small_scale_variant | TP53 | E198* | 0.43 (456/1068) | 17 | 7674939 |
|  |  |  |  |  | small_scale_variant | TP53 | E198* | 0.43 (456/1068) | 17 | 7674939 |
|  |  |  |  |  | small_scale_variant | TP53 | E198* | 0.43 (456/1068) | 17 | 7674939 |
| Head and Neck | 79 | FoundationOne CDx DX1 | 3.78Muts/Mb() | stable | rearrangement | ETV6-NTRK3 | fusion |  | 15:12 | 87941440-87941649:11875386-  11875601 |
|  |  |  |  |  | small_scale_variant | MAP3K1 | R1238K | 0.48 (517/1086) | 5 | 56883573 |
|  |  |  |  |  | small_scale_variant | MYD88 | L252P | 0.02 (20/1100) | 3 | 38141150 |

|  |  |  |  |  |  | small_scale_variant | MYD88 | L252P | 0.02 (20/1100) | 3 | 38141150 |
| --- | --- | --- | --- | --- | --- | --- | --- | --- | --- | --- | --- |
|  |  |  |  |  |  | small_scale_variant | NOTCH1 | D1808N | 0.49 (583/1184) | 9 | 136502051 |
|  |  |  |  |  |  | small_scale_variant | NOTCH1 | I1445M | 0.16 (153/940) | 9 | 136505561 |
|  |  |  |  |  |  | small_scale_variant | NOTCH3 | G1347R | 0.48 (185/389) | 19 | 15177889 |
|  |  |  |  |  |  | small_scale_variant | SMAD2 | V405I | 0.15 (169/1150) | 18 | 47845407 |
|  |  |  |  |  |  | small_scale_variant | STK11 | F354L | 0.51 (653/1283) | 19 | 1223126 |
|  |  |  |  |  |  | small_scale_variant | STK11 | F354L | 0.51 (653/1283) | 19 | 1223126 |
|  |  |  |  |  |  | small_scale_variant | STK11 | F354L | 0.51 (653/1283) | 19 | 1223126 |
|  |  |  |  |  |  | small_scale_variant | TGFBR2 | S46R | 0.48 (491/1018) | 3 | 30623240 |
| Biliary Tract | Biliary Tract | 72 | FoundationOne CDx DX1 | 15.13Muts/Mb() | high | copy_number_alter ation | MLH1 | loss |  | 3 | 36874314-37129389 |
|  |  |  |  |  |  | rearrangement | NTRK3-MTOR | fusion |  | 15:01 | 88135133-88135412:11190795-  11191106 |
|  |  |  |  |  |  | small_scale_variant | ABL1 | R134H | 0.07 (98/1354) | 9 | 130854948 |
|  |  |  |  |  |  | small_scale_variant | ARID1A | F2141fs*59 | 0.35 (266/758) | 1 | 26780317 |
|  |  |  |  |  |  | small_scale_variant | ATR | A906T | 0.06 (76/1302) | 3 | 142553316 |
|  |  |  |  |  |  | small_scale_variant | BCORL1 | P206fs*47 | 0.52 (351/681) | X | 130013388 |
|  |  |  |  |  |  | small_scale_variant | CREBBP | L54fs*1 | 0.31 (310/1001) | 16 | 3850933 |
|  |  |  |  |  |  | small_scale_variant | CTNNB1 | T693S | 0.40 (295/733) | 3 | 41238016 |
|  |  |  |  |  |  | small_scale_variant | DOT1L | G288D | 0.11 (63/559) | 19 | 2207580 |
|  |  |  |  |  |  | small_scale_variant | DOT1L | P1019L | 0.10 (85/835) | 19 | 2222225 |
|  |  |  |  |  |  | small_scale_variant | DOT1L | S911L | 0.42 (241/579) | 19 | 2220148 |
|  |  |  |  |  |  | small_scale_variant | EP300 | T1021fs*3 | 0.26 (289/1104) | 22 | 41152268 |
|  |  |  |  |  |  | small_scale_variant | EPHA3 | T351R | 0.30 (354/1193) | 3 | 89341836 |
|  |  |  |  |  |  | small_scale_variant | GNAS | V61I | 0.26 (289/1119) | 20 | 58853446 |
|  |  |  |  |  |  | small_scale_variant | IRF2 | L313fs*5 | 0.19 (229/1212) | 4 | 184388870 |
|  |  |  |  |  |  | small_scale_variant | KDM6A | R1213* | 0.55 (252/456) | X | 45089831 |
|  |  |  |  |  |  | small_scale_variant | KDM6A | R1213* | 0.55 (252/456) | X | 45089831 |
|  |  |  |  |  |  | small_scale_variant | KDM6A | R1213* | 0.55 (252/456) | X | 45089831 |
|  |  |  |  |  |  | small_scale_variant | KMT2D | K1686fs*36 | 0.16 (196/1231) | 12 | 49044427 |
|  |  |  |  |  |  | small_scale_variant | MSH2 | G40S | 0.55 (513/937) | 2 | 47403309 |
|  |  |  |  |  |  | small_scale_variant | MUTYH | c.892-2A>G | 0.48 (579/1216) | 1 | 45332088 |
|  |  |  |  |  |  | small_scale_variant | NF1 | A804V | 0.51 (382/753) | 17 | 31229026 |
|  |  |  |  |  |  | small_scale_variant | NOTCH3 | R1895C | 0.18 (155/860) | 19 | 15165500 |
|  |  |  |  |  |  | small_scale_variant | PBRM1 | R926fs*82 | 0.35 (217/614) | 3 | 52603523 |
|  |  |  |  |  |  | small_scale_variant | PIK3CB | C287R | 0.30 (341/1144) | 3 | 138734747 |
|  |  |  |  |  |  | small_scale_variant | PTCH1 | G17dup | 0.32 (71/221) | 9 | 95508310 |

|  |  |  |  |  |  | small_scale_variant | RICTOR | L1537R | 0.43 (577/1335) | 5 | 38945514 |
| --- | --- | --- | --- | --- | --- | --- | --- | --- | --- | --- | --- |
|  |  |  |  |  |  | small_scale_variant | RPTOR | T1011M | 0.07 (43/597) | 17 | 80945673 |
|  |  |  |  |  |  | small_scale_variant | SPEN | A1665V | 0.11 (150/1387) | 1 | 15931234 |
| Head and Neck | Mammary Analogue Secretory Carcinoma of Salivary Gland Origin | 42 | FoundationOne CDx DX1 | 0.0Muts/Mb() | stable | rearrangement | ETV6-NTRK3 | fusion |  | 15:12 | 88016747-88017147:11879325-  11879802 |
|  |  |  |  |  |  | small_scale_variant | ATM | T935R | 0.43 (384/896) | 11 | 108268575 |
|  |  |  |  |  |  | small_scale_variant | FGFR3 | L164V | 0.51 (321/626) | 4 | 1801411 |
|  |  |  |  |  |  | small_scale_variant | NOTCH2 | L2135fs*7 | 0.15 (184/1196) | 1 | 119916288 |
|  |  |  |  |  |  | small_scale_variant | STK11 | F354L | 0.50 (439/882) | 19 | 1223126 |
| Head and Neck | Acinic Cell Carcinoma | 55 | FoundationOne CDx DX1 | 1.26Muts/Mb() | stable | rearrangement | ETV6-NTRK3 | fusion |  | 15:12 | 87942325-87942325:11882752-  11882752 |
|  |  |  |  |  |  | small_scale_variant | BRAF | P403A | 0.54 (539/1001) | 7 | 140783128 |
|  |  |  |  |  |  | small_scale_variant | ERBB2 | S1050L | 0.55 (488/887) | 17 | 39726993 |
|  |  |  |  |  |  | small_scale_variant | GID4 | V148M | 0.59 (596/1005) | 17 | 18045150 |
|  |  |  |  |  |  | small_scale_variant | IGF1R | F75L | 0.49 (519/1068) | 15 | 98707690 |
|  |  |  |  |  |  | small_scale_variant | IGF1R | P853S | 0.48 (369/776) | 15 | 98923947 |
|  |  |  |  |  |  | small_scale_variant | PDK1 | D47E | 0.47 (305/647) | 2 | 172556291 |
|  |  |  |  |  |  | small_scale_variant | RPTOR | R276H | 0.17 (176/1009) | 17 | 80754182 |
|  |  |  |  |  |  | small_scale_variant | SETD2 | S1769Y | 0.46 (568/1224) | 3 | 47086286 |
|  |  |  |  |  |  | small_scale_variant | TERT | c.-79-67C>T | 0.21 (30/144) | 5 | 1295135 |
| Cervix |  | 59 | FoundationOne CDx DX1 | 1.26Muts/Mb() | stable | copy_number_alter ation | CDKN2A | loss |  | 9 | 21853213-21998003 |
|  |  |  |  |  |  | copy_number_alter  ation | CDKN2B | loss |  | 9 | 21998749-22012553 |
|  |  |  |  |  |  | copy_number_alter  ation | MTAP | loss |  | 9 | 21837894-21862059 |
|  |  |  |  |  |  | rearrangement | ETV6-NTRK3 | fusion |  | 15:12 | 88019066-88019273:11876066-  11876487 |
|  |  |  |  |  |  | small_scale_variant | BRCA2 | G2508S | 0.50 (477/961) | 13 | 32356514 |
|  |  |  |  |  |  | small_scale_variant | CDK4 | R24C | 0.43 (447/1040) | 12 | 57751648 |
|  |  |  |  |  |  | small_scale_variant | DOT1L | A528V | 0.50 (209/415) | 19 | 2213564 |
|  |  |  |  |  |  | small_scale_variant | PTEN | c.253+2T>C | 0.67 (494/733) | 10 | 87931091 |
|  |  |  |  |  |  | small_scale_variant | STAT3 | G342S | 0.50 (317/638) | 17 | 42333698 |
|  |  |  |  |  |  | small_scale_variant | SUFU | E152K | 0.64 (221/348) | 10 | 102550106 |
| Thyroid | Papillary Thyroid Cancer | 58 | OncoGuideTM NCC Oncopanel system | 0.80Muts/Mb() | 0.0%(stab le) | rearrangement | TPR-NTRK1 | fusion | 0.14 | 1:01 | 186349028-  186349028:156874501-156874501 |
|  |  |  |  |  |  | small_scale_variant | ERBB2 | S1096L | 0.49 (1506/3079) | 17 | 39726993 |
|  |  |  |  |  |  | small_scale_variant | MAP3K1 | V889L | 0.48 (259/544) | 5 | 56881865 |
| Biliary Tract | Gallbladder Adenocarcinoma, NOS | 69 | FoundationOne CDx DX1 | 4Muts/Mb() | stable | copy_number_alter ation | ABL1 | amplification |  | 9 | 130714319-130885683 |
|  |  |  |  |  |  | copy_number_alter  ation | DDR2 | amplification |  | 1 | 162719039-162780246 |

|  |  |  |  |  |  | copy_number_alter  ation | KDR | amplification |  | 4 | 55030470-55172588 |
| --- | --- | --- | --- | --- | --- | --- | --- | --- | --- | --- | --- |
|  |  |  |  |  |  | copy_number_alter  ation | LYN | amplification |  | 8 | 55941859-56010110 |
|  |  |  |  |  |  | copy_number_alter  ation | MCL1 | amplification |  | 1 | 150538659-150628334 |
|  |  |  |  |  |  | copy_number_alter  ation | MDM2 | amplification |  | 12 | 68760216-68883425 |
|  |  |  |  |  |  | copy_number_alter  ation | MDM2 | amplification |  | 12 | 68760216-68883425 |
|  |  |  |  |  |  | copy_number_alter  ation | MYC | amplification |  | 8 | 127694344-127789205 |
|  |  |  |  |  |  | copy_number_alter  ation | NBN | amplification |  | 8 | 89935532-89984607 |
|  |  |  |  |  |  | copy_number_alter  ation | NFE2L2 | amplification |  | 2 | 177230784-177264618 |
|  |  |  |  |  |  | copy_number_alter  ation | NTRK1 | amplification |  | 1 | 156815772-156881642 |
|  |  |  |  |  |  | copy_number_alter  ation | RAD21 | amplification |  | 8 | 116848922-116866729 |
|  |  |  |  |  |  | copy_number_alter  ation | REL | amplification |  | 2 | 60881780-60922535 |
|  |  |  |  |  |  | copy_number_alter  ation | SDHC | amplification |  | 1 | 161314350-161364810 |
|  |  |  |  |  |  | copy_number_alter  ation | TSC1 | amplification |  | 9 | 132849505-132987375 |
|  |  |  |  |  |  | copy_number_alter  ation | XPO1 | amplification |  | 2 | 61478819-61533899 |
|  |  |  |  |  |  | rearrangement | EMSY | rearrangements |  | 11:11 | 76451789-76452079:76549215-  76549532 |
|  |  |  |  |  |  | rearrangement | EMSY | rearrangements |  | 11:11 | 76451801-76452058:56192293-  56192445 |
|  |  |  |  |  |  | rearrangement | EMSY | rearrangements |  | 11:11 | 76463873-76464195:76635113-  76635408 |
|  |  |  |  |  |  | rearrangement | PRRC2C-  NTRK1 | fusion |  | 1:01 | 156868042-  156868334:171519490-171519888 |
|  |  |  |  |  |  | small_scale_variant | BRCA2 | V2503I | 0.45 | 13 | 32356499 |
|  |  |  |  |  |  | small_scale_variant | EMSY | V739A | 0.37 | 11 | 76535916 |
|  |  |  |  |  |  | small_scale_variant | MAF | Y181dup | 0.07 | 16 | 79599359 |
|  |  |  |  |  |  | small_scale_variant | MSH3 | A62_P63insPAAP AA | 0.4 | 5 | 80654916 |
|  |  |  |  |  |  | small_scale_variant | STK11 | I238F | 0.55 | 19 | 1220695 |
| Skin | Sweat Gland Carcinoma/Apocrine Eccrine Carcinoma | 61 | FoundationOne CDx DX1 | 1Muts/Mb() | stable | rearrangement | ETV6 | truncation |  | 12:02 | 11872321-11872475:68683798-  68683952 |
|  |  |  |  |  |  | rearrangement | ETV6-NTRK3 | fusion |  | 15:12 | 87951486-87951486:11872366-  11872366 |
|  |  |  |  |  |  | small_scale_variant | ARID1A | A2181fs*39 | 0.21 | 1 | 26780437 |
|  |  |  |  |  |  | small_scale_variant | BRCA1 | L52F | 0.47 | 17 | 43106514 |
|  |  |  |  |  |  | small_scale_variant | DIS3 | E466K | 0.02 | 13 | 72772176 |
|  |  |  |  |  |  | small_scale_variant | DIS3 | N630D | 0.5 | 13 | 72763600 |
|  |  |  |  |  |  | small_scale_variant | FLT1 | P441L | 0.48 | 13 | 28427273 |
|  |  |  |  |  |  | small_scale_variant | MUTYH | c.892-2A>G | 0.48 | 1 | 45332088 |
|  |  |  |  |  |  | small_scale_variant | PARP1 | T867A | 0.34 | 1 | 226365061 |

|  |  |  |  |  |  | small_scale_variant | PIK3CA | H1047R | 0.03 | 3 | 179234297 |
| --- | --- | --- | --- | --- | --- | --- | --- | --- | --- | --- | --- |
|  |  |  |  |  |  | small_scale_variant | SF3B1 | G740E | 0.03 | 2 | 197401989 |
|  |  |  |  |  |  | small_scale_variant | SOX9 | P360_P366del | 0.45 | 17 | 72123936 |
|  |  |  |  |  |  | small_scale_variant | STAG2 | c.2097-2A>G | 0.38 | X | 124066173 |
|  |  |  |  |  |  | small_scale_variant | XPO1 | T576I | 1 | 2 | 61492195 |
| Other | Adenocarcinoma, NOS | 55 | FoundationOne CDx DX2 | 14Muts/Mb(high) | stable | copy_number_alter ation | BARD1 | amplification |  | 2 | 214728675-214809569 |
|  |  |  |  |  |  | copy_number_alter  ation | ERBB4 | amplification |  | 2 | 211383614-212124903 |
|  |  |  |  |  |  | copy_number_alter  ation | KMT2D | amplification |  | 12 | 49021761-49055364 |
|  |  |  |  |  |  | copy_number_alter  ation | KRAS | amplification |  | 12 | 25191796-25295283 |
|  |  |  |  |  |  | copy_number_alter  ation | PIK3C2G | amplification |  | 12 | 18282081-18648028 |
|  |  |  |  |  |  | copy_number_alter  ation | TGFBR2 | amplification |  | 3 | 30606868-30691599 |
|  |  |  |  |  |  | rearrangement | CHIC1-NTRK1 | fusion |  | 1:X | 156841951-156842213:73678900-  73679033 |
|  |  |  |  |  |  | small_scale_variant | ABL1 | W476C | 0.1253 | 9 | 130880072 |
|  |  |  |  |  |  | small_scale_variant | DAXX | E451del | 0.4981 | 6 | 33320120 |
|  |  |  |  |  |  | small_scale_variant | FANCL | A143V | 0.3103 | 2 | 58204173 |
|  |  |  |  |  |  | small_scale_variant | FGF3 | L152P | 0.5142 | 11 | 69810570 |
|  |  |  |  |  |  | small_scale_variant | FGFR2 | A511T | 0.4196 | 10 | 121500856 |
|  |  |  |  |  |  | small_scale_variant | FGFR3 | L164V | 0.5084 | 4 | 1801411 |
|  |  |  |  |  |  | small_scale_variant | KEAP1 | G417W | 0.4147 | 19 | 10491653 |
|  |  |  |  |  |  | small_scale_variant | MSH2 | T754A | 0.691 | 2 | 47478321 |
|  |  |  |  |  |  | small_scale_variant | MYCN | A184S | 0.6061 | 2 | 15942614 |
|  |  |  |  |  |  | small_scale_variant | NOTCH1 | A1458T | 0.085 | 9 | 136505524 |
|  |  |  |  |  |  | small_scale_variant | NOTCH2 | E610K | 0.0779 | 1 | 119963661 |
|  |  |  |  |  |  | small_scale_variant | PARP1 | D307N | 0.273 | 1 | 226385596 |
|  |  |  |  |  |  | small_scale_variant | PDCD1 | T36fs*70 | 0.0571 | 2 | 241852951 |
|  |  |  |  |  |  | small_scale_variant | PIK3C2G | P628A | 0.0538 | 12 | 18391131 |
|  |  |  |  |  |  | small_scale_variant | PIK3CB | R321Q | 0.4883 | 3 | 138734644 |
|  |  |  |  |  |  | small_scale_variant | POLE | I514L | 0.253 | 12 | 132672773 |
|  |  |  |  |  |  | small_scale_variant | SMARCA4 | Q1166* | 0.4185 | 19 | 11030843 |
|  |  |  |  |  |  | small_scale_variant | TP53 | G154fs*16 | 0.3551 | 17 | 7675150 |
| Kidney | Unclassified Renal Cell Carcinoma | 69 | FoundationOne CDx DX1 | 2.52Muts/Mb() | stable | copy_number_alter ation | CBFB | amplification |  | 16 | 67029381-67098808 |
|  |  |  |  |  |  | copy_number_alter  ation | CDH1 | amplification |  | 16 | 68737374-68833499 |
|  |  |  |  |  |  | copy_number_alter  ation | CDK4 | amplification |  | 12 | 57700149-57794361 |
|  |  |  |  |  |  | copy_number_alter  ation | CTCF | amplification |  | 16 | 67610832-67637872 |

|  |  |  |  |  |  | copy_number_alter  ation | JUN | amplification |  | 1 | 58736932-58819812 |
| --- | --- | --- | --- | --- | --- | --- | --- | --- | --- | --- | --- |
|  |  |  |  |  |  | copy_number_alter  ation | MDM2 | amplification |  | 12 | 68760216-68883425 |
|  |  |  |  |  |  | copy_number_alter  ation | MYC | amplification |  | 8 | 127694344-127789205 |
|  |  |  |  |  |  | copy_number_alter  ation | SDHA | amplification |  | 5 | 218322-256441 |
|  |  |  |  |  |  | copy_number_alter  ation | SGK1 | amplification |  | 6 | 134170267-134262148 |
|  |  |  |  |  |  | rearrangement | KIRREL-NTRK1 | fusion |  | 1:01 | 156866970-  156867148:158080236-158080550 |
|  |  |  |  |  |  | small_scale_variant | LTK | S382I | 0.07 (32/491) | 15 | 41508173 |
|  |  |  |  |  |  | small_scale_variant | MSH6 | N1273S | 0.35 (201/579) | 2 | 47806468 |
|  |  |  |  |  |  | small_scale_variant | NOTCH1 | L2234V | 0.49 (250/507) | 9 | 136497039 |
|  |  |  |  |  |  | small_scale_variant | RNF43 | R519Q | 0.48 (323/679) | 17 | 58358220 |
|  |  |  |  |  |  | small_scale_variant | SPEN | R3011Q | 0.51 (337/660) | 1 | 15935272 |
|  |  |  |  |  |  | small_scale_variant | TET2 | G1282S | 0.35 (141/403) | 4 | 105259659 |
|  |  |  |  |  |  | small_scale_variant | TSC2 | A415V | 0.52 (224/432) | 16 | 2061995 |
| Esophagus/Stomach | Esophageal Squamous Cell Carcinoma | 64 | FoundationOne CDx DX1 | 6.3Muts/Mb() | stable | copy_number_alter ation | CDK6 | amplification |  | 7 | 92575965-92875055 |
|  |  |  |  |  |  | copy_number_alter  ation | CDKN2A | loss |  | 9 | 21853213-21998003 |
|  |  |  |  |  |  | copy_number_alter  ation | CDKN2B | loss |  | 9 | 21998749-22101833 |
|  |  |  |  |  |  | copy_number_alter  ation | ERBB2 | amplification |  | 17 | 39700210-39728044 |
|  |  |  |  |  |  | copy_number_alter  ation | GRM3 | amplification |  | 7 | 86765145-86864383 |
|  |  |  |  |  |  | copy_number_alter  ation | HGF | amplification |  | 7 | 81658467-81806298 |
|  |  |  |  |  |  | copy_number_alter  ation | MET | amplification |  | 7 | 116657451-116844231 |
|  |  |  |  |  |  | copy_number_alter  ation | MTAP | loss |  | 9 | 21802700-21862059 |
|  |  |  |  |  |  | interaction | PIK3CA,  ERBB2 | H1047R,  Amplification | 0.01 (14/1565), | 17 | 39700210-39728044 |
|  |  |  |  |  |  | interaction | PIK3CA,  ERBB2 | H1047R,  Amplification | 0.01 (14/1565), | 3 | 179234297 |
|  |  |  |  |  |  | rearrangement | NTRK2-FGF7 | fusion |  | 9:15 | 84742101-84742332:49461342-  49461766 |
|  |  |  |  |  |  | small_scale_variant | CBFB | Y96C | 0.12 (85/686) | 16 | 67066686 |
|  |  |  |  |  |  | small_scale_variant | EP300 | G322A | 0.60 (425/707) | 22 | 41127545 |
|  |  |  |  |  |  | small_scale_variant | EP300 | V456I | 0.56 (437/783) | 22 | 41131471 |
|  |  |  |  |  |  | small_scale_variant | EPHB1 | V391I | 0.24 (258/1090) | 3 | 135132923 |
|  |  |  |  |  |  | small_scale_variant | FANCA | S849fs*40 | 0.26 (156/598) | 16 | 89767195 |
|  |  |  |  |  |  | small_scale_variant | FANCL | Y102C | 0.25 (259/1050) | 2 | 58222011 |
|  |  |  |  |  |  | small_scale_variant | GATA3 | A396T | 0.47 (391/835) | 10 | 8073874 |
|  |  |  |  |  |  | small_scale_variant | KMT2D | V4946fs*49 | 0.25 (258/1022) | 12 | 49027128 |
|  |  |  |  |  |  | small_scale_variant | MAF | A251T | 0.21 (46/220) | 16 | 79599152 |

|  |  |  |  |  | small_scale_variant | NFE2L2 | E82D | 0.28 (335/1176) | 2 | 177234071 |
| --- | --- | --- | --- | --- | --- | --- | --- | --- | --- | --- |
|  |  |  |  |  | small_scale_variant | NTRK2 | L584F | 0.26 (180/693) | 9 | 84934280 |
|  |  |  |  |  | small_scale_variant | PIK3CA | H1047R | 0.01 (14/1565) | 3 | 179234297 |
|  |  |  |  |  | small_scale_variant | PTCH1 | R1303C | 0.22 (140/632) | 9 | 95447349 |
|  |  |  |  |  | small_scale_variant | PTPRO | A808D | 0.25 (227/894) | 12 | 15549212 |
|  |  |  |  |  | small_scale_variant | RAF1 | c.207+1G>T | 0.38 (246/645) | 3 | 12618514 |
|  |  |  |  |  | small_scale_variant | TP53 | C176F | 0.52 (477/926) | 17 | 7675085 |
| Lung | 78 | FoundationOne CDx DX1 | 11.35Muts/Mb() | stable | copy_number_alter ation | BCL6 | amplification |  | 3 | 187721382-187733693 |
|  |  |  |  |  | copy_number_alter  ation | FAS | loss |  | 10 | 88990826-89015850 |
|  |  |  |  |  | copy_number_alter  ation | FGF12 | amplification |  | 3 | 192102988-192769418 |
|  |  |  |  |  | copy_number_alter  ation | IRF4 | amplification |  | 6 | 393152-407598 |
|  |  |  |  |  | copy_number_alter  ation | KLHL6 | amplification |  | 3 | 183491926-183555653 |
|  |  |  |  |  | copy_number_alter  ation | MAP3K13 | amplification |  | 3 | 185285559-185482470 |
|  |  |  |  |  | copy_number_alter  ation | PTEN | loss |  | 10 | 87788572-88035896 |
|  |  |  |  |  | copy_number_alter  ation | PTEN | loss |  | 10 | 87788572-88035896 |
|  |  |  |  |  | copy_number_alter  ation | PTEN | loss |  | 10 | 87788572-88035896 |
|  |  |  |  |  | copy_number_alter  ation | PTEN | loss |  | 10 | 87788572-88035896 |
|  |  |  |  |  | copy_number_alter  ation | PTEN | loss |  | 10 | 87788572-88035896 |
|  |  |  |  |  | copy_number_alter  ation | PTEN | loss |  | 10 | 87788572-88035896 |
|  |  |  |  |  | copy_number_alter  ation | PTEN | loss |  | 10 | 87788572-88035896 |
|  |  |  |  |  | copy_number_alter  ation | SOX2 | amplification |  | 3 | 181665448-181758377 |
|  |  |  |  |  | rearrangement | THEM5-  NTRK1 | fusion |  | 1:01 | 156841962-  156842276:151849383-151849651 |
|  |  |  |  |  | small_scale_variant | BRCA2 | E357Q | 0.06 (69/1202) | 13 | 32332547 |
|  |  |  |  |  | small_scale_variant | CD22 | P671R | 0.29 (304/1060) | 19 | 35341942 |
|  |  |  |  |  | small_scale_variant | CSF3R | G182R | 0.36 (582/1609) | 1 | 36473564 |
|  |  |  |  |  | small_scale_variant | GRM3 | D279Y | 0.59 (608/1031) | 7 | 86786627 |
|  |  |  |  |  | small_scale_variant | GSK3B | R332H | 0.08 (92/1195) | 3 | 119863559 |
|  |  |  |  |  | small_scale_variant | KIT | A895P | 0.27 (262/959) | 4 | 54736807 |
|  |  |  |  |  | small_scale_variant | LYN | T454N | 0.35 (232/661) | 8 | 56009932 |
|  |  |  |  |  | small_scale_variant | MUTYH | c.892-2A>G | 0.77 (1256/1641) | 1 | 45332088 |
|  |  |  |  |  | small_scale_variant | NTRK2 | G357W | 0.46 (701/1510) | 9 | 84727869 |
|  |  |  |  |  | small_scale_variant | PDCD1 | F219L | 0.07 (65/893) | 2 | 241851270 |
|  |  |  |  |  | small_scale_variant | PIK3C2G | N1088T | 0.28 (237/844) | 12 | 18538218 |

|  |  |  |  |  |  | small_scale_variant | PMS2 | I787L | 0.20 (13/65) | 7 | 5977674 |
| --- | --- | --- | --- | --- | --- | --- | --- | --- | --- | --- | --- |
|  |  |  |  |  |  | small_scale_variant | POLE | T41M | 0.45 (283/633) | 12 | 132681220 |
|  |  |  |  |  |  | small_scale_variant | RB1 | Y173* | 0.85 (865/1018) | 13 | 48347843 |
|  |  |  |  |  |  | small_scale_variant | RET | I669V | 0.78 (582/750) | 10 | 43114605 |
|  |  |  |  |  |  | small_scale_variant | RICTOR | L1537R | 0.37 (421/1153) | 5 | 38945514 |
|  |  |  |  |  |  | small_scale_variant | TET2 | R544* | 0.04 (31/816) | 4 | 105235572 |
|  |  |  |  |  |  | small_scale_variant | TP53 | T155P | 0.85 (860/1007) | 17 | 7675149 |
| Thyroid | Thyroid | 47 | FoundationOne CDx DX1 | 3.78Muts/Mb() | cannot be determin ed | rearrangement | NTRK1-NPTXR | fusion |  | 1:22 | 156873764-156874055:38837513-  38837751 |
|  |  |  |  |  |  | rearrangement | TPM3-NTRK1 | fusion |  | 1:01 | 156874100-  156874417:154158664-154158871 |
|  |  |  |  |  |  | small_scale_variant | AR | D354E | 1.00 (347/348) | X | 67546208 |
|  |  |  |  |  |  | small_scale_variant | CARD11 | V171M | 0.51 (419/817) | 7 | 2944385 |
|  |  |  |  |  |  | small_scale_variant | CSF1R | N968del | 0.45 (250/552) | 5 | 150054083 |
|  |  |  |  |  |  | small_scale_variant | NTRK1 | P366L | 0.33 (183/557) | 1 | 156873879 |
|  |  |  |  |  |  | small_scale_variant | PIK3R1 | L356S | 0.50 (737/1471) | 5 | 68293148 |
|  |  |  |  |  |  | small_scale_variant | RNF43 | E170K | 0.48 (306/633) | 17 | 58363349 |
|  |  |  |  |  |  | small_scale_variant | TERT | c.-79-45C>T | 0.40 (28/70) | 5 | 1295113 |
| Soft Tissue | Soft Tissue | 45 | FoundationOne CDx DX1 | 5.04Muts/Mb() | stable | copy_number_alter ation | CDKN2A | loss |  | 9 | 21853213-21998003 |
|  |  |  |  |  |  | copy_number_alter  ation | CDKN2B | loss |  | 9 | 21998749-22101833 |
|  |  |  |  |  |  | copy_number_alter  ation | MTAP | loss |  | 9 | 21802700-21862059 |
|  |  |  |  |  |  | rearrangement | ETV6-TERF1 | fusion |  | 12:08 | 11882433-11882656:73038466-  73038691 |
|  |  |  |  |  |  | rearrangement | LMNA-NTRK1 | fusion |  | 1:01 | 156874823-  156875047:156133718-156134088 |
|  |  |  |  |  |  | small_scale_variant | AMER1 | N852K | 0.53 (519/981) | X | 64190731 |
|  |  |  |  |  |  | small_scale_variant | BRIP1 | G481D | 0.71 (1065/1492) | 17 | 61793628 |
|  |  |  |  |  |  | small_scale_variant | CUL4A | K461E | 0.28 (236/833) | 13 | 113253124 |
|  |  |  |  |  |  | small_scale_variant | IKBKE | G660E | 0.64 (589/926) | 1 | 206493312 |
|  |  |  |  |  |  | small_scale_variant | NF1 | L1377P | 0.50 (505/1012) | 17 | 31252957 |
| Head and Neck | Head and Neck | 55 | FoundationOne CDx DX1 | 0.0Muts/Mb() | stable | rearrangement | ETV6-NTRK3 | fusion |  | 15:12 | 87951485-87951485:11877093-  11877093 |
|  |  |  |  |  |  | small_scale_variant | CD22 | V677I | 0.48 (440/915) | 19 | 35341959 |
|  |  |  |  |  |  | small_scale_variant | DIS3 | T869R | 0.58 (516/883) | 13 | 72760626 |
|  |  |  |  |  |  | small_scale_variant | IRF2 | R253Q | 0.50 (497/1000) | 4 | 184389050 |
|  |  |  |  |  |  | small_scale_variant | NF2 | V72L | 0.53 (496/943) | 22 | 29636850 |
|  |  |  |  |  |  | small_scale_variant | NOTCH3 | R75P | 0.53 (361/687) | 19 | 15192493 |
|  |  |  |  |  |  | small_scale_variant | SDHC | F18L | 0.48 (335/694) | 1 | 161323647 |
| Soft Tissue | Sarcoma, NOS | 19 | FoundationOne Liquid CDx AB1 | 3Muts/Mb() | high not detected | copy_number_alter ation | AKT3 | amplification |  | 1 | 243455698-243893220 |

| copy_number_alter  ation | EGFR | amplification |  | 7 | 54970060-55255535 |
| --- | --- | --- | --- | --- | --- |
| copy_number_alter  ation | EPHA3 | amplification |  | 3 | 89107730-89479511 |
| copy_number_alter  ation | IKZF1 | amplification |  | 7 | 50319016-50400627 |
| copy_number_alter  ation | KIT | amplification |  | 4 | 54641380-54784875 |
| copy_number_alter  ation | PDGFRA | amplification |  | 4 | 54213247-54307896 |
| copy_number_alter  ation | RNF43 | amplification |  | 17 | 58354899-58415577 |
| copy_number_alter  ation | TGFBR2 | amplification |  | 3 | 30623174-30691599 |
| rearrangement | BRCA1 | truncation |  | 17:19 | 43081435-43081435:44875237-  44875237 |
| rearrangement | KIAA1549-  BRAF | fusion |  | 7:07 | 140787710-  140787710:138838048-138838048 |
| rearrangement | NTRK1 | rearrangements |  | 1:01 | 156874677-  156874677:157054101-157054101 |
| rearrangement | PPP2R1A | rearrangements |  | 19:17 | 52219846-52219846:73846717-  73846717 |
| rearrangement | PPP2R1A | rearrangements |  | 19:19 | 52221276-52221276:47120212-  47120212 |
| rearrangement | SMO | rearrangements |  | 7:07 | 129210498-  129210498:129211152-129211152 |
| rearrangement | SMO | rearrangements |  | 7:07 | 129211986-  129211986:149092536-149092536 |
| rearrangement | TPM3-NTRK1 | fusion |  | 1:01 | 156873300-  156873300:154159211-154159211 |
| small_scale_variant | AKT1 | E17K | 0 | 14 | 104780214 |
| small_scale_variant | ARID1A | N957S | 0.52 | 1 | 26766358 |
| small_scale_variant | ERBB3 | C259R | 0 | 12 | 56088063 |
| small_scale_variant | FANCC | A325T | 0.46 | 9 | 95125109 |
| small_scale_variant | FGFR1 | G555V | 0 | 8 | 38416060 |
| small_scale_variant | LTK | G212_G213insGG G | 0.51 | 15 | 41511835 |
| small_scale_variant | MYC | K304I | 0 | 8 | 127740504 |
| small_scale_variant | NF1 | E2195fs*46 | 0 | 17 | 31337524 |
| small_scale_variant | NRAS | Q61R | 0 | 1 | 114713908 |
| small_scale_variant | PDGFRB | L595Q | 0.01 | 5 | 150125468 |
| small_scale_variant | PDGFRB | N666K | 0.04 | 5 | 150124275 |
| small_scale_variant | PDGFRB | N666S | 0 | 5 | 150124276 |
| small_scale_variant | PDGFRB | R565_D575del | 0 | 5 | 150125526 |
| small_scale_variant | PDGFRB | V568E | 0.01 | 5 | 150125549 |
| small_scale_variant | PDGFRB | Y562C | 0.09 | 5 | 150125567 |
| small_scale_variant | SMO | P505R | 0.01 | 7 | 129210410 |
| small_scale_variant | STK11 | F354L | 0.44 | 19 | 1223126 |
| small_scale_variant | TP53 | R273H | 0.01 | 17 | 7673802 |

|  |  |  |  |  |  | small_scale_variant | TP53 | R342* | 0.31 | 17 | 7670685 |
| --- | --- | --- | --- | --- | --- | --- | --- | --- | --- | --- | --- |
|  |  |  |  |  |  | small_scale_variant | TSC2 | G661S | 0.41 | 16 | 2071818 |
| Soft Tissue | Infantile Fibrosarcoma | 0 | FoundationOne CDx DX1 | 0Muts/Mb() | stable | rearrangement | ETV6-NTRK3 | fusion |  | 15:12 | 87955179-87955179:11884039-  11884039 |
|  |  |  |  |  |  | small_scale_variant | CALR | P205S | 0.51 | 19 | 12940363 |
|  |  |  |  |  |  | small_scale_variant | EP300 | P1986L | 0.51 | 22 | 41177668 |
|  |  |  |  |  |  | small_scale_variant | MET | T230M | 0.5 | 7 | 116699773 |
| Head and Neck |  | 38 | FoundationOne CDx DX1 | 2.52Muts/Mb() | stable | rearrangement | ETV6-NTRK3 | fusion |  | 15:12 | 88017583-88017815:11881911-  11882207 |
|  |  |  |  |  |  | small_scale_variant | BRCA2 | H1467Y | 0.48 (455/948) | 13 | 32338754 |
|  |  |  |  |  |  | small_scale_variant | CD70 | T118M | 0.49 (530/1073) | 19 | 6586249 |
|  |  |  |  |  |  | small_scale_variant | LTK | G212_G213insGG G | 0.37 (315/851) | 15 | 41511835 |
|  |  |  |  |  |  | small_scale_variant | LTK | R203C | 0.32 (247/763) | 15 | 41511867 |
|  |  |  |  |  |  | small_scale_variant | SETD2 | S1442fs*12 | 0.28 (377/1348) | 3 | 47120312 |
|  |  |  |  |  |  | small_scale_variant | TSC2 | R1159W | 0.39 (468/1208) | 16 | 2080242 |
| Thyroid | Papillary Thyroid Cancer | 82 | FoundationOne CDx DX2 | 0Muts/Mb() | stable | rearrangement | TPM3-NTRK1 | fusion |  | 1:01 | 156875122-  156875661:154157828-154158532 |
|  |  |  |  |  |  | small_scale_variant | NOTCH3 | G1134R | 0.4872 | 19 | 15179424 |
|  |  |  |  |  |  | small_scale_variant | PIK3C2B | P172S | 0.4882 | 1 | 204469289 |
| Prostate | Prostate Adenocarcinoma | 85 | FoundationOne CDx DX2 | 2Muts/Mb() | stable | copy_number_alter ation | AR | amplification |  | X | 67210788-67760904 |
|  |  |  |  |  |  | copy_number_alter  ation | RAD21 | amplification |  | 8 | 116847499-116866729 |
|  |  |  |  |  |  | rearrangement | NSD3 | rearrangements |  | 8:08 | 38347557-38347557:29753778-  29753778 |
|  |  |  |  |  |  | rearrangement | SYK-NTRK2 | fusion |  | 9:09 | 84700100-84700100:90878905-  90878905 |
|  |  |  |  |  |  | small_scale_variant | KMT2D | 3936_3939dupQ  QQQ | 0.4905 | 12 | 49032897 |
|  |  |  |  |  |  | small_scale_variant | STAG2 | H138P | 0.444 | X | 124042596 |
|  |  |  |  |  |  | small_scale_variant | TET2 | P409S | 0.4623 | 4 | 105235167 |
| Prostate | Prostate Adenocarcinoma | 67 | FoundationOne Liquid CDx AB1 | 1Muts/Mb() | high not detected | rearrangement | NTRK1 | rearrangements |  | 1:01 | 156874803-  156874803:161182513-161182513 |
|  |  |  |  |  |  | rearrangement | PBX1-NTRK1 | fusion |  | 1:01 | 156874883-  156874883:164732610-164732610 |
|  |  |  |  |  |  | rearrangement | TMPRSS2-ERG | fusion |  | 21:21 | 41497353-41497353:38496868-  38496868 |
|  |  |  |  |  |  | small_scale_variant | DNMT3A | R604fs*47 | 0.0023 | 2 | 25244195 |
|  |  |  |  |  |  | small_scale_variant | DNMT3A | c.1668-2A>G | 0.0072 | 2 | 25244340 |
|  |  |  |  |  |  | small_scale_variant | EGFR | T211A | 0.0017 | 7 | 55152548 |
|  |  |  |  |  |  | small_scale_variant | EP300 | S442A | 0.476 | 22 | 41131429 |
|  |  |  |  |  |  | small_scale_variant | GNAS | C174Y | 0.0046 | 20 | 58905471 |
|  |  |  |  |  |  | small_scale_variant | GNAS | R201C | 0.0538 | 20 | 58909365 |
|  |  |  |  |  |  | small_scale_variant | NOTCH3 | P1633L | 0.4801 | 19 | 15170547 |
|  |  |  |  |  |  | small_scale_variant | TET2 | S825fs*1 | 0.0108 | 4 | 105236415 |

|  |  |  |  |  |  | small_scale_variant | TP53 | c.375+5G>T | 0.0039 | 17 | 7675989 |
| --- | --- | --- | --- | --- | --- | --- | --- | --- | --- | --- | --- |
| Breast | Breast Invasive Ductal Carcinoma | 34 | FoundationOne CDx DX2 | 1Muts/Mb() | stable | copy_number_alter ation | CTNNA1 | loss |  | 5 | 138781912-138827718 |
|  |  |  |  |  |  | rearrangement | ETV6 | rearrangements |  | 12:12 | 11875470-11875637:11939182-  11939329 |
|  |  |  |  |  |  | rearrangement | ETV6-NTRK3 | fusion |  | 15:12 | 88087328-88087328:11875430-  11875430 |
|  |  |  |  |  |  | rearrangement | TIPARP | rearrangements |  | 3:03 | 156694026-  156694213:156686110-156686346 |
|  |  |  |  |  |  | small_scale_variant | APC | R2673G | 0.32 | 5 | 112843611 |
|  |  |  |  |  |  | small_scale_variant | BARD1 | R749T | 0.47 | 2 | 214728764 |
|  |  |  |  |  |  | small_scale_variant | BRIP1 | R814C | 0.32 | 17 | 61716003 |
|  |  |  |  |  |  | small_scale_variant | CTNNA1 | N257S | 0.49 | 5 | 138824711 |
|  |  |  |  |  |  | small_scale_variant | FANCA | A737V | 0.49 | 16 | 89770576 |
|  |  |  |  |  |  | small_scale_variant | FBXW7 | Q277_F280del | 0.28 | 4 | 152337822 |
|  |  |  |  |  |  | small_scale_variant | IDH1 | R20Q | 0.5 | 2 | 208251493 |
|  |  |  |  |  |  | small_scale_variant | JAK2 | W659R | 0.65 | 9 | 5077563 |
|  |  |  |  |  |  | small_scale_variant | MSH2 | P5Q | 0.37 | 2 | 47403205 |
|  |  |  |  |  |  | small_scale_variant | NKX2-1 | G322S | 0.27 | 14 | 36517430 |
|  |  |  |  |  |  | small_scale_variant | TP53 | L194R | 0.39 | 17 | 7674950 |
| Soft Tissue | Gastrointestinal Stromal Tumor | 66 | FoundationOne CDx DX2 | 5Muts/Mb() | stable | rearrangement | ETV6-NTRK3 | fusion |  | 15:12 | 87978753-87978753:11872199-  11872199 |
|  |  |  |  |  |  | small_scale_variant | ATM | T935R | 0.47 | 11 | 108268575 |
|  |  |  |  |  |  | small_scale_variant | BRCA2 | K322Q | 0.49 | 13 | 32332442 |
|  |  |  |  |  |  | small_scale_variant | BRD4 | P781S | 0.63 | 19 | 15244471 |
|  |  |  |  |  |  | small_scale_variant | CIC | N702S | 0.51 | 19 | 42290873 |
|  |  |  |  |  |  | small_scale_variant | FANCG | V125dup | 0.5 | 9 | 35078275 |
|  |  |  |  |  |  | small_scale_variant | NOTCH3 | A1450T | 0.49 | 19 | 15177580 |
|  |  |  |  |  |  | small_scale_variant | NOTCH3 | S832N | 0.49 | 19 | 15184366 |
|  |  |  |  |  |  | small_scale_variant | SETD2 | R400Q | 0.5 | 3 | 47123437 |
|  |  |  |  |  |  | small_scale_variant | TP53 | E286K | 0.01 | 17 | 7673764 |
| Head and Neck | Mammary Analogue Secretory Carcinoma of Salivary Gland Origin | 60 | FoundationOne CDx DX2 | 2Muts/Mb() | stable | copy_number_alter ation | CDKN2A | loss |  | 9 | 21970901-21998003 |
|  |  |  |  |  |  | rearrangement | ETV6-NTRK3 | fusion |  | 15:12 | 87961967-87961967:11876084-  11876084 |
|  |  |  |  |  |  | small_scale_variant | ATR | D1915G | 0.43 | 3 | 142496515 |
|  |  |  |  |  |  | small_scale_variant | BRCA2 | K322Q | 0.52 | 13 | 32332442 |
|  |  |  |  |  |  | small_scale_variant | EPHA3 | R136Q | 0.43 | 3 | 89210113 |
|  |  |  |  |  |  | small_scale_variant | GATA3 | P425L | 0.47 | 10 | 8073962 |
|  |  |  |  |  |  | small_scale_variant | MAP2K2 | V400M | 0.5 | 19 | 4090603 |
|  |  |  |  |  |  | small_scale_variant | MSH2 | H839R | 0.52 | 2 | 47480753 |
|  |  |  |  |  |  | small_scale_variant | PALB2 | Q460R | 0.38 | 16 | 23635167 |

|  |  |  |  |  |  | small_scale_variant | STK11 | F354L | 0.49 | 19 | 1223126 |
| --- | --- | --- | --- | --- | --- | --- | --- | --- | --- | --- | --- |
| Prostate | Prostate Adenocarcinoma | 78 | FoundationOne Liquid CDx AB1 | 20.23Muts/Mb(hi gh) | high not detected | copy_number_alter ation | AR | amplification |  | X | 67210788-67760904 |
|  |  |  |  |  |  | copy_number_alter  ation | CARD11 | amplification |  | 7 | 2906637-2958567 |
|  |  |  |  |  |  | copy_number_alter  ation | PTEN | loss |  | 10 | 87960893-87965472 |
|  |  |  |  |  |  | rearrangement | NTRK1-LPPR1 | fusion |  | 1:09 | 156872451-  156872451:101042833-101042833 |
|  |  |  |  |  |  | rearrangement | TMPRSS2-ERG | fusion |  | 21:21 | 41499245-41499245:38650957-  38650957 |
|  |  |  |  |  |  | small_scale_variant | AR | I915M | 0 | X | 67723823 |
|  |  |  |  |  |  | small_scale_variant | AR | S901Y | 0 | X | 67723780 |
|  |  |  |  |  |  | small_scale_variant | BRCA2 | K178fs*7 | 0.01 | 13 | 32326515 |
|  |  |  |  |  |  | small_scale_variant | BRCA2 | V3078I | 0 | 13 | 32380121 |
|  |  |  |  |  |  | small_scale_variant | CARD11 | G924W | 0.09 | 7 | 2915306 |
|  |  |  |  |  |  | small_scale_variant | CHEK1 | D351H | 0.13 | 11 | 125644218 |
|  |  |  |  |  |  | small_scale_variant | CHEK2 | Y337D | 0 | 22 | 28696987 |
|  |  |  |  |  |  | small_scale_variant | CSF3R | R853L | 0.17 | 1 | 36466391 |
|  |  |  |  |  |  | small_scale_variant | ERBB4 | R103C | 0.33 | 2 | 211947544 |
|  |  |  |  |  |  | small_scale_variant | GNAS | K638N | 0 | 20 | 58855179 |
|  |  |  |  |  |  | small_scale_variant | ID3 | D42E | 0.26 | 1 | 23559301 |
|  |  |  |  |  |  | small_scale_variant | JAK2 | D185Y | 0.44 | 9 | 5050770 |
|  |  |  |  |  |  | small_scale_variant | JAK2 | c.1993-  46_2000del54 | 0.04 | 9 | 5078259 |
|  |  |  |  |  |  | small_scale_variant | MERTK | A910S | 0.02 | 2 | 112028592 |
|  |  |  |  |  |  | small_scale_variant | MERTK | Q233R | 0.54 | 2 | 111947508 |
|  |  |  |  |  |  | small_scale_variant | MET | D153V | 0.02 | 7 | 116699542 |
|  |  |  |  |  |  | small_scale_variant | NTRK1 | c.360-1G>T | 0.02 | 1 | 156866909 |
|  |  |  |  |  |  | small_scale_variant | RET | A1019V | 0.02 | 10 | 43126591 |
|  |  |  |  |  |  | small_scale_variant | STK11 | F354L | 0.71 | 19 | 1223126 |
|  |  |  |  |  |  | small_scale_variant | TNFRSF14 | Q148* | 0 | 1 | 2559960 |
|  |  |  |  |  |  | small_scale_variant | TP53 | E258K | 0.45 | 17 | 7674191 |
|  |  |  |  |  |  | small_scale_variant | VEGFA | E278D | 0.28 | 6 | 43777644 |
| Skin | Skin | 51 | OncoGuideTM NCC Oncopanel system | 0.00Muts/Mb() | 1.96%(sta ble) | rearrangement | ETV6-NTRK3 | fusion | 0.39 | 12:15 | 11883254-11883254:87955038-  87955038 |
|  |  |  |  |  |  | small_scale_variant | ATM | A220V | 0.47 (116/248) | 11 | 108244115 |
|  |  |  |  |  |  | small_scale_variant | BAP1 | T351A | 0.47 (191/411) | 3 | 52405175 |
| Other | Undifferentiated Malignant Neoplasm | 0 | OncoGuideTM NCC Oncopanel system | 0.00Muts/Mb() | 0.46%(sta ble) | rearrangement | TPM3-NTRK1 | fusion | 0.08 | 1:01 | 154158150-  154158152:156874069-156874071 |
|  |  |  |  |  |  | small_scale_variant | FGFR1 | R767Q | 0.51 (941/1856) | 8 | 38414009 |

|  |  |  |  |  |  | small_scale_variant | NOTCH1 | S2471L | 0.49 (373/763) | 9 | 136496327 |
| --- | --- | --- | --- | --- | --- | --- | --- | --- | --- | --- | --- |
| Head and Neck | Mammary Analogue Secretory Carcinoma of Salivary Gland Origin | 33 | FoundationOne CDx DX1 | 0.0Muts/Mb() | cannot be determin ed | rearrangement | ETV6-NTRK3 | fusion |  | 15:12 | 87973411-87973411:11875248-  11875248 |
|  |  |  |  |  |  | small_scale_variant | CEBPA | I62V | 0.48 (235/491) | 19 | 33302231 |
|  |  |  |  |  |  | small_scale_variant | IKBKE | R500Q | 0.49 (392/802) | 1 | 206485068 |
|  |  |  |  |  |  | small_scale_variant | LTK | G212_G213insGG G | 0.39 (48/122) | 15 | 41511835 |
|  |  |  |  |  |  | small_scale_variant | MRE11 | R87W | 0.52 (367/701) | 11 | 94485979 |
|  |  |  |  |  |  | small_scale_variant | SETD2 | S355P | 0.50 (505/1007) | 3 | 47123573 |
|  |  |  |  |  |  | small_scale_variant | TSC1 | T417I | 0.49 (426/877) | 9 | 132910584 |
| Adrenal Gland | Adrenocortical Carcinoma | 50 | OncoGuideTM NCC Oncopanel system | 178.3Muts/Mb() |  | rearrangement | NRG1-NTRK2 | fusion |  | 8:09 | 32420183-32420189:84824723-  84824729 |
|  |  |  |  |  |  | small_scale_variant | BRCA2 | I1929V | 0.57 (114/200) | 13 | 32340140 |
|  |  |  |  |  |  | small_scale_variant | ARID1A | S1090R | 0.07 (6/87) | 1 | 26771190 |
|  |  |  |  |  |  | small_scale_variant | ARID1A | c.2989-1G>T | 0.06 (5/84) | 1 | 26767789 |
|  |  |  |  |  |  | small_scale_variant | ARID2 | V1177L | 0.06 (5/80) | 12 | 45851652 |
|  |  |  |  |  |  | small_scale_variant | ATM | P1680R | 0.07 (5/76) | 11 | 108299747 |
|  |  |  |  |  |  | small_scale_variant | ATM | R2227S | 0.07 (5/70) | 11 | 108325416 |
|  |  |  |  |  |  | small_scale_variant | ATM | V1729F | 0.05 (5/95) | 11 | 108301655 |
|  |  |  |  |  |  | small_scale_variant | BARD1 | P546R | 0.05 (5/97) | 2 | 214752487 |
|  |  |  |  |  |  | small_scale_variant | BARD1 | T81N | 0.09 (5/56) | 2 | 214792419 |
|  |  |  |  |  |  | small_scale_variant | BRAF | Q201K | 0.05 (5/91) | 7 | 140808899 |
|  |  |  |  |  |  | small_scale_variant | BRCA1 | S1577P | 0.12 (8/65) | 17 | 43071185 |
|  |  |  |  |  |  | small_scale_variant | BRCA2 | V1076L | 0.07 (6/91) | 13 | 32337581 |
|  |  |  |  |  |  | small_scale_variant | CUL3 | G143* | 0.09 (7/82) | 2 | 224514724 |
|  |  |  |  |  |  | small_scale_variant | ERBB2 | I655V | 0.10 (16/160) | 17 | 39723335 |
|  |  |  |  |  |  | small_scale_variant | ERBB3 | A232V | 0.06 (12/188) | 12 | 56087876 |
|  |  |  |  |  |  | small_scale_variant | ERBB3 | N293K | 0.06 (6/93) | 12 | 56088547 |
|  |  |  |  |  |  | small_scale_variant | ERBB3 | P860H | 0.05 (6/116) | 12 | 56097903 |
|  |  |  |  |  |  | small_scale_variant | ERBB4 | G336C | 0.08 (5/62) | 2 | 211712168 |
|  |  |  |  |  |  | small_scale_variant | EZH2 | E740* | 0.07 (5/68) | 7 | 148807669 |
|  |  |  |  |  |  | small_scale_variant | EZH2 | M41I | 0.07 (5/74) | 7 | 148846593 |
|  |  |  |  |  |  | small_scale_variant | FBXW7 | D173E | 0.07 (6/84) | 4 | 152350107 |
|  |  |  |  |  |  | small_scale_variant | FBXW7 | D510H | 0.07 (5/76) | 4 | 152326122 |
|  |  |  |  |  |  | small_scale_variant | FGFR4 | A229T | 0.05 (27/525) | 5 | 177091766 |
|  |  |  |  |  |  | small_scale_variant | IGF1R | A746P | 0.05 (6/118) | 15 | 98922182 |
|  |  |  |  |  |  | small_scale_variant | JAK1 | D1042H | 0.06 (5/84) | 1 | 64837948 |

|  |  |  |  |  |  | small_scale_variant | JAK2 | V486L | 0.06 (5/84) | 9 | 5069151 |
| --- | --- | --- | --- | --- | --- | --- | --- | --- | --- | --- | --- |
|  |  |  |  |  |  | small_scale_variant | KDM6A | E1064* | 0.05 (6/117) | X | 45082621 |
|  |  |  |  |  |  | small_scale_variant | MET | W1320C | 0.08 (6/79) | 7 | 116795911 |
|  |  |  |  |  |  | small_scale_variant | MLH1 | D214H | 0.08 (5/62) | 3 | 37012062 |
|  |  |  |  |  |  | small_scale_variant | NF1 | D1058Y | 0.06 (5/82) | 17 | 31230900 |
|  |  |  |  |  |  | small_scale_variant | NRG1 | G616V | 0.07 (16/215) | 8 | 32764326 |
|  |  |  |  |  |  | small_scale_variant | NT5C2 | L395F | 0.07 (6/85) | 10 | 103091590 |
|  |  |  |  |  |  | small_scale_variant | PALB2 | S980R | 0.06 (7/123) | 16 | 23623025 |
|  |  |  |  |  |  | small_scale_variant | PALB2 | T166N | 0.09 (7/80) | 16 | 23636049 |
|  |  |  |  |  |  | small_scale_variant | PBRM1 | R921L | 0.07 (5/76) | 3 | 52603538 |
|  |  |  |  |  |  | small_scale_variant | PBRM1 | S691Y | 0.05 (5/92) | 3 | 52609808 |
|  |  |  |  |  |  | small_scale_variant | PBRM1 | c.385C>G | 0.07 (6/82) | 3 | 52662276 |
|  |  |  |  |  |  | small_scale_variant | PDGFRA | A93T | 0.15 (9/61) | 4 | 54261322 |
|  |  |  |  |  |  | small_scale_variant | PRKCI | L84F | 0.06 (5/86) | 3 | 170259997 |
|  |  |  |  |  |  | small_scale_variant | ROS1 | D2213N | 0.08 (5/65) | 6 | 117301070 |
|  |  |  |  |  |  | small_scale_variant | ROS1 | S2229C | 0.09 (6/66) | 6 | 117301021 |
|  |  |  |  |  |  | small_scale_variant | SETBP1 | V231F | 0.07 (7/99) | 18 | 44950031 |
|  |  |  |  |  |  | small_scale_variant | SMAD4 | F310L | 0.06 (6/102) | 18 | 51059891 |
|  |  |  |  |  |  | small_scale_variant | TSC1 | S1164I | 0.06 (7/126) | 9 | 132896239 |
| Soft Tissue | Sarcoma, NOS | 0 | FoundationOne CDx DX1 | 0.0Muts/Mb() | cannot be determin ed | rearrangement | ETV6-NTRK3 | fusion |  | 15:12 | 87984019-87984019:11881781-  11881781 |
|  |  |  |  |  |  | small_scale_variant | FGFR3 | L164V | 0.49 (186/383) | 4 | 1801411 |
|  |  |  |  |  |  | small_scale_variant | JAK2 | V392M | 0.52 (392/753) | 9 | 5065000 |
|  |  |  |  |  |  | small_scale_variant | MUTYH | c.892-2A>G | 0.50 (300/603) | 1 | 45332088 |
|  |  |  |  |  |  | small_scale_variant | RAD51D | R275W | 0.50 (266/527) | 17 | 35101281 |
|  |  |  |  |  |  | small_scale_variant | SMO | K575M | 0.44 (235/532) | 7 | 129211036 |
| Soft Tissue | Dedifferentiated Liposarcoma | 74 | FoundationOne CDx DX1 | 1Muts/Mb() | stable | copy_number_alter ation | CDK4 | amplification |  | 12 | 57715890-57794361 |
|  |  |  |  |  |  | copy_number_alter  ation | CDK4 | amplification |  | 12 | 57715890-57794361 |
|  |  |  |  |  |  | copy_number_alter  ation | ESR1 | amplification |  | 6 | 151759521-152148205 |
|  |  |  |  |  |  | copy_number_alter  ation | MDM2 | amplification |  | 12 | 68760216-68883425 |
|  |  |  |  |  |  | copy_number_alter  ation | NTRK1 | loss |  | 1 | 156842075-156873959 |
|  |  |  |  |  |  | copy_number_alter  ation | TNFAIP3 | amplification |  | 6 | 137799787-137881319 |
|  |  |  |  |  |  | rearrangement | MEF2D-  NTRK1 | fusion |  | 1:01 | 156874096-  156874465:156478781-156479045 |
|  |  |  |  |  |  | small_scale_variant | EP300 | K1358R | 0.44 | 22 | 41168768 |
|  |  |  |  |  |  | small_scale_variant | NOTCH3 | A1450T | 0.51 | 19 | 15177580 |

|  |  |  |  |  |  | small_scale_variant | STAT3 | T341I | 0.33 | 17 | 42333700 |
| --- | --- | --- | --- | --- | --- | --- | --- | --- | --- | --- | --- |
|  |  |  |  |  |  | small_scale_variant | TGFBR2 | S46R | 0.49 | 3 | 30623240 |
| Peripheral Nervous System | Malignant Peripheral Nerve Sheath Tumor | 20 | FoundationOne CDx DX1 | 0Muts/Mb() | stable | copy_number_alter ation | CDKN2A | loss |  | 9 | 21954945-21998003 |
|  |  |  |  |  |  | copy_number_alter  ation | CDKN2B | loss |  | 9 | 21998749-22064596 |
|  |  |  |  |  |  | rearrangement | LMNA-NTRK1 | fusion |  | 1:01 | 156874801-  156875048:156133526-156133778 |
|  |  |  |  |  |  | small_scale_variant | BARD1 | L123W | 0.49 | 2 | 214781506 |
|  |  |  |  |  |  | small_scale_variant | GABRA6 | K351N | 0.51 | 5 | 161692167 |
|  |  |  |  |  |  | small_scale_variant | GNAS | E132K | 0.51 | 20 | 58853659 |
|  |  |  |  |  |  | small_scale_variant | MYC | Q52K | 0.51 | 8 | 127738371 |
|  |  |  |  |  |  | small_scale_variant | RAD51D | V66M | 0.46 | 17 | 35118568 |
| Head and Neck | Salivary Adenocarcinoma | 44 | FoundationOne CDx DX1 | 1Muts/Mb() | stable | rearrangement | ETV6-NTRK3 | fusion |  | 15:12 | 88023188-88023188:11882832-  11882832 |
|  |  |  |  |  |  | rearrangement | NTRK3 | rearrangements |  | 15:12 | 88061205-88061205:11883771-  11883771 |
|  |  |  |  |  |  | small_scale_variant | BRCA2 | V2109I | 0.51 | 13 | 32340680 |
|  |  |  |  |  |  | small_scale_variant | CDKN2A | A86fs*29 | 0.26 | 9 | 21971089 |
|  |  |  |  |  |  | small_scale_variant | CDKN2A | A86fs*29 | 0.26 | 9 | 21971089 |
|  |  |  |  |  |  | small_scale_variant | CDKN2A | A86fs*29 | 0.26 | 9 | 21971089 |
|  |  |  |  |  |  | small_scale_variant | ERBB3 | G780E | 0.48 | 12 | 56097109 |
|  |  |  |  |  |  | small_scale_variant | NOTCH3 | A1450T | 0.47 | 19 | 15177580 |
|  |  |  |  |  |  | small_scale_variant | RB1 | E365D | 0.5 | 13 | 48368572 |
|  |  |  |  |  |  | small_scale_variant | SMARCA4 | P171S | 0.13 | 19 | 10986344 |
|  |  |  |  |  |  | small_scale_variant | SMO | K575M | 0.62 | 7 | 129211036 |
|  |  |  |  |  |  | small_scale_variant | TERT | c.-79-45C>T | 0.3 | 5 | 1295113 |
| Head and Neck | Acinic Cell Carcinoma | 60 | FoundationOne CDx DX1 | 0Muts/Mb() | stable | rearrangement | ETV6-NTRK3 | fusion |  | 15:12 | 87941950-87941950:11884353-  11884353 |
|  |  |  |  |  |  | small_scale_variant | KIT | M618I | 0.5 | 4 | 54727902 |
|  |  |  |  |  |  | small_scale_variant | PARP1 | M615V | 0.45 | 1 | 226377206 |
|  |  |  |  |  |  | small_scale_variant | PIK3C2B | Y127C | 0.52 | 1 | 204469423 |
|  |  |  |  |  |  | small_scale_variant | ROS1 | T326R | 0.51 | 6 | 117394618 |
| Pancreas | Pancreas | 46 | FoundationOne Liquid CDx AB1 | 1Muts/Mb() | high not detected | rearrangement | ATP1B1- NTRK1 | fusion |  | 1:01 | 156871724-  156871724:169115234-169115234 |
|  |  |  |  |  |  | small_scale_variant | AXL | R7G | 0.49 | 19 | 41219411 |
|  |  |  |  |  |  | small_scale_variant | CDK8 | R112S | 0.03 | 13 | 26353760 |
|  |  |  |  |  |  | small_scale_variant | CIC | T1043N | 0.47 | 19 | 42292419 |
|  |  |  |  |  |  | small_scale_variant | ERBB2 | V116M | 0.5 | 17 | 39708441 |
|  |  |  |  |  |  | small_scale_variant | ERG | P404A | 0.54 | 21 | 38383633 |
|  |  |  |  |  |  | small_scale_variant | FANCG | A153G | 0.5 | 9 | 35078193 |
|  |  |  |  |  |  | small_scale_variant | JAK1 | T593M | 0.48 | 1 | 64847653 |

|  |  |  |  |  | small_scale_variant | KEL | R292Q | 0.47 | 7 | 142954233 |
| --- | --- | --- | --- | --- | --- | --- | --- | --- | --- | --- |
|  |  |  |  |  | small_scale_variant | NOTCH1 | D1185N | 0.5 | 9 | 136507395 |
|  |  |  |  |  | small_scale_variant | PALB2 | c.1685-1G>C | 0.03 | 16 | 23630470 |
|  |  |  |  |  | small_scale_variant | ZNF217 | D598G | 0.48 | 20 | 53576971 |
| Esophagus/Stomach | 81 | FoundationOne CDx DX1 | 7.57Muts/Mb() | stable | copy_number_alter ation | ATR | amplification |  | 3 | 142445884-142586926 |
|  |  |  |  |  | copy_number_alter  ation | BCL6 | amplification |  | 3 | 187721382-187733693 |
|  |  |  |  |  | copy_number_alter  ation | CCND1 | amplification |  | 11 | 69597245-69687252 |
|  |  |  |  |  | copy_number_alter  ation | CDKN2A | loss |  | 9 | 21853213-21998003 |
|  |  |  |  |  | copy_number_alter  ation | CDKN2B | loss |  | 9 | 21998749-22101833 |
|  |  |  |  |  | copy_number_alter  ation | EMSY | amplification |  | 11 | 76402610-76599854 |
|  |  |  |  |  | copy_number_alter  ation | EPHB1 | amplification |  | 3 | 134925786-135259127 |
|  |  |  |  |  | copy_number_alter  ation | FGF12 | amplification |  | 3 | 192102988-192769418 |
|  |  |  |  |  | copy_number_alter  ation | FGF19 | amplification |  | 11 | 69699261-69747018 |
|  |  |  |  |  | copy_number_alter  ation | FGF3 | amplification |  | 11 | 69765547-69868824 |
|  |  |  |  |  | copy_number_alter  ation | FGF4 | amplification |  | 11 | 69773308-69775084 |
|  |  |  |  |  | copy_number_alter  ation | KDM6A | loss |  | X | 44742591-45237803 |
|  |  |  |  |  | copy_number_alter  ation | KLHL6 | amplification |  | 3 | 183491926-183555653 |
|  |  |  |  |  | copy_number_alter  ation | MAP3K13 | amplification |  | 3 | 185285559-185482470 |
|  |  |  |  |  | copy_number_alter  ation | MEN1 | amplification |  | 11 | 64665597-64946143 |
|  |  |  |  |  | copy_number_alter  ation | MTAP | loss |  | 9 | 21802700-21862059 |
|  |  |  |  |  | copy_number_alter  ation | NKX2-1 | amplification |  | 14 | 36478543-36554296 |
|  |  |  |  |  | copy_number_alter  ation | NTRK1 | amplification |  | 1 | 156815772-156881642 |
|  |  |  |  |  | copy_number_alter  ation | PIK3CA | amplification |  | 3 | 179157482-179278657 |
|  |  |  |  |  | copy_number_alter  ation | PIK3CB | amplification |  | 3 | 138655388-138759343 |
|  |  |  |  |  | copy_number_alter  ation | PRKCI | amplification |  | 3 | 170222655-170303148 |
|  |  |  |  |  | copy_number_alter  ation | SOX2 | amplification |  | 3 | 181665448-181758377 |
|  |  |  |  |  | copy_number_alter  ation | TERC | amplification |  | 3 | 169764212-169765362 |
|  |  |  |  |  | copy_number_alter  ation | TIPARP | amplification |  | 3 | 156677697-156705131 |
|  |  |  |  |  | rearrangement | TPM3-NTRK1 | fusion |  | 1:01 | 156875321-  156875650:154166854-154166990 |
|  |  |  |  |  | small_scale_variant | ATM | D2959H | 0.66 (473/714) | 11 | 108365106 |
|  |  |  |  |  | small_scale_variant | ATM | E1530Q | 0.29 (184/644) | 11 | 108292770 |

|  |  |  |  |  | small_scale_variant | ATRX | S1992del | 0.74 (754/1022) | X | 77593829 |
| --- | --- | --- | --- | --- | --- | --- | --- | --- | --- | --- |
|  |  |  |  |  | small_scale_variant | CDC73 | I189V | 0.19 (217/1156) | 1 | 193141902 |
|  |  |  |  |  | small_scale_variant | CDK12 | N31D | 0.58 (562/967) | 17 | 39462162 |
|  |  |  |  |  | small_scale_variant | GATA4 | R284H | 0.29 (173/604) | 8 | 11750178 |
|  |  |  |  |  | small_scale_variant | NFE2L2 | R34G | 0.47 (569/1199) | 2 | 177234217 |
|  |  |  |  |  | small_scale_variant | NOTCH1 | L5fs*25 | 0.42 (223/528) | 9 | 136545775 |
|  |  |  |  |  | small_scale_variant | NTRK3 | V21F | 0.11 (124/1112) | 15 | 88256093 |
|  |  |  |  |  | small_scale_variant | PDK1 | I397V | 0.63 (633/1005) | 2 | 172595847 |
|  |  |  |  |  | small_scale_variant | TP53 | N131fs*27 | 0.38 (406/1070) | 17 | 7675185 |
|  |  |  |  |  | small_scale_variant | TP53 | V216fs*31 | 0.22 (221/1021) | 17 | 7674883 |
| Soft Tissue | 1 | FoundationOne CDx DX1 | 0.0Muts/Mb() | stable | rearrangement | ETV6-NTRK3 | fusion |  | 15:12 | 87972896-87973252:11878529-  11878796 |
|  |  |  |  |  | small_scale_variant | CCND1 | P54S | 0.66 (595/899) | 11 | 69641473 |
|  |  |  |  |  | small_scale_variant | CIC | N1347S | 0.48 (279/580) | 19 | 42293836 |
|  |  |  |  |  | small_scale_variant | DAXX | D477E | 0.49 (555/1134) | 6 | 33320045 |
|  |  |  |  |  | small_scale_variant | ERBB2 | R1146W | 0.63 (590/934) | 17 | 39727712 |
|  |  |  |  |  | small_scale_variant | ERBB3 | R1118Q | 0.49 (513/1037) | 12 | 56101212 |
|  |  |  |  |  | small_scale_variant | INPP4B | I676T | 0.50 (512/1015) | 4 | 142122236 |
|  |  |  |  |  | small_scale_variant | NOTCH1 | D1185N | 0.51 (216/425) | 9 | 136507395 |
|  |  |  |  |  | small_scale_variant | PIK3CA | I889M | 0.53 (714/1359) | 3 | 179230004 |
|  |  |  |  |  | small_scale_variant | RNF43 | R519Q | 0.40 (471/1190) | 17 | 58358220 |
| Bowel | 48 | FoundationOne CDx DX1 | 5.04Muts/Mb() | stable | copy_number_alter ation | CCND1 | amplification |  | 11 | 69597245-69687252 |
|  |  |  |  |  | copy_number_alter  ation | CCNE1 | amplification |  | 19 | 29763011-29869731 |
|  |  |  |  |  | copy_number_alter  ation | EMSY | amplification |  | 11 | 76408964-76599854 |
|  |  |  |  |  | copy_number_alter  ation | FGF19 | amplification |  | 11 | 69699261-69747018 |
|  |  |  |  |  | copy_number_alter  ation | FGF3 | amplification |  | 11 | 69765547-69868824 |
|  |  |  |  |  | copy_number_alter  ation | FGF4 | amplification |  | 11 | 69773308-69775084 |
|  |  |  |  |  | copy_number_alter  ation | FGFR1 | amplification |  | 8 | 38413627-38505552 |
|  |  |  |  |  | copy_number_alter  ation | NSD3 | amplification |  | 8 | 38275641-38348171 |
|  |  |  |  |  | copy_number_alter  ation | ZNF703 | amplification |  | 8 | 37695979-37698674 |
|  |  |  |  |  | rearrangement | LMNA-NTRK1 | fusion |  | 1:01 | 156874872-  156875048:156131646-156131891 |
|  |  |  |  |  | small_scale_variant | ABL1 | S881del | 0.37 (347/950) | 9 | 130884932 |
|  |  |  |  |  | small_scale_variant | ARID1A | E193_P194delins DS | 0.30 (142/478) | 1 | 26696982 |
|  |  |  |  |  | small_scale_variant | BCL6 | V277L | 0.60 (669/1116) | 3 | 187729576 |
|  |  |  |  |  | small_scale_variant | FOXL2 | T367A | 0.40 (213/531) | 3 | 138945624 |

|  |  |  |  |  |  | small_scale_variant | MAP2K2 | V400M | 0.57 (522/923) | 19 | 4090603 |
| --- | --- | --- | --- | --- | --- | --- | --- | --- | --- | --- | --- |
|  |  |  |  |  |  | small_scale_variant | NOTCH1 | D1185N | 0.39 (286/737) | 9 | 136507395 |
|  |  |  |  |  |  | small_scale_variant | RNF43 | V299M | 0.40 (426/1070) | 17 | 58360206 |
|  |  |  |  |  |  | small_scale_variant | TP53 | R248Q | 0.44 (442/994) | 17 | 7674220 |
| Lung | Lung Adenocarcinoma | 70 | FoundationOne CDx DX1 | 0.0Muts/Mb() | stable | copy_number_alter ation | CDKN2A | loss |  | 9 | 21853213-21998003 |
|  |  |  |  |  |  | copy_number_alter  ation | CDKN2B | loss |  | 9 | 21998749-22101833 |
|  |  |  |  |  |  | copy_number_alter  ation | MTAP | loss |  | 9 | 21802700-21862059 |
|  |  |  |  |  |  | rearrangement | SQSTM1-  NTRK1 | fusion |  | 1:05 | 156874070-  156874512:179829585-179830291 |
|  |  |  |  |  |  | small_scale_variant | CD22 | E266K | 0.52 (145/278) | 19 | 35337832 |
|  |  |  |  |  |  | small_scale_variant | DNMT3A | I681fs*32 | 0.30 (83/273) | 2 | 25241604 |
|  |  |  |  |  |  | small_scale_variant | NTRK3 | V21F | 0.71 (140/197) | 15 | 88256093 |
|  |  |  |  |  |  | small_scale_variant | TSC2 | I1357L | 0.50 (113/224) | 16 | 2084291 |
| Testis | Testis | 73 | FoundationOne CDx DX1 | 0.0Muts/Mb() | stable | copy_number_alter ation | CDK4 | amplification |  | 12 | 57700149-57794361 |
|  |  |  |  |  |  | copy_number_alter  ation | DDR2 | amplification |  | 1 | 162719039-162780246 |
|  |  |  |  |  |  | copy_number_alter  ation | JUN | amplification |  | 1 | 58736932-58832848 |
|  |  |  |  |  |  | copy_number_alter  ation | MDM2 | amplification |  | 12 | 68760216-68876270 |
|  |  |  |  |  |  | copy_number_alter  ation | SDHC | amplification |  | 1 | 161314350-161364810 |
|  |  |  |  |  |  | rearrangement | NTRK1-GRIP1 | fusion |  | 1:12 | 156873732-156873925:66487052-  66487467 |
|  |  |  |  |  |  | small_scale_variant | CTNNA1 | Y245H | 0.48 (413/866) | 5 | 138824674 |
|  |  |  |  |  |  | small_scale_variant | GATA3 | P42S | 0.49 (301/617) | 10 | 8055779 |
|  |  |  |  |  |  | small_scale_variant | KMT2D | R4420W | 0.52 (432/829) | 12 | 49031447 |
|  |  |  |  |  |  | small_scale_variant | NOTCH1 | D1185N | 0.47 (184/392) | 9 | 136507395 |
| Head and Neck | Acinic Cell Carcinoma | 67 | FoundationOne CDx DX1 | 0.0Muts/Mb() | stable | copy_number_alter ation | CDKN2A | loss |  | 9 | 21984653-21998003 |
|  |  |  |  |  |  | copy_number_alter  ation | CDKN2B | loss |  | 9 | 21998749-22048814 |
|  |  |  |  |  |  | copy_number_alter  ation | EPHA3 | amplification |  | 3 | 89107730-89479511 |
|  |  |  |  |  |  | copy_number_alter  ation | SDHA | amplification |  | 5 | 218322-256441 |
|  |  |  |  |  |  | rearrangement | ETV6-NTRK3 | fusion |  | 15:12 | 87976120-87976120:11881799-  11881799 |
|  |  |  |  |  |  | small_scale_variant | LTK | R606Q | 0.50 (358/719) | 15 | 41505411 |
|  |  |  |  |  |  | small_scale_variant | MSH2 | A230fs*2 | 0.10 (61/593) | 2 | 47412455 |
|  |  |  |  |  |  | small_scale_variant | PDGFRB | E460K | 0.44 (260/590) | 5 | 150129958 |
|  |  |  |  |  |  | small_scale_variant | PTPRO | G174E | 0.47 (300/644) | 12 | 15499454 |
|  |  |  |  |  |  | small_scale_variant | RAD51C | T288A | 0.54 (364/678) | 17 | 58720770 |
|  |  |  |  |  |  | small_scale_variant | TYRO3 | I339V | 0.45 (310/694) | 15 | 41568270 |

| Soft Tissue | Well-Differentiated Liposarcoma | 70 | FoundationOne  CDx DX1 | 0.0Muts/Mb() | stable | copy_number_alter  ation | CDK4 | amplification |  | 12 | 57700149-57794361 |
| --- | --- | --- | --- | --- | --- | --- | --- | --- | --- | --- | --- |
|  |  |  |  |  |  | copy_number_alter  ation | JAK3 | amplification |  | 19 | 17826742-17844417 |
|  |  |  |  |  |  | copy_number_alter  ation | MDM2 | amplification |  | 12 | 68760216-68883425 |
|  |  |  |  |  |  | copy_number_alter  ation | MDM2 | amplification |  | 12 | 68760216-68883425 |
|  |  |  |  |  |  | copy_number_alter  ation | NTRK1 | amplification |  | 1 | 156815772-156881642 |
|  |  |  |  |  |  | copy_number_alter  ation | SDHC | amplification |  | 1 | 161314350-161364810 |
|  |  |  |  |  |  | rearrangement | NTRK1-  KIRREL1 | fusion |  | 1:01 | 156873736-  156874023:158050831-158051165 |
|  |  |  |  |  |  | small_scale_variant | BCL2L1 | Q3R | 0.51 (307/599) | 20 | 31722211 |
|  |  |  |  |  |  | small_scale_variant | CARD11 | A687V | 0.39 (235/607) | 7 | 2923214 |
|  |  |  |  |  |  | small_scale_variant | MTOR | Y64S | 0.47 (275/590) | 1 | 11258565 |
|  |  |  |  |  |  | small_scale_variant | ROS1 | R1910* | 0.21 (176/819) | 6 | 117321308 |
|  |  |  |  |  |  | small_scale_variant | TSC1 | T417I | 0.47 (344/736) | 9 | 132910584 |
| Head and Neck | Mammary Analogue Secretory Carcinoma of Salivary Gland Origin | 28 | FoundationOne CDx DX2 | 0Muts/Mb() | stable | rearrangement | ETV6-NTRK3 | fusion |  | 15:12 | 87949297-87949297:11876530-  11876530 |
|  |  |  |  |  |  | small_scale_variant | CIC | N702S | 0.4965 | 19 | 42290873 |
|  |  |  |  |  |  | small_scale_variant | CTNNA1 | N257S | 0.4865 | 5 | 138824711 |
|  |  |  |  |  |  | small_scale_variant | LTK | R658P | 0.4635 | 15 | 41505017 |
|  |  |  |  |  |  | small_scale_variant | MSH3 | R1061G | 0.4848 | 5 | 80873166 |
|  |  |  |  |  |  | small_scale_variant | NTRK1 | R593W | 0.4519 | 1 | 156876544 |
| Thyroid | Papillary Thyroid Cancer | 68 | FoundationOne CDx DX2 | 0Muts/Mb() | stable | rearrangement | TPM3-NTRK1 | fusion |  | 1:01 | 156874113-  156874113:154163360-154163360 |
|  |  |  |  |  |  | small_scale_variant | CARD11 | R608H | 0.5099 | 7 | 2924350 |
|  |  |  |  |  |  | small_scale_variant | CCND3 | R50Q | 0.4827 | 6 | 41941501 |
|  |  |  |  |  |  | small_scale_variant | MAP3K13 | I523V | 0.5218 | 3 | 185466887 |
|  |  |  |  |  |  | small_scale_variant | MSH3 | F709L | 0.4815 | 5 | 80768875 |
|  |  |  |  |  |  | small_scale_variant | ROS1 | T210A | 0.4742 | 6 | 117397066 |
|  |  |  |  |  |  | small_scale_variant | SDHD | V111I | 0.4672 | 11 | 112094821 |
|  |  |  |  |  |  | small_scale_variant | TERT | c.-79-45C>T | 0.1542 | 5 | 1295113 |
|  |  |  |  |  |  | small_scale_variant | TSC1 | S829R | 0.4569 | 9 | 132901606 |
