# Supplementary material for: Prevalence of neurotrophic tropomyosin receptor kinase (NTRK) fusion gene positivity in patients with solid tumors in Japan
Source: Cancer Med. 2024 Jun 25;13(12):e7351. doi: 10.1002/cam4.7351 (PMC11199329; doi:10.1002/cam4.7351)
Supplement: Supplementary file 4 — Table S4. [file CAM4-13-e7351-s001.docx]

Supplementary Table 4 *NTRK* gene fusions previously reported or unreported

| ***NTRK* fusion** | **FusionGDB2** | **TCGA Fusion Gene Database** | **FPIA** | **Mitelman Database of Chromosome Aberrations and Gene Fusions in Cancer** | **COSMIC**  **Fusions** | **Literature search** | **Unreported** |
| --- | --- | --- | --- | --- | --- | --- | --- |
| *AGTPBP1::NTRK2* | No data | No data | No data | No data | No data | Reported |  |
| *ARHGEF11::NTRK1* | No data | No data | No data | Known | No data | Reported |  |
| *ARHGEF2::NTRK1* | Known |  |  |  |  |  |  |
| *ATP1B1::NTRK1* | No data | No data | No data | Known |  |  |  |
| *C1orf226::NTRK1* | No data | No data | No data | No data | No data | Not identified | Unreported |
| *CHIC1::NTRK1* | No data | No data | No data | No data | No data | Not identified | Unreported |
| *CRB1::NTRK1* | No data | No data | No data | No data | No data | Not identified | Unreported |
| *DCAF12::NTRK2* | No data | No data | No data | No data | No data | Not identified | Unreported |
| *ETV6::NTRK3* | Known |  |  |  |  |  |  |
| *GP2::NTRK1* | No data | No data | No data | No data | No data | Reported |  |
| *KANK4::NTRK1* | No data | No data | No data | No data | No data | Not identified | Unreported |
| *KIRREL::NTRK1** | No data | No data | No data | No data | No data | Not identified | Unreported |
| *LMNA::NTRK1* | Known |  |  |  |  |  |  |
| *MEF2D::NTRK1* | No data | No data | No data | No data | No data | Reported |  |
| *NRG1::NTRK2* | No data | No data | No data | No data | No data | Not identified | Unreported |
| *NTRK1::BCAN* | Known |  |  |  |  |  |  |
| *NTRK1::BPI* | No data | No data | No data | No data | No data | Not identified | Unreported |
| *NTRK1::GRIP1* | No data | No data | No data | No data | No data | Not identified | Unreported |
| *NTRK1::KIRREL1* | No data | No data | No data | No data | No data | Not identified | Unreported |

| *NTRK1::LPPR1* | No data | No data | No data | No data | No data | Not identified | Unreported |
| --- | --- | --- | --- | --- | --- | --- | --- |
| *NTRK1::NPTXR* | No data | No data | No data | No data | No data | Not identified | Unreported |
| *NTRK1::SLC25A44* | No data | No data | No data | No data | No data | Reported |  |
| *NTRK2::CLEC16A* | No data | No data | No data | No data | No data | Not identified | Unreported |
| *NTRK2::FGF7* | No data | No data | No data | No data | No data | Not identified | Unreported |
| *NTRK3::CNTN1* | No data | No data | No data | No data | No data | Not identified | Unreported |
| *NTRK3::ETV6* | Known |  |  |  |  |  |  |
| *NTRK3::IGF1R* | No data | No data | No data | No data | No data | Not identified | Unreported |
| *NTRK3::MTOR* | No data | No data | No data | No data | No data | Not identified | Unreported |
| *NTRK3::UNC13C* | No data | No data | No data | No data | No data | Not identified | Unreported |
| *PBX1::NTRK1* | No data | No data | No data | No data | No data | Not identified | Unreported |
| *PEAR1::NTRK1* | Known | No data | No data | No data | No data | Reported |  |
| *PHF20::NTRK1* | No data | No data | No data | No data | No data | Reported |  |
| *PRCC::NTRK1* | Known |  |  |  |  |  |  |
| *PRRC2C::NTRK1* | No data | No data | No data | No data | No data | Not identified | Unreported |
| *SQSTM1::NTRK1* | Known |  |  |  |  |  |  |
| *SYK::NTRK2* | No data | No data | No data | No data | No data | Not identified | Unreported |
| *THEM5::NTRK1* | No data | No data | No data | No data | No data | Not identified | Unreported |
| *TPM3::NTRK1* | Known |  |  |  |  |  |  |
| *TPR::NTRK1* | No data | No data | No data | No data | Known |  |  |

* “KIRREL” is only displayed in the C-CAT database

**FusionGDB2:** <https://compbio.uth.edu/FusionGDB2/index.html> The University of Texas Health Science Center at Houston, USA

**TCGA Fusion Gene Database:** <https://www.tumorfusions.org/> The Jackson Laboratory, USA

**FPIA:** <http://bioinfo-sysu.com/fpia/#update> Sun Yat-sen University, Guangzhou, Guangdong, China

**Mitelman Database of Chromosome Aberrations and Gene Fusions in Cancer:** <https://mitelmandatabase.isb-cgc.org/mb_search> Supported by National Cancer Institute, USA, the Swedish Cancer Society and the Swedish Childhood Cancer Foundation

**COSMIC Fusions:** <https://cancer.sanger.ac.uk/cosmic> Wellcome Sanger Institute, Hinxton, UK
