# Supplementary material for: Prevalence of neurotrophic tropomyosin receptor kinase (NTRK) fusion gene positivity in patients with solid tumors in Japan
Source: Cancer Med. 2024 Jun 25;13(12):e7351. doi: 10.1002/cam4.7351 (PMC11199329; doi:10.1002/cam4.7351)
Supplement: Supplementary file 5 — Table S5. [file CAM4-13-e7351-s003.docx]

Supplementary Table 5 Microsatellite instability (MSI) and tumor mutational burden (TMB) status in *NTRK* gene fusion-positive patients (n = 91)

| **Status** |  | **n (%)** |
| --- | --- | --- |
|  | *NTRK* gene fusion-positive patients | 91 (100%) |
| MSI status | MSI-high | 3 (3.3%) |
|  | MSI-stable | 76 (83.5%) |
|  | Other | 10 (11.0%) |
|  | Missing data | 2 (2.2%) |
| TMB status | TMB-high (≥10 mutations/Mb) | 8 (8.8%) |
|  | TMB-low (<10 mutations/Mb) | 83 (91.2%) |

Mb, megabase
