# Supplementary material for: Prevalence of neurotrophic tropomyosin receptor kinase (NTRK) fusion gene positivity in patients with solid tumors in Japan
Source: Cancer Med. 2024 Jun 25;13(12):e7351. doi: 10.1002/cam4.7351 (PMC11199329; doi:10.1002/cam4.7351)
Supplement: Supplementary file 6 — Table S6. [file CAM4-13-e7351-s006.docx]

Supplementary Table 6 Co-occurrence of *NTRK* gene fusions with other biomarkers

| Biomarker | Biomarker status (patients, n) | | | Gene profiling panel platform | | | *NTRK* fusion status (patients, n) | | Odds ratio | Confidence interval | Spearman correlation coefficient | *P*  value |
| --- | --- | --- | --- | --- | --- | --- | --- | --- | --- | --- | --- | --- |
|  | Positive | Negative | Total | FoundationOne® CDx | FoundationOne® Liquid CDx | OncoGuide^TM^ NCC  Oncopanel System | Positive | Negative |  |  |  |  |
| TMB-high | 8 | 4,196 | 4,204 | Yes | Yes | Yes | 91 | 46,530 | 0.972 | 0.470, 2.010 | -0.000 | 0.94 |
| MSI-high | 3 | 603 | 606 | Yes | Yes | Yes | 91 | 46,530 | 2.597 | 0.819, 8.229 | 0.008 | 0.092 |
| *TP53* | 20 | 27,385 | 27,405 | Yes | Yes | Yes | 91 | 46,530 | 0.197 | 0.120, 0.324 | -0.033 | <0.001 |
| *KRAS* | 1 | 12,399 | 12,400 | Yes | Yes | Yes | 91 | 46,530 | 0.031 | 0.004, 0.220 | -0.026 | <0.001 |
| *APC* | 4 | 9,569 | 9,573 | Yes | Yes | Yes | 91 | 46,530 | 0.178 | 0.065, 0.484 | -0.018 | <0.001 |
| *CDKN2A* | 18 | 9,066 | 9,084 | Yes | Yes | Yes | 91 | 46,530 | 1.019 | 0.608, 1.708 | 0.000 | 0.943 |
| *KMT2D* | 8 | 7,551 | 7,559 | Yes | Yes | No | 83 | 41,133 | 0.474 | 0.229, 0.984 | -0.010 | 0.04 |
| *BRCA2* | 10 | 7,370 | 7,380 | Yes | Yes | Yes | 91 | 46,530 | 0.656 | 0.340, 1.266 | -0.006 | 0.205 |
| *NOTCH3* | 19 | 6,945 | 6,964 | Yes | Yes | Yes | 91 | 46,530 | 1.504 | 0.907, 2.495 | 0.007 | 0.111 |
| *PIK3CA* | 6 | 6,826 | 6,832 | Yes | Yes | Yes | 91 | 46,530 | 0.411 | 0.179, 0.940 | -0.010 | 0.03 |
| *NOTCH1* | 17 | 6,329 | 6,346 | Yes | Yes | Yes | 91 | 46,530 | 1.459 | 0.861, 2.474 | 0.007 | 0.158 |
| *ATM* | 9 | 6,024 | 6,033 | Yes | Yes | Yes | 91 | 46,530 | 0.738 | 0.371, 1.470 | -0.004 | 0.386 |
| *ARID1A* | 10 | 5,676 | 5,686 | Yes | Yes | Yes | 91 | 46,530 | 0.889 | 0.460, 1.715 | -0.002 | 0.725 |
| *CDKN2B* | 14 | 5,568 | 5,582 | Yes | Yes | No | 83 | 41,133 | 1.296 | 0.729, 2.304 | 0.004 | 0.376 |
| *LTK* | 11 | 5,356 | 5,367 | Yes | Yes | No | 83 | 41,133 | 1.021 | 0.541, 1.926 | 0.000 | 0.95 |

| *ERBB2* | 11 | 4,953 | 4,964 | Yes | Yes | Yes | 91 | 46,530 | 1.154 | 0.614, 2.169 | 0.002 | 0.656 |
| --- | --- | --- | --- | --- | --- | --- | --- | --- | --- | --- | --- | --- |
| *SPEN* | 13 | 4,880 | 4,893 | Yes | Yes | No | 83 | 41,133 | 1.380 | 0.763, 2.496 | 0.005 | 0.285 |
| *STK11* | 11 | 4,839 | 4,850 | Yes | Yes | Yes | 91 | 46,530 | 1.185 | 0.630, 2.227 | 0.002 | 0.598 |
| *GNAS* | 4 | 4,569 | 4,573 | Yes | Yes | Yes | 91 | 46,530 | 0.422 | 0.155, 1.151 | -0.008 | 0.082 |
| *SMAD4* | 3 | 4,559 | 4,562 | Yes | Yes | Yes | 91 | 46,530 | 0.314 | 0.099, 0.992 | -0.010 | 0.037 |
| *ROS1* | 8 | 4,411 | 4,419 | Yes | Yes | Yes | 91 | 46,530 | 0.920 | 0.445, 1.903 | -0.001 | 0.823 |
| *NF1* | 7 | 4,344 | 4,351 | Yes | Yes | Yes | 91 | 46,530 | 0.809 | 0.374, 1.751 | -0.002 | 0.59 |
| *TSC1* | 11 | 4,340 | 4,351 | Yes | Yes | Yes | 91 | 46,530 | 1.337 | 0.711, 2.512 | 0.004 | 0.366 |
| *MTAP* | 11 | 4,003 | 4,014 | Yes | Yes | Yes | 91 | 46,530 | 1.461 | 0.777, 2.746 | 0.005 | 0.236 |
| *PTEN* | 3 | 3,896 | 3,899 | Yes | Yes | Yes | 91 | 46,530 | 0.373 | 0.118, 1.180 | -0.008 | 0.081 |
| *RB1* | 5 | 3,881 | 3,886 | Yes | Yes | Yes | 91 | 46,530 | 0.639 | 0.259, 1.575 | -0.005 | 0.326 |
| *MYC* | 5 | 3,847 | 3,852 | Yes | Yes | Yes | 91 | 46,530 | 0.645 | 0.262, 1.590 | -0.004 | 0.337 |
| *ASXL1* | 1 | 3,548 | 3,549 | Yes | Yes | No | 83 | 41,133 | 0.129 | 0.018, 0.928 | -0.012 | 0.016 |
| *DNMT3A* | 4 | 3,544 | 3,548 | Yes | Yes | No | 83 | 41,133 | 0.537 | 0.197, 1.467 | -0.006 | 0.218 |
| *RAD21* | 2 | 3,506 | 3,508 | Yes | Yes | No | 83 | 41,133 | 0.265 | 0.065, 1.078 | -0.010 | 0.046 |
| *PTCH1* | 9 | 3,477 | 3,486 | Yes | Yes | Yes | 91 | 46,530 | 1.359 | 0.682, 2.707 | 0.004 | 0.381 |
| *TSC2* | 11 | 3,463 | 3,474 | Yes | Yes | Yes | 91 | 46,530 | 1.710 | 0.910, 3.215 | 0.008 | 0.092 |
| *BRAF* | 4 | 3,405 | 3,409 | Yes | Yes | Yes | 91 | 46,530 | 0.582 | 0.214, 1.587 | -0.005 | 0.285 |
| *KMT2A* | 7 | 3,297 | 3,304 | Yes | Yes | No | 83 | 41,133 | 1.057 | 0.487, 2.294 | 0.001 | 0.889 |
| *RNF43* | 8 | 3,294 | 3,302 | Yes | Yes | No | 83 | 41,133 | 1.225 | 0.591, 2.542 | 0.003 | 0.585 |

| *ERBB3* | 6 | 3,285 | 3,291 | Yes | Yes | Yes | 91 | 46,530 | 0.929 | 0.406, 2.128 | -0.001 | 0.862 |
| --- | --- | --- | --- | --- | --- | --- | --- | --- | --- | --- | --- | --- |
| *MSH3* | 7 | 3,223 | 3,230 | Yes | Yes | No | 83 | 41,133 | 1.083 | 0.499, 2.352 | 0.001 | 0.839 |
| *BRCA1* | 4 | 3,219 | 3,223 | Yes | Yes | Yes | 91 | 46,530 | 0.619 | 0.227, 1.686 | -0.004 | 0.343 |
| *FANCA* | 3 | 3,199 | 3,202 | Yes | Yes | No | 83 | 41,133 | 0.445 | 0.140, 1.409 | -0.007 | 0.157 |
| *CIC* | 7 | 3,194 | 3,201 | Yes | Yes | No | 83 | 41,133 | 1.094 | 0.504, 2.375 | 0.001 | 0.82 |
| *SETD2* | 6 | 3,189 | 3,195 | Yes | Yes | Yes | 91 | 46,530 | 0.959 | 0.419, 2.197 | -0.000 | 0.922 |
| *IRS2* | 1 | 3,174 | 3,175 | Yes | Yes | No | 83 | 41,133 | 0.146 | 0.020, 1.048 | -0.011 | 0.026 |
| *KEL* | 2 | 3,165 | 3,167 | Yes | Yes | No | 83 | 41,133 | 0.296 | 0.073, 1.205 | -0.009 | 0.071 |
| *RICTOR* | 3 | 3,139 | 3,142 | Yes | Yes | No | 83 | 41,133 | 0.454 | 0.143, 1.438 | -0.007 | 0.168 |
| *CDK12* | 2 | 3,068 | 3,070 | Yes | Yes | Yes | 91 | 46,530 | 0.318 | 0.078, 1.293 | -0.008 | 0.091 |
| *MAP3K1* | 10 | 3,046 | 3,056 | Yes | Yes | Yes | 91 | 46,530 | 1.762 | 0.913, 3.403 | 0.008 | 0.087 |
| *NTRK1* | 13 | 2,983 | 2,996 | Yes | Yes | Yes | 91 | 46,530 | 2.433 | 1.351, 4.382 | 0.014 | 0.002 |
| *NBN* | 5 | 2,991 | 2,996 | Yes | Yes | No | 83 | 41,133 | 0.817 | 0.331, 2.021 | -0.002 | 0.662 |
| *EP300* | 5 | 2,988 | 2,993 | Yes | Yes | Yes | 91 | 46,530 | 0.847 | 0.344, 2.089 | -0.002 | 0.718 |
| *MTOR* | 4 | 2,939 | 2,943 | Yes | Yes | Yes | 91 | 46,530 | 0.682 | 0.250, 1.859 | -0.003 | 0.452 |
| *ZNF217* | 2 | 2,925 | 2,927 | Yes | Yes | No | 83 | 41,133 | 0.323 | 0.079, 1.312 | -0.008 | 0.096 |
| *TERT* | 11 | 2,908 | 2,919 | Yes | Yes | No | 83 | 41,133 | 2.008 | 1.064, 3.792 | 0.011 | 0.028 |
| *EGFR* | 4 | 2,846 | 2,850 | Yes | Yes | Yes | 91 | 46,530 | 0.706 | 0.259, 1.924 | -0.003 | 0.494 |
| *TET2* | 5 | 2,845 | 2,850 | Yes | Yes | No | 83 | 41,133 | 0.863 | 0.349, 2.133 | -0.002 | 0.749 |
| *DIS3* | 5 | 2,806 | 2,811 | Yes | Yes | No | 83 | 41,133 | 0.876 | 0.354, 2.164 | -0.001 | 0.773 |

| *ATRX* | 3 | 2,783 | 2,786 | Yes | Yes | No | 83 | 41,133 | 0.517 | 0.163, 1.637 | -0.006 | 0.253 |
| --- | --- | --- | --- | --- | --- | --- | --- | --- | --- | --- | --- | --- |
| *KIT* | 4 | 2,782 | 2,786 | Yes | Yes | Yes | 91 | 46,530 | 0.723 | 0.265, 1.971 | -0.003 | 0.524 |
| *NOTCH2* | 5 | 2,762 | 2,767 | Yes | Yes | Yes | 91 | 46,530 | 0.921 | 0.374, 2.272 | -0.001 | 0.859 |
| *CREBBP* | 3 | 2,684 | 2,687 | Yes | Yes | Yes | 91 | 46,530 | 0.557 | 0.176, 1.761 | -0.005 | 0.312 |
| *ALK* | 7 | 2,680 | 2,687 | Yes | Yes | Yes | 91 | 46,530 | 1.363 | 0.630, 2.951 | 0.004 | 0.429 |
| *FLT3* | 3 | 2,673 | 2,676 | Yes | Yes | Yes | 91 | 46,530 | 0.559 | 0.177, 1.769 | -0.005 | 0.316 |
| *CARD11* | 8 | 2,654 | 2,662 | Yes | Yes | No | 83 | 41,133 | 1.547 | 0.745, 3.209 | 0.006 | 0.238 |
| *PALB2* | 6 | 2,650 | 2,656 | Yes | Yes | Yes | 91 | 46,530 | 1.169 | 0.510, 2.677 | 0.002 | 0.712 |
| *PIK3C2G* | 3 | 2,625 | 2,628 | Yes | Yes | No | 83 | 41,133 | 0.550 | 0.174, 1.743 | -0.005 | 0.303 |
| *TGFBR2* | 8 | 2,616 | 2,624 | Yes | Yes | No | 83 | 41,133 | 1.571 | 0.757, 3.259 | 0.006 | 0.222 |
| *SMARCA4* | 4 | 2,594 | 2,598 | Yes | Yes | Yes | 91 | 46,530 | 0.779 | 0.286, 2.123 | -0.002 | 0.624 |
| *MET* | 8 | 2,570 | 2,578 | Yes | Yes | Yes | 91 | 46,530 | 1.649 | 0.797, 3.410 | 0.006 | 0.173 |
| *MSH6* | 2 | 2,564 | 2,566 | Yes | Yes | Yes | 91 | 46,530 | 0.385 | 0.095, 1.566 | -0.006 | 0.166 |
| *CCND1* | 5 | 2,510 | 2,515 | Yes | Yes | Yes | 91 | 46,530 | 1.020 | 0.413, 2.514 | 0.000 | 0.966 |
| *NTRK3* | 3 | 2,483 | 2,486 | Yes | Yes | Yes | 91 | 46,530 | 0.605 | 0.191, 1.913 | -0.004 | 0.387 |
| *MST1R* | 3 | 2,437 | 2,440 | Yes | Yes | No | 83 | 41,133 | 0.595 | 0.188, 1.887 | -0.004 | 0.373 |
| *MAP3K13* | 7 | 2,400 | 2,407 | Yes | Yes | No | 83 | 41,133 | 1.486 | 0.685, 3.227 | 0.005 | 0.313 |
| *FLT1* | 3 | 2,394 | 2,397 | Yes | Yes | No | 83 | 41,133 | 0.607 | 0.191, 1.923 | -0.004 | 0.391 |
| *CHEK2* | 3 | 2,391 | 2,394 | Yes | Yes | Yes | 91 | 46,530 | 0.629 | 0.199, 1.990 | -0.004 | 0.426 |
| *FBXW7* | 4 | 2,385 | 2,389 | Yes | Yes | Yes | 91 | 46,530 | 0.851 | 0.312, 2.321 | -0.001 | 0.752 |

| *ATR* | 4 | 2,379 | 2,383 | Yes | Yes | No | 83 | 41,133 | 0.825 | 0.302, 2.254 | -0.002 | 0.707 |
| --- | --- | --- | --- | --- | --- | --- | --- | --- | --- | --- | --- | --- |
| *FGFR1* | 3 | 2,348 | 2,351 | Yes | Yes | Yes | 91 | 46,530 | 0.641 | 0.203, 2.029 | -0.004 | 0.446 |
| *POLE* | 6 | 2,338 | 2,344 | Yes | Yes | Yes | 91 | 46,530 | 1.334 | 0.582, 3.057 | 0.003 | 0.494 |
| *GATA3* | 6 | 2,331 | 2,337 | Yes | Yes | No | 83 | 41,133 | 1.297 | 0.565, 2.980 | 0.003 | 0.539 |
| *GATA6* | 1 | 2,288 | 2,289 | Yes | Yes | No | 83 | 41,133 | 0.207 | 0.029, 1.488 | -0.009 | 0.083 |
| *FGFR3* | 3 | 2,285 | 2,288 | Yes | Yes | Yes | 91 | 46,530 | 0.660 | 0.209, 2.088 | -0.003 | 0.476 |
| *IKBKE* | 8 | 2,277 | 2,285 | Yes | Yes | No | 83 | 41,133 | 1.820 | 0.877, 3.778 | 0.008 | 0.103 |
| *MSH2* | 7 | 2,253 | 2,260 | Yes | Yes | Yes | 91 | 46,530 | 1.638 | 0.757, 3.545 | 0.006 | 0.206 |
| *KDM5A* | 0 | 2,238 | 2,238 | Yes | Yes | No | 83 | 41,133 | 0.000 | – | -0.011 | 0.029 |
| *NSD3* | 3 | 2,231 | 2,234 | Yes | Yes | No | 83 | 41,133 | 0.654 | 0.206, 2.072 | -0.004 | 0.467 |
| *FANCG* | 5 | 2,226 | 2,231 | Yes | Yes | No | 83 | 41,133 | 1.120 | 0.453, 2.770 | 0.001 | 0.805 |
| *AR* | 3 | 2,161 | 2,164 | Yes | Yes | No | 83 | 41,133 | 0.676 | 0.213, 2.143 | -0.003 | 0.504 |
| *DOT1L* | 4 | 2,159 | 2,163 | Yes | Yes | No | 83 | 41,133 | 0.914 | 0.334, 2.498 | -0.001 | 0.861 |
| *PBRM1* | 3 | 2,148 | 2,151 | Yes | Yes | Yes | 91 | 46,530 | 0.704 | 0.223, 2.228 | -0.003 | 0.549 |
| *CDH1* | 3 | 2,122 | 2,125 | Yes | Yes | No | 83 | 41,133 | 0.689 | 0.218, 2.185 | -0.003 | 0.525 |
| *ERBB4* | 4 | 2,120 | 2,124 | Yes | Yes | Yes | 91 | 46,530 | 0.963 | 0.353, 2.627 | -0.000 | 0.941 |
| *PDGFRB* | 4 | 2,118 | 2,122 | Yes | Yes | Yes | 91 | 46,530 | 0.964 | 0.354, 2.629 | -0.000 | 0.943 |
| *PIK3C2B* | 7 | 2,111 | 2,118 | Yes | Yes | No | 83 | 41,133 | 1.703 | 0.784, 3.697 | 0.007 | 0.174 |
| *DAXX* | 5 | 2,092 | 2,097 | Yes | Yes | No | 83 | 41,133 | 1.196 | 0.484, 2.958 | 0.002 | 0.698 |
| *MUTYH* | 5 | 2,085 | 2,090 | Yes | Yes | No | 83 | 41,133 | 1.201 | 0.486, 2.968 | 0.002 | 0.692 |

| *CTNNA1* | 4 | 2,076 | 2,080 | Yes | Yes | No | 83 | 41,133 | 0.953 | 0.349, 2.604 | -0.000 | 0.925 |
| --- | --- | --- | --- | --- | --- | --- | --- | --- | --- | --- | --- | --- |
| *CTNNB1* | 2 | 2,068 | 2,070 | Yes | Yes | Yes | 91 | 46,530 | 0.483 | 0.119, 1.963 | -0.005 | 0.299 |
| *FGF3* | 4 | 2,065 | 2,069 | Yes | Yes | No | 83 | 41,133 | 0.958 | 0.350, 2.618 | -0.000 | 0.933 |
| *FGF19* | 3 | 2,017 | 2,020 | Yes | Yes | No | 83 | 41,133 | 0.727 | 0.229, 2.305 | -0.003 | 0.587 |
| *CD22* | 3 | 2,001 | 2,004 | Yes | Yes | No | 83 | 41,133 | 0.733 | 0.231, 2.324 | -0.003 | 0.597 |
| *NSD2* | 1 | 2,001 | 2,002 | Yes | Yes | No | 83 | 41,133 | 0.238 | 0.033, 1.714 | -0.008 | 0.121 |
| *BRIP1* | 4 | 1,995 | 1,999 | Yes | Yes | No | 83 | 41,133 | 0.993 | 0.363, 2.715 | -0.000 | 0.99 |
| *KDM6A* | 3 | 1,981 | 1,984 | Yes | Yes | Yes | 91 | 46,530 | 0.767 | 0.242, 2.425 | -0.002 | 0.65 |
| *BCOR* | 2 | 1,979 | 1,981 | Yes | Yes | No | 83 | 41,133 | 0.489 | 0.120, 1.988 | -0.005 | 0.307 |
| *BRD4* | 3 | 1,970 | 1,973 | Yes | Yes | No | 83 | 41,133 | 0.745 | 0.235, 2.363 | -0.002 | 0.616 |
| *FGF4* | 5 | 1,953 | 1,958 | Yes | Yes | No | 83 | 41,133 | 1.286 | 0.520, 3.180 | 0.003 | 0.585 |
| *TBX3* | 2 | 1,946 | 1,948 | Yes | Yes | No | 83 | 41,133 | 0.497 | 0.122, 2.024 | -0.005 | 0.319 |
| *POLD1* | 0 | 1,916 | 1,916 | Yes | Yes | Yes | 91 | 46,530 | 0.000 | – | -0.009 | 0.048 |
| *ZNF703* | 2 | 1,911 | 1,913 | Yes | Yes | No | 83 | 41,133 | 0.507 | 0.125, 2.063 | -0.005 | 0.333 |
| *AXL* | 3 | 1,892 | 1,895 | Yes | Yes | Yes | 91 | 46,530 | 0.804 | 0.254, 2.544 | -0.002 | 0.71 |
| *EPHA3* | 4 | 1,885 | 1,889 | Yes | Yes | No | 83 | 41,133 | 1.054 | 0.386, 2.882 | 0.001 | 0.918 |
| *PTPRO* | 3 | 1,860 | 1,863 | Yes | Yes | No | 83 | 41,133 | 0.792 | 0.250, 2.510 | -0.002 | 0.691 |
| *VEGFA* | 3 | 1,855 | 1,858 | Yes | Yes | No | 83 | 41,133 | 0.794 | 0.251, 2.517 | -0.002 | 0.695 |
| *RET* | 4 | 1,836 | 1,840 | Yes | Yes | Yes | 91 | 46,530 | 1.119 | 0.410, 3.053 | 0.001 | 0.826 |
| *MDM2* | 6 | 1,809 | 1,815 | Yes | Yes | Yes | 91 | 46,530 | 1.745 | 0.761, 3.999 | 0.006 | 0.183 |

| *CCNE1* | 2 | 1,803 | 1,805 | Yes | Yes | Yes | 91 | 46,530 | 0.557 | 0.137, 2.266 | -0.004 | 0.407 |
| --- | --- | --- | --- | --- | --- | --- | --- | --- | --- | --- | --- | --- |
| *SDHA* | 2 | 1,802 | 1,804 | Yes | Yes | No | 83 | 41,133 | 0.539 | 0.132, 2.194 | -0.004 | 0.381 |
| *RAD51D* | 6 | 1,785 | 1,791 | Yes | Yes | No | 83 | 41,133 | 1.718 | 0.747, 3.948 | 0.006 | 0.197 |
| *BCORL1* | 2 | 1,755 | 1,757 | Yes | Yes | No | 83 | 41,133 | 0.554 | 0.136, 2.255 | -0.004 | 0.403 |
| *AURKA* | 0 | 1,752 | 1,752 | Yes | Yes | No | 83 | 41,133 | 0.000 | – | -0.009 | 0.055 |
| *KDR* | 3 | 1,742 | 1,745 | Yes | Yes | No | 83 | 41,133 | 0.848 | 0.268, 2.688 | -0.001 | 0.779 |
| *PRKCI* | 2 | 1,740 | 1,742 | Yes | Yes | Yes | 91 | 46,530 | 0.578 | 0.142, 2.351 | -0.004 | 0.439 |
| *JAK3* | 3 | 1,718 | 1,721 | Yes | Yes | Yes | 91 | 46,530 | 0.889 | 0.281, 2.813 | -0.001 | 0.842 |
| *PDGFRA* | 4 | 1,714 | 1,718 | Yes | Yes | Yes | 91 | 46,530 | 1.202 | 0.441, 3.279 | 0.002 | 0.719 |
| *EPHB4* | 3 | 1,700 | 1,703 | Yes | Yes | No | 83 | 41,133 | 0.870 | 0.274, 2.757 | -0.001 | 0.813 |
| *BCL2L1* | 1 | 1,687 | 1,688 | Yes | Yes | No | 83 | 41,133 | 0.285 | 0.040, 2.050 | -0.007 | 0.183 |
| *MLH1* | 2 | 1,684 | 1,686 | Yes | Yes | Yes | 91 | 46,530 | 0.598 | 0.147, 2.432 | -0.003 | 0.468 |
| *SMO* | 6 | 1,676 | 1,682 | Yes | Yes | Yes | 91 | 46,530 | 1.889 | 0.824, 4.330 | 0.007 | 0.126 |
| *AMER1* | 3 | 1,675 | 1,678 | Yes | Yes | No | 83 | 41,133 | 0.883 | 0.279, 2.800 | -0.001 | 0.833 |
| *DDR1* | 2 | 1,659 | 1,661 | Yes | Yes | No | 83 | 41,133 | 0.588 | 0.144, 2.391 | -0.004 | 0.452 |
| *FGFR4* | 2 | 1,626 | 1,628 | Yes | Yes | Yes | 91 | 46,530 | 0.621 | 0.153, 2.522 | -0.003 | 0.501 |
| *EPHB1* | 4 | 1,592 | 1,596 | Yes | Yes | No | 83 | 41,133 | 1.258 | 0.460, 3.438 | 0.002 | 0.654 |
| *ARFRP1* | 1 | 1,594 | 1,595 | Yes | Yes | No | 83 | 41,133 | 0.302 | 0.042, 2.175 | -0.006 | 0.208 |
| *PARP3* | 0 | 1,587 | 1,587 | Yes | Yes | No | 83 | 41,133 | 0.000 | – | -0.009 | 0.068 |
| *SRC* | 1 | 1,586 | 1,587 | Yes | Yes | No | 83 | 41,133 | 0.304 | 0.042, 2.186 | -0.006 | 0.21 |

| *PIK3R1* | 2 | 1,570 | 1,572 | Yes | Yes | Yes | 91 | 46,530 | 0.644 | 0.158, 2.616 | -0.003 | 0.535 |
| --- | --- | --- | --- | --- | --- | --- | --- | --- | --- | --- | --- | --- |
| *TEK* | 1 | 1,569 | 1,570 | Yes | Yes | No | 83 | 41,133 | 0.308 | 0.043, 2.211 | -0.006 | 0.215 |
| *ESR1* | 4 | 1,560 | 1,564 | Yes | Yes | Yes | 91 | 46,530 | 1.325 | 0.486, 3.616 | 0.003 | 0.581 |
| *RAD52* | 0 | 1,554 | 1,554 | Yes | Yes | No | 83 | 41,133 | 0.000 | – | -0.009 | 0.071 |
| *ABL1* | 5 | 1,536 | 1,541 | Yes | Yes | Yes | 91 | 46,530 | 1.703 | 0.690, 4.202 | 0.005 | 0.242 |
| *KLHL6* | 4 | 1,530 | 1,534 | Yes | Yes | No | 83 | 41,133 | 1.311 | 0.479, 3.583 | 0.003 | 0.597 |
| *NFE2L2* | 3 | 1,510 | 1,513 | Yes | Yes | Yes | 91 | 46,530 | 1.016 | 0.321, 3.216 | 0.000 | 0.978 |
| *HGF* | 2 | 1,507 | 1,509 | Yes | Yes | No | 83 | 41,133 | 0.649 | 0.159, 2.643 | -0.003 | 0.543 |
| *CBL* | 3 | 1,490 | 1,493 | Yes | Yes | No | 83 | 41,133 | 0.998 | 0.315, 3.163 | -0.000 | 0.997 |
| *GRM3* | 4 | 1,483 | 1,487 | Yes | Yes | No | 83 | 41,133 | 1.354 | 0.495, 3.701 | 0.003 | 0.554 |
| *MED12* | 0 | 1,483 | 1,483 | Yes | Yes | No | 83 | 41,133 | 0.000 | – | -0.009 | 0.078 |
| *JAK2* | 4 | 1,473 | 1,477 | Yes | Yes | Yes | 91 | 46,530 | 1.406 | 0.516, 3.837 | 0.003 | 0.503 |
| *EMSY* | 4 | 1,468 | 1,472 | Yes | Yes | No | 83 | 41,133 | 1.368 | 0.500, 3.741 | 0.003 | 0.54 |
| *DDR2* | 4 | 1,462 | 1,466 | Yes | Yes | Yes | 91 | 46,530 | 1.417 | 0.520, 3.867 | 0.003 | 0.494 |
| *IGF1R* | 2 | 1,434 | 1,436 | Yes | Yes | Yes | 91 | 46,530 | 0.707 | 0.174, 2.873 | -0.002 | 0.626 |
| *PRDM1* | 2 | 1,408 | 1,410 | Yes | Yes | No | 83 | 41,133 | 0.697 | 0.171, 2.836 | -0.002 | 0.612 |
| *CEBPA* | 1 | 1,405 | 1,406 | Yes | Yes | No | 83 | 41,133 | 0.345 | 0.048, 2.479 | -0.005 | 0.268 |
| *LYN* | 3 | 1,391 | 1,394 | Yes | Yes | No | 83 | 41,133 | 1.071 | 0.338, 3.397 | 0.001 | 0.907 |
| *KEAP1* | 2 | 1,384 | 1,386 | Yes | Yes | Yes | 91 | 46,530 | 0.733 | 0.180, 2.980 | -0.002 | 0.663 |
| *FGFR2* | 1 | 1,355 | 1,356 | Yes | Yes | Yes | 91 | 46,530 | 0.370 | 0.052, 2.661 | -0.005 | 0.304 |

| *CDK4* | 7 | 1,328 | 1,335 | Yes | Yes | Yes | 91 | 46,530 | 2.836 | 1.309, 6.144 | 0.013 | 0.006 |
| --- | --- | --- | --- | --- | --- | --- | --- | --- | --- | --- | --- | --- |
| *RPTOR* | 3 | 1,308 | 1,311 | Yes | Yes | No | 83 | 41,133 | 1.142 | 0.360, 3.620 | 0.001 | 0.822 |
| *SOX9* | 1 | 1,310 | 1,311 | Yes | Yes | No | 83 | 41,133 | 0.371 | 0.052, 2.665 | -0.005 | 0.304 |
| *AXIN1* | 2 | 1,294 | 1,296 | Yes | Yes | Yes | 91 | 46,530 | 0.786 | 0.193, 3.194 | -0.002 | 0.735 |
| *BAP1* | 2 | 1,290 | 1,292 | Yes | Yes | Yes | 91 | 46,530 | 0.788 | 0.194, 3.204 | -0.002 | 0.739 |
| *CUL4A* | 1 | 1,286 | 1,287 | Yes | Yes | No | 83 | 41,133 | 0.378 | 0.053, 2.717 | -0.005 | 0.315 |
| *BCL6* | 4 | 1,272 | 1,276 | Yes | Yes | No | 83 | 41,133 | 1.587 | 0.580, 4.339 | 0.004 | 0.364 |
| *MPL* | 0 | 1,260 | 1,260 | Yes | Yes | No | 83 | 41,133 | 0.000 | – | -0.008 | 0.105 |
| *PARP1* | 3 | 1,255 | 1,258 | Yes | Yes | No | 83 | 41,133 | 1.192 | 0.376, 3.778 | 0.001 | 0.766 |
| *HSD3B1* | 0 | 1,247 | 1,247 | Yes | Yes | No | 83 | 41,133 | 0.000 | – | -0.008 | 0.107 |
| *MERTK* | 1 | 1,232 | 1,233 | Yes | Yes | No | 83 | 41,133 | 0.395 | 0.055, 2.840 | -0.005 | 0.339 |
| *MAP2K4* | 0 | 1,214 | 1,214 | Yes | Yes | Yes | 91 | 46,530 | 0.000 | – | -0.007 | 0.118 |
| *MAP2K2* | 3 | 1,211 | 1,214 | Yes | Yes | Yes | 91 | 46,530 | 1.276 | 0.403, 4.038 | 0.002 | 0.678 |
| *CDK8* | 2 | 1,201 | 1,203 | Yes | Yes | No | 83 | 41,133 | 0.821 | 0.202, 3.343 | -0.001 | 0.783 |
| *ERCC4* | 4 | 1,178 | 1,182 | Yes | Yes | No | 83 | 41,133 | 1.717 | 0.628, 4.697 | 0.005 | 0.286 |
| *MCL1* | 3 | 1,163 | 1,166 | Yes | Yes | No | 83 | 41,133 | 1.289 | 0.406, 4.087 | 0.002 | 0.666 |
| *PMS2* | 1 | 1,165 | 1,166 | Yes | Yes | Yes | 91 | 46,530 | 0.433 | 0.060, 3.108 | -0.004 | 0.391 |
| *GABRA6* | 1 | 1,158 | 1,159 | Yes | Yes | No | 83 | 41,133 | 0.421 | 0.059, 3.027 | -0.004 | 0.375 |
| *SOX2* | 2 | 1,152 | 1,154 | Yes | Yes | No | 83 | 41,133 | 0.857 | 0.210, 3.490 | -0.001 | 0.829 |
| *FGF6* | 1 | 1,150 | 1,151 | Yes | Yes | No | 83 | 41,133 | 0.424 | 0.059, 3.049 | -0.004 | 0.379 |

| *FGF23* | 0 | 1,140 | 1,140 | Yes | Yes | No | 83 | 41,133 | 0.000 | – | -0.008 | 0.124 |
| --- | --- | --- | --- | --- | --- | --- | --- | --- | --- | --- | --- | --- |
| *SNCAIP* | 0 | 1,137 | 1,137 | Yes | Yes | No | 83 | 41,133 | 0.000 | – | -0.008 | 0.125 |
| *FUBP1* | 0 | 1,133 | 1,133 | Yes | Yes | No | 83 | 41,133 | 0.000 | – | -0.008 | 0.125 |
| *BARD1* | 4 | 1,121 | 1,125 | Yes | Yes | Yes | 91 | 46,530 | 1.862 | 0.682, 5.083 | 0.006 | 0.217 |
| *JAK1* | 4 | 1,114 | 1,118 | Yes | Yes | Yes | 91 | 46,530 | 1.874 | 0.687, 5.116 | 0.006 | 0.212 |
| *SF3B1* | 1 | 1,113 | 1,114 | Yes | Yes | No | 83 | 41,133 | 0.438 | 0.061, 3.153 | -0.004 | 0.4 |
| *AKT1* | 1 | 1,106 | 1,107 | Yes | Yes | Yes | 91 | 46,530 | 0.456 | 0.064, 3.278 | -0.004 | 0.424 |
| *NRAS* | 1 | 1,102 | 1,103 | Yes | Yes | Yes | 91 | 46,530 | 0.458 | 0.064, 3.290 | -0.004 | 0.426 |
| *AKT2* | 1 | 1,095 | 1,096 | Yes | Yes | Yes | 91 | 46,530 | 0.461 | 0.064, 3.312 | -0.004 | 0.43 |
| *NKX2-1* | 3 | 1,082 | 1,085 | Yes | Yes | No | 83 | 41,133 | 1.388 | 0.438, 4.402 | 0.003 | 0.576 |
| *FGF14* | 0 | 1,077 | 1,077 | Yes | Yes | No | 83 | 41,133 | 0.000 | – | -0.007 | 0.135 |
| *RBM10* | 2 | 1,074 | 1,076 | Yes | Yes | No | 83 | 41,133 | 0.921 | 0.226, 3.751 | -0.001 | 0.908 |
| *FANCC* | 2 | 1,069 | 1,071 | Yes | Yes | No | 83 | 41,133 | 0.925 | 0.227, 3.769 | -0.001 | 0.914 |
| *MAF* | 4 | 1,067 | 1,071 | Yes | Yes | No | 83 | 41,133 | 1.901 | 0.695, 5.201 | 0.006 | 0.203 |
| *TERC* | 1 | 1,068 | 1,069 | Yes | Yes | No | 83 | 41,133 | 0.457 | 0.064, 3.290 | -0.004 | 0.426 |
| *IDH1* | 2 | 1,063 | 1,065 | Yes | Yes | Yes | 91 | 46,530 | 0.961 | 0.236, 3.908 | -0.000 | 0.956 |
| *CSF3R* | 3 | 1,051 | 1,054 | Yes | Yes | No | 83 | 41,133 | 1.430 | 0.451, 4.536 | 0.003 | 0.541 |
| *MYCL* | 0 | 1,045 | 1,045 | Yes | Yes | No | 83 | 41,133 | 0.000 | – | -0.007 | 0.141 |
| *EZH2* | 2 | 1,009 | 1,011 | Yes | Yes | Yes | 91 | 46,530 | 1.014 | 0.249, 4.123 | 0.000 | 0.985 |
| *IDH2* | 0 | 1,002 | 1,002 | Yes | Yes | Yes | 91 | 46,530 | 0.000 | – | -0.007 | 0.157 |

| *RARA* | 1 | 994 | 995 | Yes | Yes | No | 83 | 41133 | 0.492 | 0.068, 3.542 | -0.004 | 0.472 |
| --- | --- | --- | --- | --- | --- | --- | --- | --- | --- | --- | --- | --- |
| *PIK3CB* | 3 | 969 | 972 | Yes | Yes | No | 83 | 41133 | 1.554 | 0.490, 4.930 | 0.004 | 0.45 |
| *WT1* | 1 | 970 | 971 | Yes | Yes | No | 83 | 41133 | 0.505 | 0.070, 3.631 | -0.003 | 0.489 |
| *NF2* | 2 | 957 | 959 | Yes | Yes | Yes | 91 | 46530 | 1.070 | 0.263, 4.352 | 0.000 | 0.925 |
| *PARP2* | 1 | 958 | 959 | Yes | Yes | No | 83 | 41,133 | 0.511 | 0.071, 3.678 | -0.003 | 0.497 |
| *CCND2* | 0 | 954 | 954 | Yes | Yes | No | 83 | 41,133 | 0.000 | – | -0.007 | 0.16 |
| *MRE11* | 2 | 946 | 948 | Yes | Yes | No | 83 | 41,133 | 1.049 | 0.258, 4.272 | 0.000 | 0.947 |
| *CSF1R* | 1 | 936 | 937 | Yes | Yes | No | 83 | 41,133 | 0.524 | 0.073, 3.767 | -0.003 | 0.513 |
| *TIPARP* | 4 | 930 | 934 | Yes | Yes | No | 83 | 41,133 | 2.189 | 0.800, 5.989 | 0.008 | 0.118 |
| *GATA4* | 2 | 928 | 930 | Yes | Yes | No | 83 | 41,133 | 1.070 | 0.263, 4.357 | 0.000 | 0.925 |
| *CCND3* | 1 | 916 | 917 | Yes | Yes | No | 83 | 41,133 | 0.535 | 0.074, 3.851 | -0.003 | 0.528 |
| *FGF10* | 0 | 909 | 909 | Yes | Yes | No | 83 | 41,133 | 0.000 | – | -0.007 | 0.171 |
| *INPP4B* | 2 | 900 | 902 | Yes | Yes | No | 83 | 41,133 | 1.104 | 0.271, 4.496 | 0.001 | 0.89 |
| *MYCN* | 1 | 900 | 901 | Yes | Yes | Yes | 91 | 46,530 | 0.563 | 0.078, 4.047 | -0.003 | 0.563 |
| *FLCN* | 2 | 897 | 899 | Yes | Yes | No | 83 | 41,133 | 1.108 | 0.272, 4.512 | 0.001 | 0.887 |
| *MEN1* | 1 | 891 | 892 | Yes | Yes | Yes | 91 | 46,530 | 0.569 | 0.079, 4.089 | -0.003 | 0.57 |
| *FH* | 1 | 885 | 886 | Yes | Yes | No | 83 | 41,133 | 0.555 | 0.077, 3.989 | -0.003 | 0.552 |
| *RAD54L* | 1 | 880 | 881 | Yes | Yes | No | 83 | 41,133 | 0.558 | 0.078, 4.012 | -0.003 | 0.556 |
| *RAD51C* | 2 | 878 | 880 | Yes | Yes | Yes | 91 | 46,530 | 1.168 | 0.287, 4.753 | 0.001 | 0.828 |
| *PRKN* | 0 | 878 | 878 | Yes | Yes | No | 83 | 41,133 | 0.000 | – | -0.007 | 0.178 |

| *NTRK2* | 4 | 868 | 872 | Yes | Yes | Yes | 91 | 46,530 | 2.419 | 0.886, 6.604 | 0.008 | 0.075 |
| --- | --- | --- | --- | --- | --- | --- | --- | --- | --- | --- | --- | --- |
| *KDM5C* | 0 | 872 | 872 | Yes | Yes | No | 83 | 41,133 | 0.000 | – | -0.007 | 0.18 |
| *SPOP* | 0 | 868 | 868 | Yes | Yes | No | 83 | 41,133 | 0.000 | – | -0.007 | 0.181 |
| *IKZF1* | 2 | 865 | 867 | Yes | Yes | No | 83 | 41,133 | 1.149 | 0.282, 4.682 | 0.001 | 0.846 |
| *MDM4* | 1 | 865 | 866 | Yes | Yes | Yes | 91 | 46,530 | 0.587 | 0.082, 4.215 | -0.002 | 0.592 |
| *CDK6* | 2 | 864 | 866 | Yes | Yes | Yes | 91 | 46,530 | 1.188 | 0.292, 4.831 | 0.001 | 0.81 |
| *PPP2R1A* | 1 | 851 | 852 | Yes | Yes | No | 83 | 41,133 | 0.577 | 0.080, 4.152 | -0.003 | 0.58 |
| *PAX5* | 0 | 849 | 849 | Yes | Yes | No | 83 | 41,133 | 0.000 | – | -0.007 | 0.186 |
| *CDKN1B* | 0 | 840 | 840 | Yes | Yes | No | 83 | 41,133 | 0.000 | – | -0.006 | 0.188 |
| *TNFAIP3* | 1 | 833 | 834 | Yes | Yes | No | 83 | 41,133 | 0.590 | 0.082, 4.244 | -0.003 | 0.596 |
| *FANCL* | 2 | 829 | 831 | Yes | Yes | No | 83 | 41,133 | 1.200 | 0.295, 4.891 | 0.001 | 0.799 |
| *CALR* | 2 | 826 | 828 | Yes | Yes | No | 83 | 41,133 | 1.205 | 0.296, 4.909 | 0.001 | 0.795 |
| *FGF12* | 2 | 825 | 827 | Yes | Yes | No | 83 | 41,133 | 1.206 | 0.296, 4.915 | 0.001 | 0.793 |
| *ACVR1B* | 0 | 825 | 825 | Yes | Yes | No | 83 | 41,133 | 0.000 | – | -0.006 | 0.192 |
| *ARAF* | 1 | 820 | 821 | Yes | Yes | Yes | 91 | 46,530 | 0.619 | 0.086, 4.451 | -0.002 | 0.631 |
| *CASP8* | 3 | 811 | 814 | Yes | Yes | No | 83 | 41,133 | 1.864 | 0.588, 5.916 | 0.005 | 0.283 |
| *TYRO3* | 2 | 811 | 813 | Yes | Yes | No | 83 | 41,133 | 1.228 | 0.301, 5.002 | 0.001 | 0.774 |
| *RAD51B* | 1 | 812 | 813 | Yes | Yes | No | 83 | 41,133 | 0.606 | 0.084, 4.356 | -0.002 | 0.615 |
| *AKT3* | 2 | 805 | 807 | Yes | Yes | Yes | 91 | 46,530 | 1.276 | 0.314, 5.193 | 0.002 | 0.733 |
| *SGK1* | 1 | 803 | 804 | Yes | Yes | No | 83 | 41,133 | 0.612 | 0.085, 4.406 | -0.002 | 0.623 |

| *STAG2* | 2 | 798 | 800 | Yes | Yes | No | 83 | 41,133 | 1.248 | 0.306, 5.085 | 0.002 | 0.757 |
| --- | --- | --- | --- | --- | --- | --- | --- | --- | --- | --- | --- | --- |
| *PDCD1LG2* | 2 | 763 | 765 | Yes | Yes | No | 83 | 41,133 | 1.306 | 0.321, 5.323 | 0.002 | 0.708 |
| *CTCF* | 2 | 763 | 765 | Yes | Yes | No | 83 | 41,133 | 1.306 | 0.321, 5.323 | 0.002 | 0.708 |
| *RAC1* | 1 | 747 | 748 | Yes | Yes | Yes | 91 | 46,530 | 0.681 | 0.095, 4.894 | -0.002 | 0.701 |
| *SDHC* | 5 | 735 | 740 | Yes | Yes | No | 83 | 41,133 | 3.523 | 1.422, 8.728 | 0.014 | 0.004 |
| *MITF* | 0 | 734 | 734 | Yes | Yes | No | 83 | 41,133 | 0.000 | – | -0.006 | 0.219 |
| *IRF2* | 2 | 719 | 721 | Yes | Yes | No | 83 | 41,133 | 1.388 | 0.341, 5.656 | 0.002 | 0.646 |
| *MTAP, CDKN2A* | 0 | 708 | 708 | Yes | No | Yes | 85 | 40,419 | 0.000 | – | -0.006 | 0.218 |
| *CDC73* | 2 | 700 | 702 | Yes | Yes | No | 83 | 41,133 | 1.426 | 0.350, 5.812 | 0.002 | 0.619 |
| *HNF1A* | 1 | 695 | 696 | Yes | Yes | No | 83 | 41,133 | 0.710 | 0.099, 5.105 | -0.002 | 0.732 |
| *RAF1* | 1 | 694 | 695 | Yes | Yes | Yes | 91 | 46,530 | 0.734 | 0.102, 5.274 | -0.001 | 0.758 |
| *MAP2K1* | 0 | 689 | 689 | Yes | Yes | Yes | 91 | 46,530 | 0.000 | – | -0.005 | 0.242 |
| *SMAD2* | 1 | 682 | 683 | Yes | Yes | No | 83 | 41,133 | 0.723 | 0.101, 5.204 | -0.002 | 0.747 |
| *PRKAR1A* | 1 | 676 | 677 | Yes | Yes | No | 83 | 41,133 | 0.730 | 0.101, 5.251 | -0.002 | 0.753 |
| *GID4* | 3 | 666 | 669 | Yes | Yes | No | 83 | 41,133 | 2.279 | 0.718, 7.233 | 0.007 | 0.151 |
| *MKNK1* | 0 | 648 | 648 | Yes | Yes | No | 83 | 41,133 | 0.000 | – | -0.006 | 0.249 |
| *SMARCB1* | 0 | 642 | 642 | Yes | Yes | Yes | 91 | 46,530 | 0.000 | – | -0.005 | 0.259 |
| *NFKBIA* | 0 | 632 | 632 | Yes | Yes | No | 83 | 41,133 | 0.000 | – | -0.006 | 0.255 |
| *PDK1* | 2 | 629 | 631 | Yes | Yes | No | 83 | 41,133 | 1.590 | 0.390, 6.481 | 0.003 | 0.514 |
| *PTPN11* | 0 | 629 | 629 | Yes | Yes | No | 83 | 41,133 | 0.000 | – | -0.006 | 0.256 |

| *CD274* | 0 | 626 | 626 | Yes | Yes | Yes | 91 | 46,530 | 0.000 | – | -0.005 | 0.265 |
| --- | --- | --- | --- | --- | --- | --- | --- | --- | --- | --- | --- | --- |
| *ALOX12B* | 1 | 623 | 624 | Yes | Yes | No | 83 | 41,133 | 0.793 | 0.110, 5.706 | -0.001 | 0.817 |
| *EED* | 1 | 621 | 622 | Yes | Yes | No | 83 | 41,133 | 0.796 | 0.111, 5.725 | -0.001 | 0.82 |
| *ERG* | 1 | 618 | 619 | Yes | Yes | No | 83 | 41,133 | 0.799 | 0.111, 5.753 | -0.001 | 0.824 |
| *VHL* | 1 | 615 | 616 | Yes | Yes | Yes | 91 | 46,530 | 0.830 | 0.115, 5.963 | -0.001 | 0.852 |
| *CUL3* | 2 | 611 | 613 | Yes | Yes | Yes | 91 | 46,530 | 1.689 | 0.415, 6.874 | 0.003 | 0.459 |
| *BTG2* | 2 | 601 | 603 | Yes | Yes | No | 83 | 41,133 | 1.665 | 0.408, 6.788 | 0.004 | 0.472 |
| *PDCD1* | 2 | 600 | 602 | Yes | Yes | No | 83 | 41,133 | 1.668 | 0.409, 6.800 | 0.004 | 0.471 |
| *SDHD* | 4 | 591 | 595 | Yes | Yes | No | 83 | 41,133 | 3.473 | 1.268, 9.515 | 0.013 | 0.01 |
| *STAT3* | 2 | 591 | 593 | Yes | Yes | Yes | 91 | 46,530 | 1.747 | 0.429, 7.110 | 0.004 | 0.43 |
| *HRAS* | 1 | 592 | 593 | Yes | Yes | Yes | 91 | 46,530 | 0.862 | 0.120, 6.198 | -0.001 | 0.883 |
| *FOXL2* | 1 | 591 | 592 | Yes | Yes | No | 83 | 41,133 | 0.837 | 0.116, 6.020 | -0.001 | 0.859 |
| *SYK* | 0 | 571 | 571 | Yes | Yes | No | 83 | 41,133 | 0.000 | – | -0.005 | 0.28 |
| *MEF2B* | 0 | 569 | 569 | Yes | Yes | No | 83 | 41,133 | 0.000 | – | -0.005 | 0.281 |
| *H3F3A* | 1 | 551 | 552 | Yes | Yes | No | 83 | 41,133 | 0.898 | 0.125, 6.465 | -0.001 | 0.915 |
| *CD79B* | 1 | 549 | 550 | Yes | Yes | No | 83 | 41,133 | 0.902 | 0.125, 6.488 | -0.001 | 0.918 |
| *ERRFI1* | 0 | 547 | 547 | Yes | Yes | No | 83 | 41,133 | 0.000 | – | -0.005 | 0.29 |
| *U2AF1* | 0 | 546 | 546 | Yes | Yes | No | 83 | 41,133 | 0.000 | – | -0.005 | 0.291 |
| *GNA13* | 0 | 537 | 537 | Yes | Yes | No | 83 | 41,133 | 0.000 | – | -0.005 | 0.295 |
| *CRKL* | 1 | 532 | 533 | Yes | Yes | Yes | 91 | 46,530 | 0.961 | 0.134, 6.908 | -0.000 | 0.968 |

| *REL* | 1 | 525 | 526 | Yes | Yes | No | 83 | 41,133 | 0.943 | 0.131, 6.790 | -0.000 | 0.954 |
| --- | --- | --- | --- | --- | --- | --- | --- | --- | --- | --- | --- | --- |
| *SUFU* | 1 | 521 | 522 | Yes | Yes | No | 83 | 41,133 | 0.951 | 0.132, 6.843 | -0.000 | 0.96 |
| *IRF4* | 1 | 509 | 510 | Yes | Yes | No | 83 | 41,133 | 0.973 | 0.135, 7.006 | -0.000 | 0.979 |
| *PPARG* | 0 | 507 | 507 | Yes | Yes | No | 83 | 41,133 | 0.000 | – | -0.005 | 0.309 |
| *RAD51* | 0 | 504 | 504 | Yes | Yes | No | 83 | 41,133 | 0.000 | – | -0.005 | 0.31 |
| *GSK3B* | 2 | 502 | 504 | Yes | Yes | No | 83 | 41,133 | 1.998 | 0.490, 8.150 | 0.005 | 0.325 |
| *PIK3CA, PTEN* | 0 | 503 | 503 | Yes | Yes | Yes | 91 | 46,530 | 0.000 | – | -0.005 | 0.319 |
| *XPO1* | 2 | 498 | 500 | Yes | Yes | No | 83 | 41,133 | 2.015 | 0.494, 8.217 | 0.005 | 0.319 |
| *NT5C2* | 1 | 498 | 499 | Yes | Yes | Yes | 91 | 46,530 | 1.027 | 0.143, 7.385 | 0.000 | 0.979 |
| *AURKB* | 2 | 493 | 495 | Yes | Yes | No | 83 | 41,133 | 2.035 | 0.499, 8.302 | 0.005 | 0.312 |
| *CHEK1* | 3 | 482 | 485 | Yes | Yes | No | 83 | 41,133 | 3.163 | 0.995, 10.050 | 0.010 | 0.039 |
| *FAS* | 1 | 454 | 455 | Yes | Yes | No | 83 | 41,133 | 1.093 | 0.152, 7.867 | 0.000 | 0.93 |
| *CD79A* | 0 | 445 | 445 | Yes | Yes | No | 83 | 41,133 | 0.000 | – | -0.005 | 0.341 |
| *PIM1* | 0 | 441 | 441 | Yes | Yes | No | 83 | 41,133 | 0.000 | – | -0.005 | 0.343 |
| *JUN* | 2 | 439 | 441 | Yes | Yes | No | 83 | 41,133 | 2.289 | 0.561, 9.338 | 0.006 | 0.235 |
| *TENT5C* | 0 | 423 | 423 | Yes | Yes | No | 83 | 41,133 | 0.000 | – | -0.005 | 0.353 |
| *BTK* | 0 | 418 | 418 | Yes | Yes | No | 83 | 41,133 | 0.000 | – | -0.005 | 0.356 |
| *CYP17A1* | 0 | 413 | 413 | Yes | Yes | No | 83 | 41,133 | 0.000 | – | -0.005 | 0.359 |
| *GNAQ* | 0 | 405 | 405 | Yes | Yes | Yes | 91 | 46,530 | 0.000 | – | -0.004 | 0.371 |
| *MAPK1* | 0 | 403 | 403 | Yes | Yes | No | 83 | 41,133 | 0.000 | – | -0.004 | 0.365 |

| *XRCC2* | 1 | 402 | 403 | Yes | Yes | No | 83 | 41,133 | 1.236 | 0.172, 8.899 | 0.001 | 0.833 |
| --- | --- | --- | --- | --- | --- | --- | --- | --- | --- | --- | --- | --- |
| *CDKN1A* | 0 | 396 | 396 | Yes | Yes | No | 83 | 41,133 | 0.000 | – | -0.004 | 0.369 |
| *QKI* | 2 | 389 | 391 | Yes | Yes | No | 83 | 41,133 | 2.586 | 0.634, 10.556 | 0.007 | 0.169 |
| *P2RY8* | 1 | 367 | 368 | Yes | Yes | No | 83 | 41,133 | 1.355 | 0.188, 9.758 | 0.001 | 0.762 |
| *HDAC1* | 2 | 365 | 367 | Yes | Yes | No | 83 | 41,133 | 2.758 | 0.676, 11.259 | 0.007 | 0.14 |
| *BCL2L2* | 0 | 361 | 361 | Yes | Yes | No | 83 | 41,133 | 0.000 | – | -0.004 | 0.391 |
| *CBFB* | 2 | 342 | 344 | Yes | Yes | No | 83 | 41,133 | 2.945 | 0.721, 12.026 | 0.008 | 0.114 |
| *CXCR4* | 0 | 308 | 308 | Yes | Yes | No | 83 | 41,133 | 0.000 | – | -0.004 | 0.429 |
| *PPP2R2A* | 0 | 301 | 301 | Yes | Yes | No | 83 | 41,133 | 0.000 | – | -0.004 | 0.434 |
| *SOCS1* | 0 | 293 | 293 | Yes | Yes | No | 83 | 41,133 | 0.000 | – | -0.004 | 0.44 |
| *GNA11* | 0 | 287 | 287 | Yes | Yes | Yes | 91 | 46,530 | 0.000 | – | -0.003 | 0.452 |
| *SDHB* | 0 | 282 | 282 | Yes | Yes | No | 83 | 41,133 | 0.000 | – | -0.004 | 0.449 |
| *MYD88* | 1 | 281 | 282 | Yes | Yes | No | 83 | 41,133 | 1.773 | 0.246, 12.782 | 0.003 | 0.565 |
| *CD70* | 1 | 267 | 268 | Yes | Yes | No | 83 | 41,133 | 1.867 | 0.259, 13.459 | 0.003 | 0.529 |
| *NRG1* | 2 | 265 | 267 | Yes | No | Yes | 85 | 40,419 | 3.651 | 0.893, 14.921 | 0.010 | 0.053 |
| *TNFRSF14* | 1 | 261 | 262 | Yes | Yes | No | 83 | 41,133 | 1.910 | 0.265, 13.772 | 0.003 | 0.514 |
| *ARID2* | 1 | 252 | 253 | Yes | No | Yes | 85 | 40,419 | 1.898 | 0.263, 13.682 | 0.003 | 0.518 |
| *NPM1* | 0 | 240 | 240 | Yes | Yes | No | 83 | 41,133 | 0.000 | – | -0.003 | 0.485 |
| *CDKN2C* | 1 | 234 | 235 | Yes | Yes | No | 83 | 41,133 | 2.131 | 0.295, 15.378 | 0.004 | 0.442 |
| *SETBP1* | 1 | 226 | 227 | No | No | Yes | 8 | 5,397 | 3.269 | 0.400, 26.681 | 0.016 | 0.242 |

| *KRAS, TP53* | 0 | 220 | 220 | Yes | Yes | Yes | 91 | 46,530 | 0.000 | – | -0.003 | 0.511 |
| --- | --- | --- | --- | --- | --- | --- | --- | --- | --- | --- | --- | --- |
| *MAP3K4* | 0 | 192 | 192 | Yes | No | Yes | 85 | 40,419 | 0.000 | – | -0.003 | 0.524 |
| *BCL2* | 0 | 191 | 191 | Yes | Yes | No | 83 | 41,133 | 0.000 | – | -0.003 | 0.534 |
| *BTG1* | 1 | 180 | 181 | Yes | Yes | No | 83 | 41,133 | 2.775 | 0.384, 20.042 | 0.005 | 0.291 |
| *TMPRSS2-*  *ERG* fusion | 2 | 173 | 175 | Yes | Yes | No | 83 | 41,133 | 5.846 | 1.426, 23.966 | 0.014 | 0.005 |
| *IL7R* | 0 | 140 | 140 | Yes | No | Yes | 85 | 40,419 | 0.000 | – | -0.003 | 0.587 |
| *PIK3CA, ERBB2* | 1 | 135 | 136 | Yes | Yes | Yes | 91 | 46,530 | 3.819 | 0.528, 27.604 | 0.007 | 0.153 |

MSI, microsatellite instability; TMB, tumor mutational burden
