# Supplementary material for: Prevalence of neurotrophic tropomyosin receptor kinase (NTRK) fusion gene positivity in patients with solid tumors in Japan
Source: Cancer Med. 2024 Jun 25;13(12):e7351. doi: 10.1002/cam4.7351 (PMC11199329; doi:10.1002/cam4.7351)
Supplement: Supplementary file 7 — Table S7. [file CAM4-13-e7351-s004.docx]

| Table S7. (A) Proportion of NTRK gene fusions in adults (≥18 years) by histological classification | | | | |
| --- | --- | --- | --- | --- |
| Cancer Type | Histological type | Total number of patients | NTRK fusion, n (%) | |
| Head and Neck | All | 1668 | 21 | 1.26% |
|  | Acinic Cell Carcinoma | 18 | 3 | 16.67% |
|  | Mammary Analog Secretory Carcinoma of Salivary Gland Origin | 9 | 9 | 100.00% |
|  | Myoepithelial Carcinoma | 26 | 1 | 3.85% |
|  | Salivary Adenocarcinoma | 29 | 1 | 3.45% |
|  | Salivary Carcinoma Unclassified | 36 | 1 | 2.78% |
|  | Unclassified | 499 | 6 | 1.20% |
| Soft Tissue | All | 1919 | 11 | 0.57% |
|  | Angiomatoid Fibrous Histiocytoma | 1 | 1 | 100.00% |
|  | Angiosarcoma | 106 | 1 | 0.94% |
|  | Dedifferentiated Liposarcoma | 187 | 1 | 0.53% |
|  | Well-Differentiated Liposarcoma | 30 | 1 | 3.33% |
|  | Ewing Sarcoma of Soft Tissue | 24 | 1 | 4.17% |
|  | Gastrointestinal Stromal Tumor | 166 | 1 | 0.60% |
|  | Sarcoma, NOS | 81 | 2 | 2.47% |
|  | Unclassified | 549 | 3 | 0.55% |
| Thyroid | All | 529 | 7 | 1.32% |
|  | Papillary Thyroid Cancer | 223 | 6 | 2.69% |
|  | Unclassified | 92 | 1 | 1.09% |
| (B) Proportion of NTRK gene fusions in pediatric patients (<18 years) by histological classification | | | | |
| Cancer Type | Histological type | Total number of patients | NTRK fusion, n (%) | |
| Head and Neck | All | 10 | 1 | 10.00% |
|  | Mammary Analog Secretory Carcinoma of Salivary Gland Origin | 1 | 1 | 100.00% |
| Soft Tissue | All | 158 | 8 | 5.06% |
|  | Infantile Fibrosarcoma | 6 | 3 | 50.00% |
|  | Sarcoma, NOS | 15 | 1 | 6.67% |
|  | Unclassified | 47 | 4 | 8.51% |
|  |  |  |  |  |
